# Fibroblast PI3K/AKT signaling and extracellular matrix homeostasis: mechanisms, targets, and delivery challenges

**DOI:** 10.3389/fcell.2025.1681875

**Published:** 2025-12-23

**Authors:** Chunyun Fang, Zitao Zeng, Bin Ni, Xiaochun Wen, Zhipeng Fang, Junrong Zou, Guoxi Zhang

**Affiliations:** 1 Department of Obstetrics and Gynecology, First Affiliated Hospital of Gannan Medical University, Ganzhou, Jiangxi, China; 2 First Clinical College of Medicine, Gannan Medical University, Ganzhou, Jiangxi, China; 3 Department of Pharmacy, First Affiliated Hospital of Gannan Medical University, Ganzhou, Jiangxi, China; 4 Department of Radiology, Wan’an County People’s Hospital, Ji’an, Jiangxi, China; 5 Department of Urology, Institute of Urology, First Affiliated Hospital of Gannan Medical University, Jiangxi Engineering Technology Research Center of Calculi Prevention, Gannan Medical University, Ganzhou, Jiangxi, China

**Keywords:** cell therapy, extracellular matrix, fibroblast, PI3K/AKT, targeted therapy

## Abstract

The extracellular matrix (ECM) is essential for tissue homeostasis, ensuring structural stability, facilitating cell-cell communication, and tightly controlling key cellular processes, including proliferation, differentiation, and migration. Numerous cell types and signalling cascades direct ECM turnover; chief among them, the phosphatidyl-inositol-3-kinase (PI3K)/AKT (protein kinase B, PKB) axis remains intensively studied in fibroblasts. Recent evidence indicates that the integration of extracellular cues with intracellular mediators in fibroblasts can modulate the impact of the PI3K/AKT pathway on the ECM. This process is intricately linked to critical fibroblast functions such as metabolic reprogramming, autophagy, apoptosis, and stress responses, ultimately shaping outcomes in fibrotic diseases, wound healing, tissue remodelling, and pathological scar formation. Whereas conventional reviews centre on site-restricted subsets in single disorders, we integrate multi-tissue insights to chart PI3K/AKT signalling across heterogeneous fibroblast populations, taxonomising their sources into a unifying framework that confronts heterogeneity and accelerates precision therapeutic design.

## 1 Introduction

The extracellular matrix (ECM) is a dynamic, three-dimensional network that emerges during embryogenesis and persists across diverse tissues and organs. It sustains vital biological functions by ensuring structural integrity, conferring mechanical elasticity, and preserving tissue homeostasis via tightly regulated synthesis and degradation (Hynes, 2009). Functioning as a structural scaffold, the ECM mediates tissue remodeling by bridging the extracellular milieu with intracellular signaling, directing cellular processes like proliferation, differentiation, and migration, and critically shaping resident cell behavior (Theocharis et al., 2016). Collagen constitutes the ECM’s primary protein, accompanied by fibrous proteins (elastin, fibronectin (FN), laminin) and glycosaminoglycan-rich components like hyaluronic acid (HA). Beyond development, the ECM underpins tissue homeostasis, facilitating wound repair (Talbott et al., 2022), pelvic floor stabilization in stress incontinence (Fang et al., 2025), and airway regeneration (Liu G. et al., 2021), among others. It further governs joint inflammation, angiogenesis, and immune/cancer cell trafficking (Bonnans, Chou, and Werb, 2014). Conversely, dysregulation of ECM composition can precipitate various pathologies by altering tissue biomechanics and the biochemical microenvironment, including fibrotic diseases, Graves’ ophthalmopathy (GO) characterized by HA deposition (Lanzolla, Marinò, and Menconi, 2024), and severe conditions like systemic sclerosis (SSc) (Piera-Velazquez and Jimenez, 2021). Fibrosis, a slowly progressive disorder, leads to functional impairment across cellular, tissue, and organ levels, culminating in tissue degeneration (Conte, 2022). In summary, the ECM is a complex and dynamic structure that is crucial for intercellular communication. As its regulation involves a multitude of cell types, this article will primarily explore these mechanisms from the perspective of fibroblasts.

The term “fibroblast” broadly encompasses connective tissue-producing cells, including mesenchymal cells, perivascular cells, stromal progenitors, and classical fibroblasts (Younesi et al., 2024). For the purposes of this review, we specifically focus on *bona fide* fibroblasts. As mesenchymal-derived cells of embryonic mesodermal origin, fibroblasts serve as pivotal regulators of ECM homeostasis and are ubiquitously distributed throughout human tissues. Under normal physiological conditions, fibroblasts contribute to tissue maintenance by synthesizing ECM components (collagen, elastin, proteoglycans), modulating immune responses, and preserving tissue architecture. However, pathological activation triggers excessive matrix protein synthesis, causing ECM accumulation, matrix metalloproteinases (MMPs) and tissue inhibitors of metalloproteinases (TIMPs) imbalance, and ultimately fibrotic tissue remodeling. Disrupted MMPs/TIMPs ratios promote aberrant ECM degradation, compromising tissue integrity (Frangogiannis, 2021; Bonnans, Chou, and Werb, 2014). Fibroblast-secreted MMP-2 and MMP-9 (MMP-2/9) mediate degradation of key ECM components, including collagen, FN, gelatin, and elastin. Following tissue injury, fibroblasts undergo phenotypic conversion to myofibroblasts in response to mechanical stress, chemokine signaling, and inflammatory cues. Myofibroblasts are primarily localized to pathological milieus, such as damaged, fibrotic, and neoplastic tissues. These cells are typically characterized by the expression of alpha-smooth muscle actin (α-SMA) and are responsible for secreting excessive collagen and FN, alongside key pro-fibrotic mediators like transforming growth factor-β (TGF-β), which collectively drive disease progression. Under physiological conditions, such as in wound resolution, myofibroblasts are efficiently eliminated via apoptosis, inactivation, or integration into the scar tissue. In contrast, during persistent pathology, they evade apoptotic clearance and maintain a chronically activated state, resulting in aberrant tissue remodeling and irreversible damage (Travers et al., 2016; Witherel et al., 2019). This dynamic process, known as the fibroblast-to-myofibroblast transition (FMT), thus constitutes a fundamental mechanism of tissue repair that, when dysregulated, becomes a pivotal driver of pathological fibrosis.

The phosphoinositide 3-kinase (PI3K)/protein kinase B (AKT) signaling pathway represents a pivotal intracellular axis, critically governing fundamental biological processes including cell proliferation, survival, metabolism, migration, and apoptosis. Numerous studies and reviews have elucidated PI3K/AKT-mediated mechanisms in diseases such as cancer (Hao et al., 2025; Hashemi et al., 2023; Yu X et al., 2022), inflammatory conditions (Chen et al. 201b; Sun F. et al., 2020), neurological disorders (Guo et al., 2024), and fibrotic diseases (Wang J. et al., 2022). However, most existing research focuses on disease-specific PI3K/AKT pathways, with limited attention given to cell-centric perspectives. To address ECM dysregulation in fibrotic and remodeling diseases, we propose a fibroblast-PI3K/AKT-centric framework to integrate evidence from various fibrotic diseases, wound healing, and scar formation processes. Although research on PI3K/AKT mechanisms in fibroblasts remains developing, this review synthesizes recent advances in the fibroblast-PI3K/AKT-ECM axis to establish a theoretical foundation for future investigations.

**FIGURE 1:**
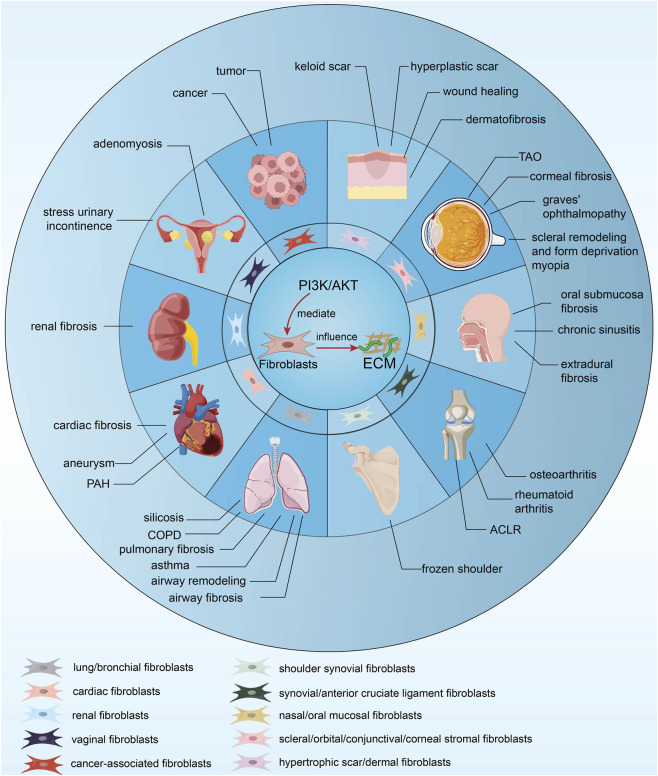
This figure provides a comprehensive overview of PI3K/AKT-mediated ECM regulation within fibroblast populations. It synthesizes recent evidence on the pathophysiological processes driven by the fibroblast-PI3K/AKT-ECM axis—notably fibrosis and tissue remodeling—across diverse human organ systems, including the heart, kidney, lung, skin, eye, joints, and musculature. This synthesis establishes a conceptual framework of diseases associated with this signaling axis, thereby laying the necessary groundwork for the subsequent, more detailed discussions that follow.

Overall, the mechanisms through which fibroblasts modulate the ECM are intricate and multifaceted, encompassing a variety of signaling pathways. Herein, we initially delineate the fundamental association between fibroblast characteristics and PI3K/AKT signaling. Subsequently, leveraging the multifunctional nature of PI3K/AKT, we examine its role and regulatory mechanisms in the ECM during disease processes in diverse tissues. These diseases encompass fibrotic conditions, including pulmonary, cardiac, renal, cutaneous, extracranial, and shoulder fibrosis, along with abnormal scarring. Moreover, we address diseases associated with ECM homeostasis, such as pulmonary arterial hypertension (PAH), wound healing, stress urinary incontinence, airway remodeling, and GO. The focus is on the pathophysiological responses of fibroblasts mediated by PI3K/AKT in fibrosis and tissue remodeling, as well as potential therapeutic targets for modulating fibroblast PI3K/AKT signaling. Finally, informed by recent preclinical studies, we summarize potential targeted therapeutic agents, propose drug delivery strategies, and outline future perspectives (Figure 1).

## 2 Overview of fibroblasts and their functional association with the PI3K/AKT signaling pathway

### 2.1 Origin and functions of fibroblasts

The historical characterization of fibroblasts dates to 1858, when Rudolf Virchow first described them as “spindle-shaped cells of connective tissue” (Molenaar, 2003), a definition later formalized by Ernst Ziegler who introduced the term “fibroblast” (Ziegler, 1900). The developmental origin of these cells, as comprehensively reviewed by LeBleu and Neilson (2020), is traced to embryonic gastrulation. During this pivotal stage, ectodermal cells undergo a type I epithelial-mesenchymal transition (EMT) to generate primitive mesenchyme, which differentiates into primitive fibroblasts (PF). This primitive mesenchyme gives rise to the endoderm and mesoderm, with the latter differentiating into mature fibroblasts, connective tissue, and other structures. Mesenchymal stromal cells (MSCs), which share a mesodermal origin, persist in adult tissues as phenotypic analogues or precursors to resident fibroblasts. The fibroblasts derived from these embryonic primordial cells ultimately colonize tissue interstitium, establishing the population of resident quiescent fibroblasts (RQFs) that maintain ECM homeostasis in healthy states. Furthermore, fibroblasts exhibit significant transcriptional heterogeneity across different anatomical sites, a phenomenon governed by positional memory and the regulation of axis-specific patterning genes (LeBleu and Neilson, 2020). Notably, the diverse origins of fibroblasts underpin their functional heterogeneity, determine differentiation pathways, and influence plasticity. These manifestations include embryonic PFs contributing to tissue architecture and ECM remodeling; adult RQFs maintaining ECM homeostasis and facilitating repair post-injury; EMT-transdifferentiated fibroblasts and bone marrow-derived fibroblasts performing specialized roles in fibrosis, injury repair, and immune regulation; organ-specific subtypes such as pulmonary adipose-derived fibroblasts and hepatic stellate cells (HSCs) adapting to their respective organ functions; and pathological subpopulations like cancer-associated fibroblasts (CAFs) exhibiting heterogeneous functions due to their diverse origins (LeBleu and Neilson, 2020).

**FIGURE 2:**
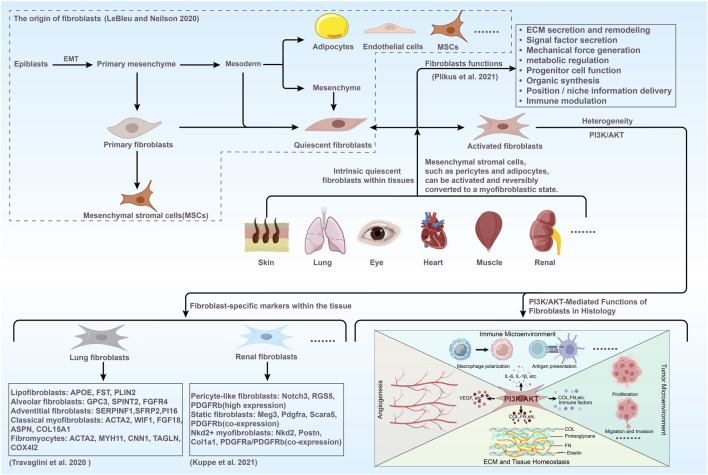
The schematic diagram offers a comprehensive overview of the origins of fibroblasts and their multifaceted roles within the organism. It also underscores the characteristic markers of heterogeneous fibroblasts in various tissues (including pulmonary fibroblasts (Travaglini et al., 2020) and renal fibroblasts (Kuppe et al., 2021)), their activation, and functions regulated by the PI3K/AKT pathway. A number of fibroblast-specific markers are listed.

Plikus et al. (2021) have systematically delineated the multifaceted functions of fibroblasts into eight principal categories: (1) ECM secretion and remodeling: Fibroblasts secrete ECM components, including collagen, elastic fibers, and proteoglycans, and regulate ECM architecture via cross-linking and proteolysis to preserve tissue mechanical strength and microenvironmental stability. (2) Signaling molecule Secretion: Fibroblasts release soluble signaling molecules, including cytokines and growth factors, which provide regulatory signals and participate in processes such as cell proliferation and differentiation. (3) Generation of mechanical forces: Fibroblasts generate mechanical forces via self-contraction, driving ECM polarization and tissue morphogenesis, and facilitating repair processes such as wound contraction. (4) Metabolic regulation: Fibroblasts respond to environmental signals (e.g., hypoxia), modulate metabolic pathways (e.g., glycolysis), secrete metabolic byproducts, and contribute to tissue energy balance and microenvironmental metabolic regulation. (5) Progenitor cell Function: Acting as self-renewing mesenchymal progenitors, they maintain population stability and support tissue repair through differentiation. (6) Tissue synthesis: During organ morphogenesis, tissue injury repair, and pathological states, fibroblasts construct the tissue matrix (e.g., basement membranes), thereby providing the structural foundation for tissue regeneration. (7) Niche signaling: Fibroblasts provide positional information to tissues via region-specific molecular expression and serve as a key signaling hub within the stem cell niche, regulating stem cell quiescence and activation. (8) Immune regulation: Fibroblasts bidirectionally modulate innate and adaptive immunity, including regulating macrophage polarization, participating in parasite encapsulation and foreign body clearance, and secreting antimicrobial peptides to enhance tissue resistance to infection (Figure 2).

### 2.2 Fibroblast heterogeneity, plasticity, and their functional integration with the PI3K/AKT signaling pathway

Fibroblast heterogeneity, which arises from their diverse developmental origins, is further complicated by the current lack of a fully defined, unique molecular marker panel (Younesi et al., 2024). Consequently, detecting fibroblasts can be challenging due to the nonspecificity of many known markers. Examples include vimentin, collagen 1α2 chain (COL1α2), and platelet-derived growth factor receptor alpha (PDGFRα), which are expressed in most fibroblasts and also detected in some endothelial and epithelial cells (Driskell and Watt, 2015). To understand the complex role of the PI3K/AKT pathway in fibrosis, it is crucial to recognize that fibroblasts are a heterogeneous population. Their intrinsic heterogeneity, significant functional plasticity, and diverse activation states collectively determine their responsiveness to PI3K/AKT signaling. In recent years, single-cell RNA sequencing (scRNA-seq) has enhanced our understanding of fibroblast heterogeneity. Researchers have gained deeper insights into common and specific markers of fibroblasts across and within organs, and identified the functions associated with different subtypes based on these markers (Lendahl et al., 2022). For instance, in renal tissue, Nkd2^+^myofibroblasts directly govern collagen production, whereas Colec11^+^/Cxcl12^+^ fibroblasts establish a pro-inflammatory microenvironment, with their expansion promoting inflammation-driven fibrogenesis. Their numbers significantly increase during fibrosis progression, driving inflammation-related fibrotic development (Kuppe et al., 2021). Crucially, fibroblast multi-subtyping and multifunctionality suggest fundamental differences in basal signaling activity and dependencies among distinct subpopulations. Within the PI3K/AKT context, the pro-fibrotic subpopulation may exhibit higher basal PI3K/AKT activity than resting subpopulations, making it more sensitive to stimuli and prone to ECM secretion. Conversely, a metabolically active subpopulation may depend more heavily on the PI3K/AKT pathway for energy supply. In summary, fibroblast responses to PI3K/AKT signaling are not uniform but are deeply rooted in their intrinsic properties.

The activation and plasticity of fibroblasts are also central targets of PI3K/AKT regulation. FMT is the quintessential manifestation of their functional plasticity (Zhang F et al., 2025), driven by complex signaling networks in which the PI3K/AKT pathway acts as a central integrator, engaging in extensive cross-talk with diverse activating signals. TGF-β, which will be detailed later, activates fibroblasts via both the canonical SMAD pathway and the non-canonical PI3K/AKT pathway. These pathways synergistically promote fibroblast survival and inhibit apoptosis, leading to the sustenance of myofibroblast persistence. Furthermore, pathways like Wnt/β-catenin intersect with PI3K/AKT (Sharma et al., 2002; Yu et al., 2018), co-regulating fibroblast proliferation and metabolic reprogramming. Importantly, activated fibroblasts have heterogeneous origins, including tissue-resident fibroblasts, mesenchymal precursor cells, and transdifferentiated epithelial or endothelial cells (Younesi et al., 2024). These cells of diverse origins may enter the myofibroblast pool with inherent signaling characteristics, further complicating PI3K/AKT signaling responses. Consequently, fibroblast subpopulations exhibit highly differential responses to PI3K/AKT pathway modulation. This heterogeneity implies that a universal therapeutic strategy targeting PI3K/AKT is unlikely to be effective against all pathogenic subpopulations and may disrupt subsets with protective functions. Therefore, elucidating these nuanced signaling dependencies presents a considerable challenge of paramount importance. This review aims to establish a preliminary theoretical framework to guide future research in this complex area.

In summary, the PI3K/AKT signaling pathway is a master regulator of fibroblast physiology and pathology, orchestrating critical processes including proliferation, migration, adhesion, metabolism, apoptosis, and ECM remodeling. Through dynamic phosphorylation cascades, it integrates diverse signals from growth factors, the ECM, and the tissue microenvironment to coordinate a spectrum of downstream biological events. The pathway promotes fibroblast proliferation and enhances cell survival by activating effectors like mTOR and concurrently inhibiting pro-apoptotic proteins such as Bax (Zhang X. et al., 2022). Furthermore, in pathological contexts like inflammation and cancer, PI3K/AKT signaling augments the fibroblast secretion of inflammatory mediators (e.g., IL-6, IL-1β) (Lee et al., 2021; Hou et al., 2021; Lin et al., 2021), drives the pro-tumorigenic reprogramming of CAFs (Fang et al., 2023; Zhou et al., 2018; Abdul-Wahid et al., 2018) and facilitates vascular endothelial growth factor (VEGF)-mediated angiogenesis (Wang FT et al., 2024; Jiang et al., 2024). These multifaceted roles underscore its significance as a pivotal therapeutic target for a range of fibroblast-driven diseases.

## 3 The PI3K/AKT signaling pathway regulates ECM metabolism by mediating the biological behavior of fibroblasts

The aforementioned findings have preliminarily established the close association between fibroblasts and PI3K/AKT signaling. We will first elaborate on the core characteristics of the PI3K/AKT pathway, then explore how alterations in fibroblast behavior mediated by this pathway influence the tissue ECM, thereby regulating pathological processes such as fibrosis and tissue remodeling.

### 3.1 Overview of the PI3K/AKT signaling pathway

PI3K is structurally and functionally classified into classes I, II, and III, with Class I further subdivided into IA and IB. Class IA PI3Ks exist as heterodimers composed of a catalytic subunit (p110α, p110β, or p110δ) and a regulatory subunit (e.g., p85α and its splice variants p55α and p50α, p85β, or p55γ) (Yu X. et al., 2022). Class IB PI3Ks are also heterodimers, with p110γ as the catalytic subunit and p101 or p84 as the regulatory subunit (Yu X. et al., 2022). The genes encoding these Class I PI3K catalytic subunits are PIK3CA (p110α), PIK3CB (p110β), PIK3CD (p110δ), and PIK3CG (p110γ) (Hoxhaj and Manning, 2020). Class II and III PI3K are primarily involved in intracellular membrane transport and exert indirect effects in PI3K signaling (Bilanges et al., 2019). Here, we focus on Class I. The regulatory subunits of Class I PI3K contain SH2 and SH3 domains, mediating specific recognition of phosphotyrosine-containing targets. Their activity is regulated primarily by receptor tyrosine kinases (RTKs) and G protein-coupled receptors (GPCRs). Upon activation, the p110 catalytic subunits convert phosphatidylinositol-4,5-diphosphate (PIP2) into phosphatidylinositol-3,4,5-trisphosphate (PIP3). PIP3 signaling is terminated primarily by the tumor suppressor PTEN, which dephosphorylates PIP3 back to PIP2. Loss of this function is closely associated with tumorigenesis (Glaviano et al., 2023). PIP3 activates AKT, a core effector molecule with three subtypes (AKT1-3). AKT can fully activate Thr308 phosphorylation via phosphoinositide-dependent kinase 1 (PDK1) and Ser473 phosphorylation via mTORC2 (He et al., 2021). Activated AKT can directly stimulate mTORC1 by phosphorylating mTOR at Ser2448 and indirectly enhance mTORC1 activity by inhibiting the TSC1/2 complex. Additionally, AKT regulates multiple downstream molecules. For instance, FOXO1, a member of the FOXO family of transcription factors, acts as a downstream effector of PI3K/AKT and can regulate PI3K activity via a feedback mechanism in human cardiac fibroblasts (HCFs) (Wang et al., 2018; Zhang X. et al., 2025). Interestingly, a recent study revealed that FOXO1 reduces ECM deposition by inhibiting PI3K/AKT signaling in HCFs, contradicting the conventional view that PI3K/AKT activation promotes ECM expression (Okada et al., 2015). This discrepancy may arise from tissue-specific differences in fibroblasts and multi-pathway crosstalk (Wang J. et al., 2022). FOXO3a, another member of the FOXO family and a downstream target of PI3K/AKT, mediates collagen synthesis-related genes COL1A1 and COL3A1 in cardiopulmonary tissues and promotes fibroblast proliferation (Ma et al., 2020; Zhang L. et al., 2023). FOXO represents a conserved family of transcription factors across multiple species, including FOXO1, FOXO3, FOXO4, and FOXO6 (Cheng, 2019). These act as homeostasis regulators, coordinating responses to growth factor deprivation, cellular metabolism, and stress to maintain tissue homeostasis in response to environmental changes (Calissi et al., 2021). Moreover, in apoptosis regulation, AKT directly phosphorylates the pro-apoptotic protein Bad at Ser136, promoting its dissociation from anti-apoptotic proteins Bcl-2/Bcl-xL and its retention in the cytoplasm. Consequently, the Bad-induced mitochondrial apoptosis pathway is blocked (Liu R. et al., 2020). Simultaneously, AKT inhibits glycogen synthase kinase 3β (GSK3β) activity by phosphorylating its N-terminal Ser9 site. This prevents GSK3β from phosphorylating and ubiquitinating β-catenin, allowing β-catenin to accumulate in the nucleus, where it collaborates with TCF/LEF transcription factors to activate Wnt target gene expression, driving cell proliferation and survival (Sharma, Chuang, and Sun, 2002; Lawrence and Nho, 2018; Mulholland et al., 2006). Through these integrated mechanisms, the PI3K/AKT pathway plays a pivotal role in pathophysiology.

**FIGURE 3:**
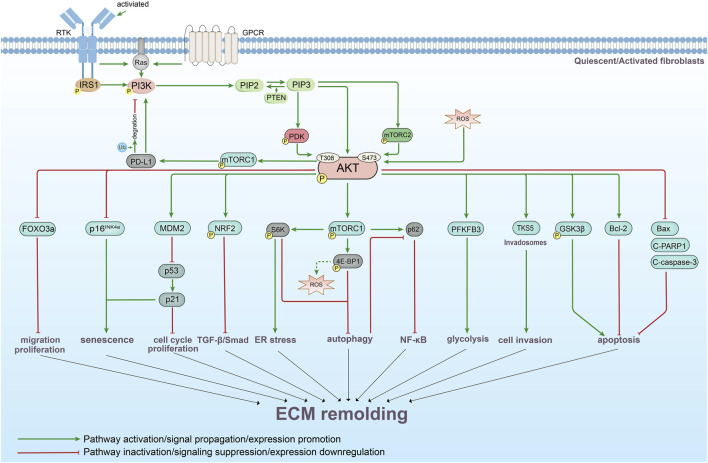
Summary of the mechanistic map of PI3K/AKT-mediated ECM in fibroblasts of various tissues. A simplified schematic diagram illustrating PI3K/AKT-mediated pathophysiological activities in fibroblasts, including proliferation, invasion, metabolism, autophagy, senescence, and apoptosis, and their subsequent effects on ECM remodeling. This diagram is intended to provide a general framework of the content.

Following tissue injury, the ensuing inflammatory response activates fibroblasts, initiating a cascade that culminates in aberrant ECM remodeling. Recent research has elucidated that the PI3K/AKT pathway is a central modulator of critical cellular processes in fibroblasts across various tissues, including their responses to oxidative stress (OS), endoplasmic reticulum (ER) stress, and the regulation of apoptosis, senescence, autophagy, metabolism, and invadosomes synthesis. By governing these diverse functions, PI3K/AKT signaling critically influences ECM dynamics and results in the progression of a wide spectrum of fibrotic and remodeling diseases (Figure 3).

### 3.2 PI3K/AKT signaling regulates the ECM by modulating OS in fibroblasts

OS is intricately linked to core metabolic processes such as the pentose phosphate pathway, fatty acid metabolism, and oxidative phosphorylation. It arises from a systemic imbalance between pro-oxidant and antioxidant species, leading to the excessive accumulation of reactive oxygen species (ROS) (Finkel and Holbrook, 2000). ROS generation occurs when nicotinamide adenine dinucleotide phosphate (NADPH) oxidase (NOX) transfers electrons from NADPH to molecular oxygen (Bedard and Krause, 2007), comprising oxygen free radicals such as superoxide and hydroxyl radicals, and non-radical oxygen derivatives such as hydrogen peroxide (H_2_O_2_) (Ornatowski et al., 2020). Under physiological conditions, ROS serve as vital signaling molecules and can mediate the action of cell-protective enzymes. However, when present in excess, ROS disrupt cellular metabolic equilibrium, and the resulting oxidative damage is a well-established driver of fibrotic pathogenesis across various organs (Todd et al., 2012).

The PI3K/AKT signaling pathway functions as a critical hub integrating OS with fibroblast activation and fibrotic progression. Stimulus-induced cellular OS modulates multiple signaling responses, including PI3K/AKT signaling (Sun et al., 2025). For example, cigarette extract stimulates excessive ROS production in nasal fibroblasts (NFs), disrupting the MMP-2/TIMP-2 balance by activating PI3K/AKT and nuclear factor-κB (NF-κB) signaling, resulting in the exacerbation of chronic rhinosinusitis (Park et al., 2020). Conversely, PI3K/AKT also influences OS responses (Wang Y. et al., 2025). In cardiac fibroblasts (CFs) overexpressing PI3K, NOX activity and superoxide anion levels significantly increase, whereas PI3K inhibition suppresses OS and improves cardiac fibrosis (Zhong et al., 2020). This indicates that PI3K/AKT and OS form a positive feedback loop, sustaining fibroblast activation. Within this positive feedback loop, NOX4 (a key enzymatic source of ROS) exhibits expression and activity regulated by PI3K/AKT signaling, closely linked to redox imbalance and ECM homeostasis (Chan et al., 2013; Kido et al., 2017; Park et al., 2010). A core cellular defense mechanism against OS involves the transcription factor nuclear factor E2-related factor 2 (NRF2) (Hybertson et al., 2011). Notably, a bidirectional regulatory relationship exists between PI3K/AKT and NRF2. On one hand, activated AKT directly phosphorylates NRF2, promoting its nuclear translocation in skeletal muscle fibroblasts (SMFs) and initiating transcription of target genes, including antioxidant enzymes, to maintain cellular redox homeostasis (Wu et al., 2023). Conversely, NRF2 can also feedback-inhibit PI3K/AKT signaling, which in turn limits excessive fibroblast activation. In pulmonary fibrosis, downregulation of NRF2 not only increases OS but also releases inhibition on PI3K/AKT signaling, accompanied by proliferation of lung fibroblasts (LFs) and excessive collagen secretion (Liu P. et al., 2021; Liu et al., 2013). Thus, within fibroblasts, the PI3K/AKT-NOX4-ROS axis drives activation, while the PI3K/AKT-NRF2 axis attempts to apply a brake. When NOX4-mediated pro-fibrotic effects overwhelm the regulatory capacity of defense systems like NRF2, and are accompanied by synergistic actions of other pro-fibrotic signals, this ultimately leads to excessive ECM deposition (Wang et al., 2021a). However, current experimental evidence for direct regulation of NOX4 expression or activity by PI3K/AKT signaling in fibroblasts remains relatively limited. Most studies support only a functional association or indirect regulation mediated by downstream transcription factors, while the specific mechanisms of their molecular interactions remain unclear (Kido et al., 2017; Park et al., 2010).

On the other hand, OS and inflammation are interrelated processes that play a crucial role in maintaining normal cellular homeostasis (Makena et al., 2023). This interplay significantly influences inflammatory signaling within fibroblasts; for instance, ROS activates the PI3K/AKT-NF-κB axis in human gingival fibroblasts, driving oral inflammation (Vo et al., 2021). In fact, inflammation is a natural response to external stimuli and oxidative stress, aiding tissue repair. Persistent inflammation is a primary driver of fibrotic disease progression (Nathan and Ding, 2010). In purely inflammatory conditions, excessive activation of the PI3K/AKT pathway in rheumatoid arthritis synovial fibroblasts (RASFs) may drive overproduction of IL-6 or IL-1β by stimulating NF-κB mechanisms (YangJ. et al., 2022). Similarly, PI3K/AKT pathway activation in osteoarthritis synovial fibroblasts (OASFs) regulates IL-1β, with integrin αVβ3 mediating cross-talk with this pathway, promoting IL-6 production and triggering inflammatory responses (Lee et al., 2021; Hou et al., 2021; Lin et al., 2021). In fibrosis, dysregulation of the PI3K/AKT pathway drives pulmonary fibroblast activation, confers anti-apoptotic properties, and promotes secretion of pro-inflammatory factors within the senescence-associated secretory phenotype (SASP), establishing a persistent inflammatory microenvironment. This chronic inflammation further drives excessive ECM deposition, ultimately leading to irreversible lung tissue scarring (Bhatt et al., 2025). Thus, the PI3K/AKT pathway-mediated inflammation-fibroblast activation-cytokine release axis is pivotal for fibrosis progression.

In conclusion, PI3K/AKT-mediated signaling forms a tightly integrated network at the OS level, governing both cellular redox signaling and inflammatory signaling. Together, these pathways shape the pathological process of fibroblast transformation toward a persistently activated, high-ECM-secreting phenotype.

### 3.3 PI3K/AKT signaling regulates the ECM by modulating apoptosis in fibroblasts

In fibrotic diseases, the abnormal persistence of fibroblasts is a prerequisite for pathological matrix deposition, while the PI3K/AKT signaling pathway, through its potent anti-apoptotic effects, is crucial for maintaining fibroblast survival. Apoptosis is a genetically regulated, active, and orderly process of cell death. It involves biochemical changes such as caspase activation, DNA fragmentation, and phosphatidylserine translocation, triggered through intrinsic mitochondrial and extrinsic death receptor pathways (Newton et al., 2024). Stress factors like ROS can induce apoptosis in dermal fibroblasts (DFs). However, activated PI3K/AKT can significantly inhibit apoptosis by phosphorylating downstream targets, thereby suppressing the activation of cleaved-caspase-3 (c-caspase-3), a core executor of apoptosis, and the cleavage of Poly (ADP-ribose) polymerase 1 (PARP1). This anti-apoptotic effect promotes the progression of the injury process toward repair (Yang R. et al., 2022). Caspase-3, belonging to the ICE-like protease family, is activated by mitochondrial cytochrome c to initiate caspase-9 activation. It is a common activated death protease in mammalian cell apoptosis, catalyzing the specific cleavage of multiple key cellular proteins (Porter and Jänicke, 1999). PARP is one of the cleavage targets following caspase-3 activation, and its cleavage serves as a crucial indicator of apoptosis (Chang et al., 2011).

PI3K/AKT regulates fibroblast apoptosis primarily via the mitochondrial pathway. On one hand, activated AKT phosphorylates and inhibits the pro-apoptotic protein Bad, rendering it inactive (Zhang et al., 2022a; Shiraishi et al., 2022). On the other hand, inhibiting PI3K/AKT in human keloid fibroblasts (HKFs) upregulates the pro-apoptotic protein Bax and downregulates the anti-apoptotic protein Bcl-2 (Zhi et al., 2021). This altered balance of Bcl-2 family proteins leads to increased mitochondrial membrane permeability, cytochrome c release, activation of initiator caspase-9 and effector caspase-3, ultimately triggering apoptosis. Moreover, in idiopathic pulmonary fibrosis (IPF), inhibiting AKT/GSK3β signaling significantly increases apoptosis rates in pulmonary fibroblasts (Bao et al., 2023). This indicates that the PI3K/AKT-Bcl-2/Bax-caspase-3 axis constitutes a core anti-apoptotic signaling pathway in fibroblasts, modulated by cell subtypes and microenvironmental signals.

Multiple fibrosis-associated factors influence fibroblast apoptosis by regulating this core axis. For example, transient receptor potential melastatin-subfamily member 7 (TRPM7), a unique channel-kinase protein permeable to numerous divalent cations and possessing an α-kinase with phosphorylated downstream substrates, plays a central role in cation homeostasis, cellular function, and organ development. When inhibited, TRPM7 promotes HKFs apoptosis by suppressing PI3K/AKT, thereby modulating the Bcl-2/Bax balance (Zhi et al., 2021; Chubanov et al., 2024). Similarly, in keloids, the transcription factor Runx2 acts as an upstream activator of PI3K/AKT. Knockdown of Runx2 induces fibroblast apoptosis and downregulates ECM protein expression by inhibiting PI3K/AKT signaling (Lv et al., 2021b). Furthermore, hypoxia, a key feature of the fibrotic microenvironment, stabilizes hypoxia-inducible factor α (HIFα) by activating the PI3K/AKT-mTOR pathway. Interestingly, however, hypoxia ultimately induces apoptosis in anterior cruciate ligament fibroblasts (ACLFs). Platelet-rich plasma (PRP), however, counteracts hypoxia-induced apoptosis by activating PI3K/AKT, thereby protecting cell survival, although it does not promote collagen synthesis (Cao et al., 2022). HIF is a heterodimeric transcription factor composed of α and β subunits that coordinates cellular adaptation to hypoxia by stabilizing its oxygen-sensitive α subunit under hypoxic conditions (Taylor and Scholz, 2022).

Notably, the anti-apoptotic effects of PI3K/AKT may synergize with calcium signaling pathways. Calcium ion (Ca^2+^) influx and the activation of its downstream effector, calmodulin-dependent kinase II (CaMKII), regulate fibroblast proliferation and migration (Imoto et al., 2018; Sazonova et al., 2007). More importantly, in pulmonary fibrosis models, simultaneous inhibition of both PI3K/AKT and CaMKII produces a stronger pro-apoptotic effect, significantly enhancing apoptosis in human lung fibroblasts (HLFs) and suppressing collagen expression—an effect superior to inhibiting either pathway alone (Zhao et al., 2020a). This strongly suggests functional synergy or cross-talk between PI3K/AKT and CaMKII signaling, collectively forming a network that maintains fibroblast survival. Although the specific molecular interface for this synergy remains incompletely elucidated, it offers a more promising dual-target strategy for intervening in fibroblast anti-apoptosis.

In summary, the PI3K/AKT pathway acts as a crucial regulator of fibroblast activity in apoptosis. It not only directly inhibits apoptosis via the Bcl-2/Caspase-3 axis but may also establish a more robust survival defense system through synergistic interactions with signals like CaMKII. This ensures the sustained survival of activated fibroblasts within the stressed microenvironment, establishing prerequisites for pathological ECM deposition and serving as a critical mechanism for maintaining and progressing fibrosis.

### 3.4 PI3K/AKT signaling regulates the ECM by modulating senescence in fibroblasts

Senescence is a permanent cell cycle arrest in response to stress. In the context of fibrosis, senescent fibroblasts play a complex and contradictory role. These cells exhibit persistent growth arrest, express anti-proliferative molecules such as p16^INK4a^, activate damage-sensing signaling pathways like p38 MAPK and NF-κB, and produce a series of senescence-associated products (He and Sharpless, 2017). Normal developmental senescence is characterized by low replicative capacity, independence from DNA damage, lack of reliance on p53 or p16^INK4a^ expression for proliferation arrest, and absence of typical SASP factor release (He and Sharpless, 2017; Muñoz-Espín et al., 2013). In contrast, senescent cells arising from tissue injury exhibit marked NF-κB activation and SASP cytokine expression (Baker et al., 2016; Demaria et al., 2014). The SASP comprises a complex array of components, including cytokines, MMPs, miRNAs, chemokines, growth factors, and small-molecule metabolites (Zhang et al., 2024). It reshapes the ECM by establishing chronic inflammation via cytokines and chemokines, suppressing growth factors, and activating hydrolases (Wlaschek et al., 2021), thus exerting multifaceted roles in aging. The PI3K/AKT signaling pathway serves as a critical switch in this process. By influencing core aging effectors, it determines whether fibroblasts undergo reparative quiescence or pathological senescence, ultimately regulating fibrosis progression.

Moderate activation of the PI3K/AKT pathway is crucial for maintaining fibroblast viability and counteracting aging. In human dermal fibroblasts (HDFs), the activated PI3K/AKT-mTOR signaling axis suppresses the expression of senescence-associated β-galactosidase (SA-β-Gal) and the release of pro-inflammatory factors such as IL-6. Simultaneously, it promotes the synthesis of collagen I/III (COL1/3) and elastin, thus maintaining ECM stability and elasticity and resisting skin aging (Guo et al., 2020). A key mechanism underlying this protective effect may involve PI3K/AKT’s inhibition of the critical aging regulatory axis p53/p21. As demonstrated in CFs, neuregulin 1 (Nrg1) activates the PI3K/AKT pathway by binding to its receptors, epidermal growth factor receptors 2 and 4 (ErbB2/4). This pathway subsequently upregulates MDM2, a negative regulator of p53, leading to p53 degradation and downregulation of p21 transcription. This process releases p21’s inhibition of cyclin-dependent kinase 2 (CDK2), significantly delaying CFs aging and alleviating cardiac fibrosis (Shiraishi et al., 2022). Therefore, the PI3K/AKT-MDM2/p53/p21 axis represents a unique anti-aging and pro-fibrotic pathway that sustains fibroblast proliferative activity and ECM synthesis capacity by inhibiting p53/p21-mediated senescence. However, under sustained or intense stress signals, abnormal activation of the PI3K/AKT pathway may induce or amplify pathological senescence phenotypes. In studies of skin photoaging, abnormal PI3K/AKT activation induced by ultraviolet (UV) exposure is closely associated with upregulation of the aging markers p53 and p21 (Chen Q. et al., 2022; Xu et al., 2025; Hu et al., 2025). This seemingly contradictory phenomenon reveals the dual-sided nature of PI3K/AKT signaling: physiologically, moderate activation promotes p53 degradation via MDM2 to suppress aging. Yet under persistent stress like UV exposure, pathway overactivation may counteract MDM2’s negative regulation of p53 by inducing DNA damage and accumulating ROS, thereby upregulating p53 and p21 and triggering pathological aging programs.

Furthermore, the PI3K/AKT pathway serves as a key mediator for senescent fibroblasts to exert their pro-fibrotic paracrine effects via SASP. In the irreversible IPF process, studies reveal that TGF-β-induced senescent pulmonary fibroblasts secrete abundant extracellular vesicles (EVs) carrying pregnancy-associated plasma protein A (PAPP-A). As a metalloproteinase, PAPP-A cleaves insulin-like growth factor-binding protein 4 (IGFBP-4), releasing and activating insulin-like growth factor (IGF). Activated IGF subsequently activates the PI3K/AKT signaling pathway in surrounding fibroblasts, driving anti-apoptotic responses and leading to severe ECM deposition. Collectively, this represents a paradigm of aging-driven fibrosis, where the TGF-β/PAPP-A/IGF-PI3K/AKT axis translates senescence-associated secretory protein (SASP) signals from senescent cells into potent PI3K/AKT-mediated pro-fibrotic signals, thereby amplifying the fibrotic response (Bale et al., 2022).

To summarize, at the cellular senescence level, the PI3K/AKT pathway serves as a key regulator determining the fate and function of fibroblasts. It delays senescence by inhibiting the p53/p21 axis, thereby maintaining fibroblast proliferation and ECM synthesis capacity. Concurrently, PI3K/AKT can be hijacked by SASP to serve as a core signal amplifier driving pathological ECM remodeling. This dual regulatory role, contingent upon both context and signal intensity, renders targeting PI3K/AKT for intervening in fibrosis-associated cellular senescence a complex yet highly promising strategy.

### 3.5 PI3K/AKT signaling regulates the ECM by modulating autophagy in fibroblasts

Autophagy is a core process for maintaining intracellular homeostasis and plays a complex role in fibrotic diseases. This process mediates classical pathways, such as phagophore initiation, nucleation, autophagosome maturation, and recycling of contents, through the hierarchically ordered activity of ATG proteins. Additionally, non-canonical pathways exist, such as bypassing key factors like Beclin-1/ULK-1 and reshaping autophagosome membrane formation mechanisms, which synergistically eliminate dysfunctional cytoplasmic proteins and organelles (Codogno et al., 2011). In fibroblasts, the PI3K/AKT-mTOR signaling pathway serves as a central brake regulating autophagy activity. Its excessive activation strongly suppresses autophagy, leading to the accumulation of abnormal proteins and damaged organelles, and impeding ECM degradation, thereby driving the fibrosis process.

PI3K/AKT-mTOR serves as a pivotal hub linking ROS and autophagy regulation. Excessive intracellular ROS can activate PI3K/AKT, subsequently stimulating its downstream key effector mTORC1 (Ornatowski et al., 2020). Activated mTORC1 acts as a classical autophagy inhibitor, suppressing autophagy initiation by phosphorylating and inhibiting autophagy-initiating complexes such as ULK1. In keloid fibroblasts (KFs), inhibiting PI3K/AKT reduces mTORC1 activity, significantly enhancing mitochondrial autophagy, improving mitochondrial homeostasis, and downregulating fibrotic marker expression (Liu et al., 2024). Conversely, under other stress conditions, elevated mitochondrial ROS accompanied by activation of the PI3K/AKT/mTOR pathway may disrupt autophagy flux, promoting fibroblast senescence (Chen Q. et al., 2022). This indicates that the PI3K/AKT-mTOR axis serves as a critical interface for the OS-autophagy dialogue. In the context of fibrosis, the PI3K/AKT-mTOR pathway’s inhibition of autophagy is a key mechanism driving pathological ECM deposition. Extensive studies demonstrate that inhibiting PI3K/AKT enhances autophagic flux in fibroblasts, manifested by an increased LC3-II/I ratio (a key autophagy indicator), upregulation of Beclin-1 expression, and degradation of the autophagic substrate p62. This activation of autophagy is accompanied by a significant reduction in the expression of fibrosis markers such as COL1A1, FN, and α-SMA, thereby effectively alleviating fibrosis in multiple organs, including the lungs and heart (Li et al., 2023; Peng et al., 2023; He et al., 2020). Consequently, the PI3K/AKT-mTOR pathway provides fibroblasts with a survival advantage by suppressing autophagy, serving as a key mechanism for maintaining chronic matrix deposition (Haspel and Choi, 2011; Mizushima and Komatsu, 2011; Zhao et al., 2020b).

Furthermore, emerging research indicates that immune checkpoint molecules can directly hijack the PI3K/AKT-mTOR pathway, promoting fibrosis by inhibiting autophagy. Programmed death-ligand 1 (PD-L1) is upregulated in TGF-β1-induced LFs and enhances PI3K/AKT-mTOR signaling activation through positive feedback, suppressing autophagy and promoting fibroblast proliferation and migration. Treatment with PD-L1 antibodies reverses this process by uncoupling the PI3K/AKT-mTOR enhancement, thus restoring autophagic activity and alleviating pulmonary fibrosis (Lu et al., 2022). PD-1, a key immune checkpoint molecule in oncology, normally prevents immune cells from damaging self-tissues, thereby maintaining immune homeostasis. During tumorigenesis, its binding to the ligand PD-L1 suppresses T cell activity, hindering the immune system’s ability to recognize and eliminate tumor cells (Keir et al., 2008). More intriguingly, a silicosis study revealed a self-amplifying positive feedback loop between PI3K/AKT-mTOR and PD-L1 in fibroblasts, generating potent, sustained signaling that jointly drives fibroblast activation, proliferation, survival, and excessive ECM deposition. However, degradation of fibroblast PD-L1 via the ubiquitin-proteasome pathway may serve as a negative regulatory mechanism attempting to disrupt these positive feedback loops (Zhao S. et al., 2024). Under sustained pathological stimulation, this negative feedback often proves insufficient to counteract the intensely activated positive signals, ultimately leading to abnormal activation of the PI3K/AKT pathway—potentially a key mechanism in diseases like pulmonary fibrosis. This reveals profound cross-talk between immune signaling and the PI3K/AKT-mTOR-autophagy axis within the fibrotic microenvironment, providing theoretical support for therapeutic strategies combining immune modulation with autophagy intervention.

Within cellular autophagy, the PI3K/AKT-mTOR pathway serves as a critical negative regulatory hub. Its inhibition of autophagy directly leads to the accumulation of damaged organelles and abnormal proteins while impairing ECM turnover capacity. Therefore, inhibitors or natural products targeting this pathway not only restore autophagic homeostasis by blocking its core signaling but also effectively disrupt its self-amplifying vicious cycle, offering significant therapeutic potential for alleviating fibrosis.

### 3.6 PI3K/AKT signaling regulates the ECM by modulating glycolysis in fibroblasts

Beyond the aforementioned differential cellular responses to developmental signals, nutritional status, and DNA damage or ROS accumulation, glycolysis—a key pathway in cellular ATP energy metabolism—is a common metabolic process. At both cellular and molecular levels, glycolysis influences vital activities such as TGF-β1 activity, ECM synthesis, and fibroblast activation. He et al. discovered that during pulmonary fibrosis, lipopolysaccharide (LPS) activates the PI3K/AKT-mTOR pathway via Toll-like receptor 4 (TLR4). This process not only suppresses autophagy but also significantly enhances the proliferation, migration, and collagen secretion of mouse LFs(He et al., 2009; He et al., 2012; Xie et al., 2019). TLR4, a pattern recognition receptor (PRR) widely distributed in immune cells, epithelial cells, and fibroblasts, binds LPS to trigger a cascade of biological effects (Ciesielska et al., 2021). Notably, PI3K/AKT activation further upregulates PFKFB3 expression, a key glycolytic enzyme, suggesting aerobic glycolysis plays a crucial role in collagen synthesis by fibroblasts. PFKFB3 is a key enzyme involved in glucose metabolism, indicating that aerobic glycolysis plays an important role in collagen synthesis within LFs (Hu et al., 2020). Inhibiting the PI3K/AKT-PFKFB3 signaling cascade effectively blocks glycolysis and significantly reduces the expression of fibrosis markers such as FN and α-SMA, thus delaying pulmonary fibrosis progression (Li Y. et al., 2025).

In KFs, the interaction between HIF-1α and the PI3K/AKT pathway is equally prominent. It not only regulates the expression of glycolysis-related enzymes but also influences metabolic adaptation processes such as oxidative phosphorylation, although the specific mechanisms require further elucidation (Wang et al., 2021b). Furthermore, studies indicate that HIF-1α-overexpressing adipose-derived stem cell-derived exosomes (ADSCs-hEVs) significantly promote COL1/3 and VEGF expression in human fibroblasts by activating AKT phosphorylation (Wang L. et al., 2021), further establishing HIF-1α as a pivotal node linking metabolic reprogramming to ECM remodeling. However, other EVs components involved remain unidentified, necessitating further clarification of HIF-1α′s role.

In conclusion, the PI3K/AKT signaling pathway plays a crucial role in fibroblast activation, metabolic adaptation, and ECM deposition by regulating glycolysis. Elucidating the specific mechanisms by which this pathway coordinates energy metabolism and the fibrotic response not only aids in understanding the persistent progression of fibrotic diseases but also provides new directions for developing targeted therapeutic strategies that intervene in metabolic pathways.

### 3.7 PI3K/AKT signaling regulates the ECM by modulating ER stress in fibroblasts

Beyond OS, various intracellular and extracellular stressors can disrupt protein homeostasis within the ER, leading to ER stress. The ER is the cellular organelle responsible for synthesizing, folding, and maturing proteins. When misfolded proteins accumulate excessively within the ER, it triggers the unfolded protein response (UPR) (Chen and Cubillos-Ruiz, 2021). This conserved pathway is activated by three ER transmembrane proteins, inositol-requiring enzyme 1α (IRE1α), pancreatic endoplasmic reticulum kinase (PERK), and activator of transcription factor 6 (ATF6), to restore protein homeostasis. ER stress is implicated in the pathogenesis of numerous diseases (Oakes and Papa, 2015). During fibrosis progression, abnormal activation of the PI3K/AKT pathway has been demonstrated as a core upstream event driving ER stress in fibroblasts. Studies indicate that bleomycin (BLM) exposure initiates a sustained ER stress response in fibroblasts by activating the PI3K/AKT signaling axis, manifested by significant time-dependent increases in the levels of core UPR sensors such as PERK, ATF6, and IRE1α(Hsu et al., 2017). Crucially, pharmacological intervention with PI3K inhibitors effectively suppresses the induced expression of these downstream UPR effector molecules, directly confirming the dominant role of the PI3K/AKT pathway in upstream ER stress responses.

This PI3K/AKT-mediated ER stress is not an isolated event but directly promotes the fibrotic phenotype of fibroblasts. It not only drives excessive fibroblast proliferation but also synchronizes with pathological ECM remodeling, jointly accelerating fibrosis progression. As demonstrated by extracellular heat shock protein 90α (eHSP90α), activation of the PI3K/AKT pathway promotes ER stress and activation in IMR-90 pulmonary fibroblasts, manifested by markedly increased expression of α-SMA and COL1. More critically, ER stress within the fibrotic lung environment itself induces eHSP90α production, forming a positive feedback loop that continuously amplifies PI3K/AKT signaling and ER stress. This synergy sustains fibroblast activation and promotes chronic ECM deposition (Zhang et al., 2021).

Therefore, targeting this ER stress axis coordinated by PI3K/AKT offers a promising intervention strategy for interrupting the fibrotic process.

### 3.8 PI3K/AKT signaling regulates the ECM by modulating invadosomes in fibroblasts

During fibrosis, fibroblasts actively remodel the ECM not only through secretory functions but also by utilizing specialized mechanical adhesion structures known as invaginations. As subcellular structures that interact with the ECM and possess potent remodeling capabilities, invadosomes represent a cellular mechanism by which fibroblasts promote tissue infiltration and interstitial remodeling (Destaing et al., 2014). In IPF, a dynamic crosstalk exists between invadosomes activity and the matrix state: invadosomes modulate the ECM, while the physicochemical properties of the ECM in turn influence invadosomes formation and function (Juin et al., 2013).

Research indicates that activation of the PI3K/AKT pathway drives fibroblast formation of invadosomes. In LFs, abnormal activation of PDGFR downstream initiates the PI3K/AKT signaling axis, directly promoting invadosomes assembly (Lebel et al., 2022). This mechanism was validated in IPF patient tissues, where expression of the invaginome marker TKS5 (a key component of invadosomes) showed significant positive correlation with COL1 in IPF lung tissue and fibroblast foci. This suggests that invadosomes-mediated invasive growth and ECM deposition occur synergistically during fibrosis progression. Notably, the clinical antifibrotic drug nintedanib effectively blocks PI3K/AKT signaling by inhibiting PDGFR activity, thereby suppressing invadosomes formation and downregulating TKS5 expression. This provides a novel mechanistic perspective explaining part of its therapeutic efficacy in IPF.

To conclude, invadosomes research reveals an additional dimension of PI3K/AKT pathway regulation in fibroblast behavior. By conferring invasive matrix remodeling capabilities to fibroblasts, it drives the malignant progression of fibrosis. In-depth exploration of this pathway not only deepens our understanding of IPF pathogenesis but also provides crucial theoretical foundations for optimizing and developing novel anti-fibrotic therapeutic strategies.

## 4 Multidimensional regulatory network of PI3K/AKT signaling in fibroblasts regulating ECM

**FIGURE 4:**
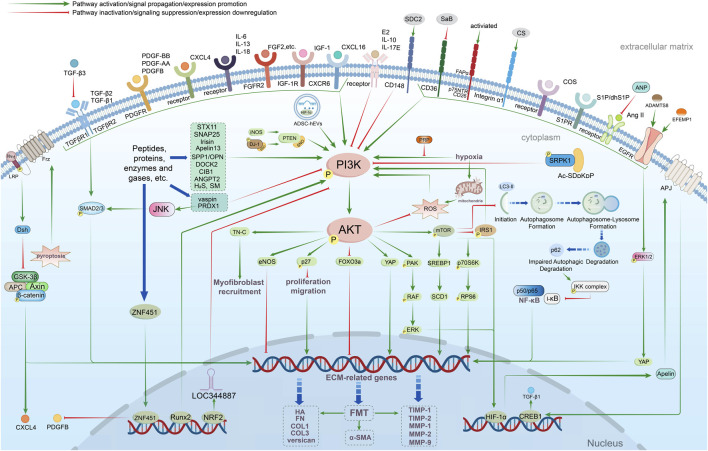
Multidimensional network regulation of PI3K/AKT-mediated ECM in fibroblasts of different origins. Fibroblasts are regulated by growth factors (TGF-β, IL-6, FGF2, IGF-1, and PDGF-AA/BB, among others), chemokines bound to surface targets (CXCL4/16), specific activation via other receptors on the surface (CD148, CD36, and p75NTR), and transcription factors (Runx2, NRF2, and HIF-1), a variety of other peptides and proteins, exogenous H_2_S gas, cell entry induction by ADSCs-hEVs, hypoxia-induced, cellular pyrolysis-mediated PI3K/AKT-related downstream cascades mediate fibroblast life activities including value-added migration, apoptosis, autophagy, senescence, ECM secretion and FMT. TGF-β, Transforming growth factor-β; TGFβR, Transforming growth factor bata receptors; CXCL16, C-X-C chemokine ligand 16; CXCR6, C-X-C chemokine receptor 6; IL-6, Interleukin-6; FGF2, Fibroblast growth factor 2; IGF-1, Insulin-like growth factor-1; PDGF-BB, Platelet-derived growth factor-BB; PDGF-AA, Platelet-derived growth factor-AA; SDC2, Syndecan-2; CD148, Cluster of differentiation 148; CD36, Cluster of differentiation 36; p75NTR, p75 neurotrophin receptor; CS, Chondroitin sulfate; S1P/dhS1P, Sphingosine-1-Phosphate/dihydro-Sphingosine-1-Phosphate; Runx2, Runt-related transcription factor 2; FMT, Fibroblast-to-myofibroblast transition; HIF-1, Hypoxia-inducible factor-1; Bcl-2, B-cell lymphoma-2; PRP, Platelet-rich plasma; SREBP1, Sterol regulatory element binding protein 1; sMAF, Small musculoaponeurotic fibrosarcoma; HA, hyaluronic acid; FN, Fibronectin; Ac-SD_D_K_D_P, N-acetyl-seryl-aspartyl-lysyl-proline with Asp and Lys residues substituted with D-amino acids; CIB1, Calcium and integrin binding protein 1; ADAMTS8, A disintegrin and metalloproteinase with thrombospondin motifs 8; FAPα, fibroblast activation protein-α; EFEMP1, EGF-containing fibulin extracellular matrix protein 1; iNOS, inducible nitric oxide synthase; DJ-1, PARK 7 gene encodes a protein of 189 amino acids; Vaspin, An inhibitor of visceral adipose tissue-derived serine protease; ZNF451, Zinc finger protein 451; Ang II, Angiotensin II; COS, Chitooligosaccharide; OPN, Osteoblast protein; PRDX1, Peroxiredoxin-1; DOCK2, Dedicator of cytokinesis 2.

The preceding section delineates the pathophysiological alterations in fibroblasts mediated by the PI3K/AKT pathway. Subsequently, we will investigate how diverse fibroblast subtypes modulate ECM modifications via multiple signaling pathways and targets, thereby constructing a multidimensional signaling framework (Figure 4).

### 4.1 Cytokines

#### 4.1.1 TGF-β

The PI3K/AKT signaling pathway serves as a central regulator in tissue microenvironment remodeling by integrating multiple upstream stimuli. Among these, growth factors play a pivotal mediating role in PI3K/AKT signaling activation.

TGF-β is a major cytokine regulating fibrosis primarily through the classical SMAD signaling pathway. Additionally, TGF-β can utilize non-classical pathways, such as the PI3K/AKT pathway, to regulate collagen balance in the ECM(Guo et al., 2021). Upon TGF-β stimulation, fibroblasts upregulate type I collagen (COL1A1 and COL1A2), type III collagen (COL3A1), and type V collagen (COL5A1 and COL5A2) (Merl-Pham et al., 2019). TGF-β1 regulates the PI3K/AKT pathway and induces fibrosis and COL1 expression (Mizushima and Komatsu, 2011). In the respiratory system, TGF-β1 activates stearoyl-CoA desaturase 1 (SCD1) expression in human fetal lung fibroblast-1 (HFL-1) via the PI3K/AKT-mTOR-SREBP1 pathway, promoting cellular activation and airway remodeling (Zhou et al., 2023). SREBP1, or sterol regulatory element-binding protein 1, is a key transcription factor regulating lipid metabolism and can be activated by mTOR (Ma et al., 2021) to enhance SCD1 function in airway cells. SCD1 has been extensively studied in lung cancer pathogenesis and plays a crucial role in cell proliferation and metastasis (Noto et al., 2017). Recent studies summarizing SCD1 research in CAFs indicate that SCD1-mediated lipid metabolic reprogramming drives sustained secretion of lipids and cytokines by CAFs. This regulates ECM remodeling and establishes an immunosuppressive microenvironment, indirectly promoting tumor progression (Mohammad et al., 2025). Consequently, SCD1 serves as a crucial effector molecule in maintaining ECM homeostasis and acts as a key bridge linking PI3K/AKT signaling to microenvironmental remodeling.

TGF-β1 further activates dual pathways in LFs: PI3K/AKT-HIF-1α and PI3K/PAK/RAF/ERK/HIF-1α. This dual activation promotes FMT and SDF-1 secretion. Crucially, it was first demonstrated that SDF-1 directly induces TGF-β secretion in M2 macrophages via these pathways. ERK/HIF-1α dual pathways in LFs promote FMT and SDF-1 secretion. Notably, SDF-1 directly induces TGF-β secretion in M2 macrophages via these pathways, forming a positive feedback loop that offers novel therapeutic targets for pulmonary fibrosis (Fu et al., 2023). In the classical pathway, both PI3K and RAF can be activated by the key intermediate molecule RAS GTPase, which in turn activates the AKT and MEK/ERK cascades, respectively (Li Q. et al., 2022). In contrast, in the non-classical pathway, PI3K regulates the P-Rex1-dependent activation of Rac1, thus activating the PAK/RAF/MEK/ERK pathway and triggering subsequent cellular responses (Ebi et al., 2013).

Additionally, the PI3K/AKT pathway activated by TGF-β1 in endometrial fibroblasts (EFs) may directly mediate progressive adenomyosis (AM) (Niu et al. 2023). In ocular fibrosis, TGF-β2 binding to its receptor was found to activate multiple signaling pathways, including PI3K/AKT, in human conjunctival fibroblasts (HConFs), leading to cross-talk and influencing ECM alterations (Liu et al., 2020). Similar mechanisms have been explored for additional therapeutic approaches targeting TGF-β2 in HConFs, though these do not involve PI3K/AKT signaling (Umetsu et al. 2024; Oouchi et al. 2021). Unlike the first two TGF-β isoforms, TGF-β3 may exert distinctly different effects and even inhibit their actions. For example, Murata et al. demonstrated that TGF-β3 promotes COL1/3 synthesis in HDFs through both TGF-β1-dependent and -independent mechanisms (Murata et al. 1997). However, evidence linking TGF-β3 to PI3K/AKT signaling remains limited and warrants further investigation (Bettahi et al. 2014).

In summary, the mechanisms of TGF-β regulation are complex and implicated in many diseases, including fibrosis and cancer. TGF-β can regulate changes in EMT, ECM secretion, cellular immune invasion, and activation of CAFs as a cancer-promoting factor (Peng et al., 2022). Theoretically, TGF-βR inhibitors can block the PI3K/AKT pathway in fibroblasts. However, given the ubiquitous nature of TGF-βR, targeting may shift to its downstream effectors, combined with other therapies to enhance efficacy and reduce side effects. Comprehensive lists of relevant targeted therapeutics are available in recent reviews (Deng et al., 2024).

#### 4.1.2 IGF-1

Insulin-like growth factor-1 (IGF-1) is a small-molecule hormone synthesized by the liver under the regulation of growth hormone (GH) (Singh et al., 2015). Fibroblasts serve as key producers and responders of IGF-1 in skin and various tissues. Through a closed-loop system involving synthesis, secretion, signaling, and functional feedback, they jointly regulate tissue homeostasis, repair, and aging (Singh et al., 2015; Böhm et al., 2025). IGF-1 release is significantly reduced in aged fibroblasts *in vitro*, as young fibroblasts exhibit markedly higher IGF-1 synthesis capacity than aged fibroblasts. When fibroblasts enter a senescent state due to ROS accumulation, DNA damage, etc., the core transcription factor JunB of the activator protein-1 (AP-1) family is activated. This upregulates the expression of the cell cycle inhibitor p16^INK4A^. This not only induces fibroblast senescence but also directly suppresses IGF-1 gene promoter activity, leading to a substantial decline in IGF-1 synthesis. This process manifests in skin aging as a progressive loss of fibroblast-derived IGF-1 (Maity et al., 2021).

The reduction in IGF-1 also inhibits the synthesis of collagen and various other structural proteins in fibroblasts, leading to ECM imbalance in tissues (Noordam et al., 2013; Yin et al., 2020). The regulatory role of IGF-1 is closely associated with the PI3K/AKT cascade (Povsic, Kohout, and Lefkowitz, 2003; Disser et al., 2019). For instance, during cardiac fibrosis, IGF-1 binding to its receptor activates the PI3K/AKT-FOXO3a signaling cascade to remodel cellular homeostasis, contributing to the onset and progression of atrial fibrillation (Zhang P. et al., 2023). Under normal conditions, the transcription factor FOXO3a downregulates COL1 expression in fibroblasts. Pathway activation induces FOXO3a phosphorylation and inactivation, thereby releasing its inhibitory effect on ECM synthesis (Zhang Q. et al., 2023). In keloids, IGF-1 induces activation of the PI3K/AKT-mTOR signaling pathway, upregulating CD26 expression on KF surfaces and enhancing cell proliferation and invasion (Xin et al., 2020). CD26, a type II transmembrane protein closely associated with KF biological activities, may serve as a functional marker for fibroblasts (Rinkevich et al., 2015). Furthermore, studies on orbital fibroblasts (OFs) indicate that the specific IGF-1R inhibitor Linsitinib effectively suppresses proliferation and HA secretion through a mechanism involving concurrent attenuation of PI3K/AKT and ERK signaling pathways, providing novel pharmacological rationale for targeted therapy of thyroid-associated ophthalmopathy (TAO, also known as GO) (Lee et al., 2024).

Therefore, targeting the regulation of fibroblast-IGF-1 interactions has become a critical research direction for fibrotic and TAO-related diseases. In-depth analysis of PI3K/AKT and related signaling pathways may provide key targets for tissue repair and anti-aging interventions.

#### 4.1.3 PDGF

The PDGF family comprises five isoforms that form homo- or heterodimers via disulfide bonds: PDGF-A (forming AA homodimers), PDGF-B (forming BB homodimers or AB heterodimers with PDGF-A), PDGF-C (forming CC homodimers), PDGF-D (forming DD homodimers) (Fredriksson et al., 2004). PDGF is a potent mitogen for fibroblasts, myofibroblasts, and other MSCs (Clark et al., 1993), inducing fibroblast proliferation, migration, and differentiation while regulating the balance between ECM synthesis and degradation (Bonner, 2004). The mechanism involves PDGF binding to the tyrosine kinase receptors PDGFR-α/β on the cell surface, leading to receptor dimerization. This triggers cross-linking and phosphorylation of the receptors in the cytoplasmic domain, subsequently activating cellular responses through the PDGF signaling pathway. This pathway comprises components such as PI3K, phospholipase Cγ, Janus kinase (JAK)/signal transducer and activator of transcription (STAT), and RAS pathways (Irma et al., 2025). Regarding PDGF subtypes, their actions also differ. James C Bonner’s review summarizes that PDGF-AA primarily acts through PDGFRα, exists as an autocrine loop in pulmonary fibrosis and scleroderma, exhibits weak chemotactic activity, and has a mild proliferative effect. In contrast, PDGF-BB binds to PDGFRα/β heterodimers, exhibiting the strongest proliferative and chemotactic activities. It is the core subtype in liver and kidney fibrosis, capable of inducing the expression of multiple fibrosis-related genes. Newer subtypes include PDGF-C, which drives fibrosis via PDGFRα, and PDGF-D, which specifically binds PDGFRβ and serves as a novel mediator in mesangial proliferative nephritis (Bonner, 2004).

In anti-skin aging research, PDGF-AA activates the PI3K/AKT signaling pathway in HDFs, significantly increasing COL1/3, elastin, and TIMP-1 expression while decreasing MMP-1 and MMP-9 expression (Guo et al., 2020). In this study, PDGF-AA was delivered via stem cell-conditioned medium, potentially obscuring other complex molecular interactions. Similarly, in cardiac fibrosis, PDGF-AA significantly induces CFs proliferation and ECM protein synthesis (Simm et al., 1998). Furthermore, in pulmonary fibrosis, upregulation of PDGF-B promotes COL1A1, COL3A1, and FN1 expression by activating the PI3K/AKT pathway, closely linked to pulmonary fibrosis pathogenesis (Peng et al., 2024; Aono et al., 2014; Noskovičová et al., 2015). In the oral cavity, PDGF-BB binding to PDGFR-β triggers PI3K catalytic activity in oral mucosal fibroblasts, initiating a PDK1-mediated AKT phosphorylation cascade that promotes fibroblast-to-myofibroblast transition, collagen synthesis, and cell migration in oral submucosal fibrosis (Wang Q. et al., 2021). PDGF is also overexpressed in infarcted myocardium, where it likely regulates fibroblast function (Zymek et al., 2006). As previously noted, PI3K/AKT and CaMKII interact in HLFs to coordinately regulate apoptosis (Zhao et al., 2020a). Therefore, the therapeutic benefits and drawbacks of PDGF-modulating agents warrant further investigation, and the development of CaMKII inhibitors represents a promising therapeutic avenue for cardiac and pulmonary diseases (McMullen et al., 2020).

Upstream signaling represented by PDGF activates the PI3K/AKT pathway, playing a central regulatory role in multiple processes, including skin repair, organ fibrosis, and pathological scar formation. This mechanism positions the PI3K/AKT pathway as a highly promising integrated therapeutic target (Bonner, 2004).

#### 4.1.4 Chemokines

Chemokines are small, multifunctional signaling proteins that exhibit diverse effects in various cell types, are significantly activated during inflammatory responses, and mediate biological functions such as cell proliferation, angiogenesis, and remodeling (Mempel et al., 2024). Fibroblasts both secrete and respond to chemokines, establishing autocrine and paracrine signaling loops. During fibrosis, chemokines function as potent chemotactic agents, recruiting key effector cells such as myofibroblasts to sites of tissue injury, where they act in concert with pro-fibrotic cytokines to drive disease progression (Wynn, 2008). Within the chemokine-mediated fibrosis mechanism, the CC- and CXC-chemokine receptor families consistently exhibit crucial regulatory roles. For instance, CXC chemokine receptor 4 (CXCR4) and CC chemokine receptor 2 (CCR2) have been demonstrated to regulate the recruitment of mouse fibroblasts to the lungs (Wynn, 2008). Anti-CCL3 antibodies significantly inhibit pulmonary fibrosis progression (Smith et al., 1995). Furthermore, knockout of CXCL16 inhibits renal fibroblast aggregation and myofibroblast formation while reducing ECM production (Xia et al., 2013). Conversely, CXCL16 promotes CFs proliferation and collagen secretion (Wang H. et al., 2014). In recent years, CXCL16 has been identified as a pro-fibrotic factor, existing in both transmembrane and soluble forms as a novel phosphatidylserine and oxidized LDL cell surface clearance receptor (Xia et al., 2013). Mechanistic studies reveal that binding to its receptor CXCR6 leads to phosphorylation and activation of the PI3K/AKT-FOXO3a cascade, thereby promoting fibroblast proliferation and collagen production (Ma et al., 2020). Regarding advances in CXCL16/CXCR6 research in fibrosis, Wang et al. (Wang J. et al., 2024) provided a comprehensive summary. We observed that appears to play a crucial regulatory role in PI3K signaling primarily within fibroblasts of renal (Xia et al., 2014; Xia et al., 2013) and pulmonary (Ma et al., 2020) tissues.

Additionally, CXCL4 has been found to mediate collagen secretion via the PI3K/AKT pathway (Wei et al., 2024). Interestingly, this study revealed that pyroptosis induces CXCL4 secretion in mouse cardiac myofibroblasts (MCFs) by activating the Wnt/β-Catenin pathway, which then feedback activates the cellular PI3K/AKT signaling pathway to promote cardiac fibrosis. Pyroptosis is a programmed necrotic cell death process mediated by gasdermin. During pyroptosis, inflammasomes activate gasdermin and caspases, leading to the release of inflammatory cytokines such as CXCL4 (Wong, 2011; Newton et al., 2024). However, the specific CXCL4 receptors on cardiac myofibroblasts remain undefined, and the roles of proposed receptors such as TLRs (Yang C. et al., 2022) and CXCR3 (Deng et al., 2019) are not fully elucidated presenting a significant challenge for therapeutic targeting of CXCL4.

Collectively, research on the chemokine-PI3K/AKT-ECM axis in fibroblasts remains nascent, representing an area of substantial potential and significant challenge.

#### 4.1.5 Interleukin

Within the interleukin family, beyond promoting inflammatory progression, numerous interleukin types are associated with fibroblast-ECM interactions. Lurje et al. highlighted that fibroblast-interleukin interactions constitute a critical regulatory link in the pathological process of fibrosis (Lurje et al., 2023). This involves IL-1β stimulating MMP3 secretion (Ainola et al., 2005), IL-4 and IL-13 activating STAT-6 to regulate transcription of genes related to monocyte-to-fibroblast differentiation via nuclear translocation (Shao et al., 2008), TNF and IL-17A synergistically activating the specific molecule IκBζ to amplify the fibrotic response (Slowikowski et al., 2020), and IL-11 mediating ERK signaling to drive fibroblast activation and collagen synthesis, leading to ECM deposition or tissue remodeling (Schafer et al., 2017). Furthermore, IL-25 (IL-17E) promotes TGF-β1 availability both directly, via autocrine secretion in fibroblasts, and indirectly, by inducing M2 macrophages. This bioavailable TGF-β1 then activates the SMAD2/3-SMAD4 pathway, enhancing collagen expression and facilitating organized ECM deposition. In diabetic wound models, this IL-25-driven mechanism improves collagen alignment and supports angiogenesis, promoting repair (Li S. et al., 2022).

At the mechanistic level, multiple interleukins promote ECM metabolism through the PI3K/AKT signaling pathway. IL-18 activates PI3K via its receptor and the downstream MyD88/IRAK1/TRAF6 complex, subsequently promoting the expression of ECM components such as FN through the AKT–IKKβ–NF-κB pathway, thus participating in myocardial fibrosis (Reddy et al., 2008). Similarly, IL-13 binding to the IL-13Rα2 receptor activates PI3K/AKT, which acts synergistically with PKC signaling to upregulate collagen and fibronectin synthesis, driving fibrosis in oral mucosa and skin (Wang, H. et al., 2024; Jinnin et al., 2006). In addition, IL-6 binding to its receptor activates PI3K/AKT signaling in synovial fibroblasts (SFLs), inducing their transformation into myofibroblasts. This process is accompanied by upregulation of ECM-related proteins such as α-SMA and COL1, ultimately leading to fibrosis (Yang BY et al., 2022). Concomitantly, PI3K/AKT activation enhances fibroblast migration via AKT phosphorylation, further contributing to tissue remodeling (Nishikai-Yan Shen et al., 2017).

On the other hand, certain interleukins exhibit anti-fibrotic potential. For instance, IL-10 activates the PI3K/AKT pathway while simultaneously stimulating STAT3 signaling, establishing cross-talk between these pathways. This inhibits the expression of fibrosis-related proteins such as COL1/3 and α-SMA, reducing ECM deposition and hyperplastic scar (HS) formation (Shi et al., 2014). This effect, which contrasts with the common pro-fibrotic outcome of PI3K/AKT activation, may be attributed to the specific context of IL-10 signaling and its concurrent activation of the STAT3 pathway.

Notably, the functional roles of interleukins in fibrosis are highly diverse and tissue-specific. For example, IL-17 promotes neutrophil infiltration and synergizes with TGF-β1 and IL-1β to exacerbate ECM deposition in IPF, yet exhibits dual pro- and anti-fibrotic functions in dermal and renal fibrosis, as well as in atherosclerosis (Ramani and Biswas, 2019). Similarly, IL-4 exhibits duality in inflammation, capable of both promoting and resolving inflammatory responses to facilitate tissue repair (Pan et al., 2025; Zeng et al., 2013). It is also implicated in regulating metabolism (Run et al., 2023) and neural regeneration (Pan et al., 2022). It is noteworthy that IL-4 and IL-13 share structural and functional similarities and are often studied in tandem (Bernstein et al., 2023).

These cytokines, with their dual pro-/anti-inflammatory and pro-/anti-fibrotic functions, contribute to a complex compensatory network. Significant patient subtype and organ heterogeneity further complicate the disease landscape. These findings underscore the multifaceted nature of interleukin signaling in fibrosis. Consequently, targeting the immune-fibroblast-PI3K/AKT (and STAT3, TGF-β/SMAD, etc.)-ECM axis presents considerable therapeutic promise, albeit with substantial challenges.

### 4.2 ECM active feedback control

The ECM itself is not merely a passive product; it can also actively signal (via matrix components, stiffness, etc.) to reciprocally regulate fibroblasts. For example, chondroitin sulfate (CS), an ECM component, and integrin α1 can promote fibroblast proliferation and migration by activating the PI3K/AKT pathway, thus influencing tissue repair (Katayama et al., 2020). FN-1, another ECM component secreted by fibroblasts, is extensively involved in pathophysiological changes within tissues (Liu et al., 2019) and is closely associated with PI3K/AKT signaling (Yang J. et al., 2022). Furthermore, ECM stiffness-induced stress is a key driver of myofibroblast activation (Tschumperlin et al., 2018; Lagares and Hinz, 2021), as the complex interplay between mechanosignals triggered by ECM stiffness and fibroblast responses is elaborated in this section (Hinz and Lagares, 2020).

When cells exert tensile or compressive forces on the ECM during various biological processes, the ECM first generates a resistive effect, the strength of which is determined by its inherent stiffness (Saraswathibhatla et al., 2023). Taking skin as an example, healthy skin can be regulated and maintained by an intact ECM when subjected to stretching, compression, twisting, and interstitial fluid flow. When the dermal ECM is damaged, fibroblasts are exposed to mechanical stress, leading to myofibroblast activation and subsequent matrix remodeling (Sawant et al., 2021). This closely correlates with the stiff, fibrotic skin observed in HS and scleroderma lesions, as well as the myofibroblast-rich granulation tissue formed in response to injury (Ehrlich et al., 1994; Sappino et al., 1990). Stiffness is typically characterized by the elastic modulus. In the human body, stiffness values range from hundreds to thousands of pascals in soft tissues, reaching tens of thousands of pascals in muscle tissue (Levental et al., 2007; Saraswathibhatla et al., 2023).

In fibrotic environments, excessive collagen deposition and increased myofibroblast contraction lead to ECM that is stiffer than that in healthy organs (Hinz and Lagares, 2020). This ECM stiffening further promotes TGF-β1 release and biomechanical activation of myofibroblasts, forming a vicious positive feedback loop that drives fibrosis progression (Hinz et al., 2019; Hinz, 2009). Recent mechanobiology research has focused on elucidating how ECM stiffness modulates fibroblasts. For instance, fibroblasts perceive and respond to mechanical cues from the external environment through diverse cell surface receptors, including integrins (αvβ1, αvβ3, etc.), discoidin domain receptors, GPCRs, and stretch-activated ion channels (Saraswathibhatla et al., 2023).

Although the association between ECM stiffness and the PI3K/AKT pathway has been recognized, its upstream mechanisms remain unclear. Existing studies indicate that multiple mechanosensitive cell surface proteins converge on downstream PI3K signaling. Among these, focal adhesions serve as sensors for various mechanical cues and function as primary connection points between cells and their matrix. Numerous proteins within the focal adhesion machinery, including integrins (Katayama et al., 2020) and focal adhesion kinase (FAK) (Geletu et al., 2022), influence PI3K activation. Protein tyrosine kinases (PTKs), common upstream receptors for PI3K activation, may modulate PI3K/AKT phosphorylation through their regulation of focal adhesion formation (Schlessinger, 2000). One study demonstrated that valve interstitial cells (VICs) perceive matrix stiffness signals through mechanosensitive proteins in the cell membrane, activating the PI3K/AKT pathway. This promotes αSMA expression and stress fiber formation, driving their differentiation into myofibroblasts and inducing nodule formation (Wang et al., 2013). However, this study did not delve into how upstream mechanosensitive molecules (e.g., integrins, mechanosensitive ion channels) activate this pathway.

Recent research in mouse dermal fibroblasts (MDFs) revealed that fibroblast activation protein-alpha (FAPα) functions as a mediator of mechanical signal transduction. The specific mechanism involves increased ECM deposition during skin fibrosis, which elevates perceived matrix stiffness and subsequently upregulates FAPα expression. This then triggers PI3K-mediated fibrotic effects, exacerbating skin fibrosis. Notably, the interaction mechanisms between FAPα and mechanosensitive proteins like integrins and FAK remain unclear in this study (He et al., 2024).

To summarize while existing studies have clarified the role of the PI3K/AKT pathway in fibroblast perception of ECM stiffness and phenotypic regulation, precise elucidation of upstream molecular interactions, including but not limited to those involving focal adhesion components and FAPα, remains lacking. Furthermore, challenges regarding tissue specificity and dose dependency in targeted interventions have not been fully resolved, limiting clinical translation efficiency. Future efforts should focus on elucidating tissue-specific molecular nodes within the ECM stiffness-PI3K/AKT regulatory network. This could enable the development of precision intervention strategies targeting the focal adhesion-FAPα-PI3K/AKT axis. Integrating these approaches with dynamic force-responsive biomaterials could provide more targeted therapeutic solutions for fibrotic diseases while offering theoretical support for the biomimetic construction of cellular microenvironments in tissue engineering.

### 4.3 ncRNA regulation

**FIGURE 5:**
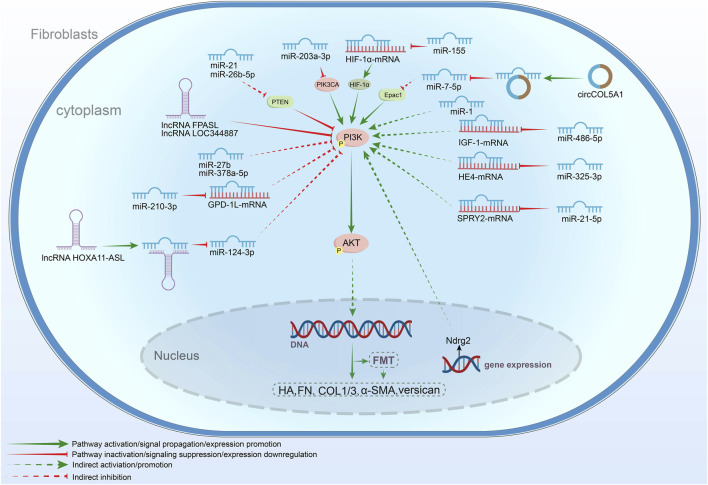
ncRNA regulation of ECM by PI3K/AKT in fibroblasts of different origins. miR-486-5p, miR-325-3p, miR-21-5p, miR-203a-3p and miR-155 inhibit PI3K/AKT signaling by suppressing IGF-1, HE4, SPRY2, PIK3CA and HIF-1α, respectively; miR-21 and miR-26b-5p promote PI3K/AKT signaling by inhibiting PTEN/AKT signaling; miR-27b and miR-378a-5p may directly inhibit PI3K/AKT signaling indirectly by binding to the corresponding mRNAs; miR-210-3p inhibits GPD-1L synthesis to promote the PI3K/AKT cascade; miR-1 may directly or indirectly promote PI3K/AKT; circRNACOL5A1 binds to miR-7-5p binding inhibits Epac1 synthesis to promote PI3K/AKT activation; lncRNA HOXA11-ASL indirectly promotes PI3K/AKT signaling cascade by inhibiting its action through binding to miR-124-3p; lncRNA FPASL/LOC344887 may directly inhibit downstream ECM induced by PI3K/AKT signaling cascade expression and FMT. FMT, Fibroblast-to-myofibroblast transition; GPD-1L, glycerol-3-phosphate dehydrogenase 1-like; HE4, Human epididymis protein 4; Ndrg2, N-myc downstream-regulated gene 2; COL1/3, Collagen type I and collagen type III; HA, hyaluronic acid; FN, Fibronectin.

Genetic material within fibroblasts also plays a pivotal role in mediating PI3K/AKT signaling. In recent years, the impact of both genes and non-coding RNAs (ncRNAs) on this pathway has been extensively explored. For example, N-myc Downstream Regulatory Gene 2 (NDRG2), a tumor suppressor gene within the NDRG family, has been shown to positively regulate PI3K/AKT signaling in DFs. This regulation enhances cell proliferation and migration, thereby accelerating the progression of skin fibrosis (Yu et al., 2025). In this section, we summarize the regulation of PI3K/AKT signaling in fibroblasts by ncRNAs (Figure 5).

ncRNAs include microRNAs (miRNAs), long non-coding RNAs (lncRNAs), and circular RNAs (circRNAs). Among these three types of ncRNAs, both lncRNAs and circRNAs can act as miRNA “sponges,” while miRNAs can also regulate the degradation and transcriptional activity of the other two types of RNA. Although they differ in structure and function, they form a complex gene regulatory network through mutual regulation. Many types of these molecules have been identified as key regulators of the PI3K/AKT signaling pathway in fibroblast-mediated fibrosis. By targeting upstream or downstream factors within the pathway or participating in signaling network regulation, they play a central role in modulating fibroblast function and ECM remodeling. miRNA regulation exhibits organ- and cell-specificity. For instance, in renal fibrosis, miR-378a-5p directly inhibits the PI3K/AKT pathway cascade to alleviate fibrosis in mouse NRK-49F cells (Zhang P. et al., 2023). In cardiac fibrosis, miR-21 promotes CFs proliferation and ECM deposition by activating the pathway through PTEN targeting, while liraglutide reverses this effect by downregulating AngII-induced miR-21 expression (Wang J. et al., 2023). miR-210-3p activates pathways by targeting Glycerol-3-phosphate dehydrogenase 1-like (GPD-1L) to drive atrial fibrosis (Hao et al., 2022). Strikingly, miR-486-5p exhibits opposing functions: it activates PI3K/AKT by targeting IGF1 inhibitors to drive proliferation in human dermal fibroblasts (Xiao, 2021), yet inactivates the pathway and exacerbates fibrosis in cardiac fibroblasts, underscoring profound fibroblast heterogeneity (Zhang X. et al., 2025). In pulmonary vascular disease PAH and ventricular fibrosis, miR-1 plays a pro-fibrotic role by activating PI3K/AKT (Liu Y. et al., 2021). Conversely, miR-325-3p suppresses the pathway and the expression of COL1/3 and MMP-2/9 by targeting human epididymis protein 4 (HE4) (Tang et al., 2022). In skin fibrosis, downregulation of miR-27b or exosomal delivery of miR-26b-5p (which targets PTEN) activates PI3K/AKT to promote ECM synthesis (Sun K. et al., 2020; Dai et al., 2024), while miR-155 (Wu et al., 2018)、miR-203a-3p (Zhao et al., 2025; Zhao Y. et al., 2024)、miR-21-5p (Wu et al., 2020) exert anti-fibrotic effects and promote wound healing by regulating HIF-1α, PIK3CA, and SPRY2, respectively.

In contrast to the relatively well-defined regulatory patterns of miRNAs, the mechanisms by which lncRNAs regulate the PI3K/AKT pathway are less characterized and require further elucidation. Nonetheless, specific lncRNAs such as FPASL and LOC344887 have been shown to negatively regulate PI3K/AKT, inhibiting hypertrophic scar fibroblasts (HSFs) proliferation and modulating ECM in IPF, respectively (Liu G. et al., 2021; Ma et al., 2022). Many studies remain at the bioinformatics prediction stage. For instance, the FGF2 signaling axis may promote DFs migration and ECM deposition by regulating lncRNAs such as HOXA-AS2 and H19 to mediate PI3K/AKT signaling (Wu et al., 2021). Similarly, bioinformatic analyses suggest that cardiac fibrosis-associated lncRNAs (e.g., NONHSAG005537, NONHSAG017620) may influence ECM remodeling by co-regulating multiple pathways, including focal adhesion, Hippo, PI3K/AKT, and TGF-β signaling (Han et al., 2023).

circRNA distinguished by their circular configuration and robust stability, possess significant regulatory capabilities. For example, in keloids, circCOL5A1 mitigates the inhibitory effect of miR-7-5p on Epac1 through binding, subsequently triggering the PI3K/AKT-ECM protein cascade in HKFs and suppressing apoptosis (Lv et al., 2021a). Examination of circRNA ceRNA networks in myocardial fibrosis further elucidates that differentially expressed circRNAs modulate CFs proliferation and differentiation by regulating pathways such as PI3K/AKT and TGF-β, underscoring their essential role in the progression of cardiac fibrosis (Gu et al., 2020).

In summary, the regulatory mechanisms of miRNAs are the most extensively characterized and display pronounced cell- and organ-specificity. By modulating the PI3K/AKT pathway, miRNAs directly govern fibroblast proliferation, migration, and extracellular matrix synthesis. While lncRNAs have been demonstrated to affect fibroblast function and extracellular matrix deposition through involvement in signaling networks regulating the PI3K/AKT pathway, the precise regulatory networks warrant additional elucidation. CircRNAs, capitalizing on their inherent stability, modulate the PI3K/AKT-extracellular matrix cascade via mechanisms like ceRNA, providing novel insights into fibrosis mechanisms. Collectively, these three classes of ncRNAs contribute to the initiation and progression of fibrosis by intricately and differentially regulating the PI3K/AKT pathway. This lays a vital groundwork for comprehending the pathological mechanisms of fibrosis and devising targeted intervention strategies. However, the synergistic or antagonistic interactions among different non-coding RNAs and their tissue-specific regulatory patterns necessitate further investigation.

### 4.4 Cell membrane receptor-mediated

Beyond the ligands described above, many studies have explored the role of cell membrane receptors. In addition to the classic receptor tyrosine kinases (e.g., PDGFR), G protein-coupled receptors (e.g., CXCR6), and serine/threonine kinase receptors (e.g., TGF-βR) previously mentioned, research has identified numerous other critical receptors on fibroblast surfaces. These receptors significantly impact ECM homeostasis by modulating the PI3K/AKT signaling pathway. Given their functional diversity, these receptors represent a promising target library for precise fibrosis intervention. Activation of specific receptors amplifies signaling in the PI3K/AKT pathway, thereby promoting fibrosis. For instance, in frozen shoulder-associated fibrosis, the class B scavenger receptor CD36 has been demonstrated to augment PIP3 biosynthesis, facilitate AKT membrane translocation and phosphorylation, and consequently drive excessive ECM deposition (Yan et al., 2023). Similarly, in scar formation, silencing the p75 neurotrophin receptor (p75NTR) effectively inhibits abnormal proliferation, migration, and ECM secretion of HSFs by suppressing PI3K/AKT-mTOR signaling and activating autophagy, highlighting its role as a mediator of pro-fibrotic signaling (Shi N. et al., 2021).

Notably, a study identified fibroblast growth factor receptor 2 (FGFR2) as an upstream activator of the PI3K/AKT pathway and a key target in the PDGFRA + fibroblast subtype. During the cystic stage, FGFR2 signaling promotes PDGFRA^+^ fibroblast differentiation into myofibroblasts via the PI3K/AKT pathway while inhibiting differentiation into stromal fibroblasts and adipofibroblasts. Conversely, FGFR2 signaling deficiency reverses this effect, resulting in increased alveolar type 2 (AT2) cell self-renewal and decreased AT1 cell differentiation, leading to abnormal postnatal alveolarization and alveolar simplification. This finding suggests that an imbalance in fibroblast subtypes drives fibrosis (Riccetti et al., 2024).

Conversely, some receptors negatively regulate the PI3K/AKT pathway, and their activation can mitigate the fibrotic phenotype. In IPF models, the transmembrane phosphatase CD148 has been shown to inhibit PI3K/AKT signaling by preventing p62 protein aggregation and restoring autophagy function, thereby suppressing the transcription of ECM-associated genes (Tsoyi et al., 2021). Although the exact mechanism by which it inhibits NF-κB signaling is not fully understood, its activator SDC2 has shown promising effects in animal models, offering a potential direction for drug development. More intriguingly, as illustrated by the G protein-coupled estrogen receptor (GPER), the same receptor can exhibit complex regulatory patterns depending on the context. In models of shoulder joint fibrosis and myocardial fibrosis, GPER activation exerts anti-fibrotic effects by inhibiting the PI3K/AKT axis. Its agonist G-1 and estrogen significantly reduce ECM deposition and improve fibrotic phenotypes (Prossnitz and Barton, 2023; Wang Z. et al., 2025; Alencar et al., 2017; Tofovic et al., 2009).

To summarize, receptors with both positive and negative regulatory roles, including CD36, p75NTR, FGFR2, CD148, and GPER, collectively form a sophisticated membrane signaling perception and transduction network. These receptors integrate diverse extracellular and intercellular signals into the PI3K/AKT pathway, ultimately determining ECM fate through modulation of downstream gene transcription, autophagy, and other cellular processes. A deeper understanding of this network, particularly the identification of tissue-specific receptor targets, combined with leveraging clinical experience from existing inhibitor development, will provide a critical foundation for advancing precision anti-fibrotic therapies.

### 4.5 Other intracellular and extracellular signals

Beyond cytokines, endogenous metabolites and gaseous signaling molecules within the tissue microenvironment also exert critical effects via the PI3K/AKT pathway. Sphingolipid metabolites, such as sphingosine-1-phosphate (S1P) and its analog dihydro-sphingosine-1-phosphate (dhS1P), robustly activate the PI3K/AKT-mTOR axis by binding to their receptor S1PR (a GPCR). This axis drives the synthesis of pro-fibrotic factors and modulates ECM degradation by regulating the MMP/TIMP balance, playing a pivotal role in processes like cardiac remodeling. Notably, the S1P signaling network is highly complex, often interacting synergistically with other pathways such as ERK and p38 MAPK while directly affecting cardiomyocytes. This highlights the pleiotropic nature of metabolite signaling across different cell types and pathological contexts (Magaye et al., 2021). Similarly, gaseous signaling molecules like hydrogen sulfide (H_2_S) alleviate atrial fibrosis by activating the PI3K/AKT-eNOS pathway in CFs (Xue et al., 2020).

Recent studies indicate that PI3K/AKT signaling activity in fibroblasts is regulated by multiple peptides and their associated proteins. These regulators interact through complex molecular networks, ultimately influencing ECM remodeling and fibrotic progression. Due to the large number of substances involved and space limitations, the relevant mechanisms are summarized in Table 1.

**TABLE 1:** Mechanisms by which other peptides and related proteins mediate PI3K/AKT in fibroblasts.

Key substance	Study	Signal cascades	Cell type	Disease	Result
Ac-SD_D_K_D_P	Qiu et al. (2020)	PI3K/AKT	TGF-β-HFL-1	IPF	Ac-SD_D_K_D_P reduces the phosphorylation of AKT and mTOR, thereby inhibiting fibrosis
ZNF451	Peng et al. (2024)	PI3K/AKT	BML-MLFs	Pulmonary fibrosis	ZNF451 depletion upregulates PDGF-B, triggering PI3K signaling in LFs and enhancing COL1A1, COL3A1, and FN1 production
PRDX1	Sun et al. (2023)	PI3K/AKT-JNK/Smad	BLM-MLFs	Pulmonary fibrosis	PRDX1 knockdown induces ROS accumulation and mitochondrial dysfunction, and also activates the PI3K/AKT-JNK/Smad signaling axis to upregulate COL1/3 transcript levels to maintain ECM homeostasis
STX11 and SNAP25	Huang G. et al. (2024)	PI3K/AKT	TGF-β/BLM-HLFs	Pulmonary fibrosis	Overexpression of either STX11 or SNAP25 antagonizes TGF-β1-induced AKT phosphorylation and reverses HLFs activation and pro-fibrotic effects
CIB1	Hu et al. (2022)	PI3K/AKT	Ang II-MCFs	Myocardial fibrosis	CIB1 activation of PI3K/AKT upregulates the expression of proteins in collagen production and induces myocardial fibrosis, leading to cardiac insufficiency after myocardial infarction
iNOS	You et al. (2024)	PI3K/AKT	Ang II -MCFs	Myocardial fibrosis	iNOS overexpression promotes increased PI3K/AKT phosphorylation Levels, promotes cardiac fibroblast proliferation and migration, increases COL1/3 expression, and leads to MF
ANP	Zhu et al. (2024)	PI3K/AKT-TN-C	Ang II-RCFs	Myocardial fibrosis	ANP inhibits Ang II-induced PI3K/AKT-mediated TN-C signaling pro-fibrotic and pro-inflammatory pathways impair cardiomyocyte adhesion to connective tissue while promoting myofibroblast recruitment and aggregation
ADAMTS8	Zha et al. (2022)	PI3K/AKT-YAP	MI/TAC -RCFs	Cardiac Fibrosis	Activation of the PI3K/AKT and MAPK signaling pathways resulted in the upregulation of YAP, promoting the proliferation of CFs and the expression of collagen and α-SMA.
DOCK2	Hu et al. (2024)	PI3K/AKT and Wnt/β-catenin	MI/Ang II-MCFs	Cardiac Fibrosis	DOCK2 silencing inhibited the activation of the PI3K/AKT pathway and reduced the expression of α-SMA, COL1/3
Vaspin	Ji et al. (2022)	PI3K/AKT	MI/TAC/Ang II-NRCFs	Cardiac Fibrosis	Vaspin inhibits the PI3K/AKT pathway, reduces COL1/3 expression, and alleviates CF.
Apelin	Zhong et al., 2020; Shi WQ et al., 2024	PI3K/AKT	Ang II-RCFs and HKFs	Cardiac Fibrosis and Keloid	Apelin-13 inhibited mRNA expression and fibrosis of COL1/3, TGF-β, and α-SMC in CFs. Apelin-activated APJ-PI3K/AKT-CREB1-TGF-β1 in KFs to promote COL1 and FN production and scar formation
Ang II	Chen et al. (2023)	PI3K/AKT-mTOR	HSFs	HS	Reduction of Ang II decreases PI3K/AKT-mTOR to activate autophagy and inhibits MMP-2/9-mediated metabolic imbalance in the ECM.
DJ-1	Lv et al. (2023)	PI3K/AKT-mTOR	HKFs	Keloid	SNO-DJ-1 promotes keloid formation by inhibiting PTEN phosphatase activity via transnitrosylation and activating the PI3K/AKT-mTOR pathway
FAPα	He et al. (2024)	PI3K/AKT	MDFs	Skin fibrosis	Activation of the PI3K/AKT pathway by FAPα promotes MDFs’ proliferation, ECM secretion, and FMT.
Irisin	Wu et al. (2023)	PI3K/AKT	D-gal-SMFs	Skeletal muscle fibrosis	Irisin overexpression attenuates D-galactose-induced senescence, redox imbalance, and fibrosis by regulating the PI3K/AKT signaling pathway
EFEMP1	Shi WQ et al. (2024)	PI3K/AKT	GPSFs	Scleral remodeling and form-deprivation myopia	Activation of PI3K/AKT by EFEMP1 with EGFR activation further promotes transcription and translation of the downstream MMP2 gene
OPN	Lou et al. (2021)	PI3K/AKT	GO patients-OFs	GO	OPN promoted the proliferation and migration of OFs by activating the PI3K/Akt signaling pathway and induced the expression of VEGF and COL1

IPF, idiopathic pulmonary fibrosis; GO:Graves’ Ophthalmopathy; HS, hyperplastic scar; HFL-1, Human fetal lung fibroblast-1; MLFs, Mouse lung fibroblasts; CFs, Cardiac fibroblasts; HCFs, Human cardiac fibroblasts; RCFs, Human cardiac fibroblasts; MCFs, Mouse cardiac fibroblasts; SMFs, Skeletal muscle fibroblasts; HSFs, Hypertrophic scar fibroblasts; HDFs, Human dermal fibroblasts; LFs, Lung fibroblasts; OFs, Orbital Fibroblasts; NRCFs; Neonatal rat cardiac fibroblasts; KFs, keloid fibroblasts; GPSFs, guinea pig scleral fibroblasts; MDFs, Mouse dermal fibroblasts; RDFs, Rat dermal fibroblasts; CIB1, Calcium and integrin binding protein 1; ADAMTS8, A disintegrin and metalloproteinase with thrombospondin motifs 8; DOCK2, Dedicator of Cytokinesis 2; FAPα, fibroblast activation protein-α; EFEMP1, EGF-containing fibulin extracellular matrix protein 1; iNOS, inducible nitric oxide synthase; DJ-1, PARK; 7 gene encodes a protein of 189 amino acids; Vaspin, An inhibitor of visceral adipose tissue-derived serine protease; ZNF451, Zinc finger protein 451; Ang II, Angiotensin II; COL1/3, Collagen type I and collagen type III; MMP-2/9, Matrix metalloproteinases-2; and matrix metalloproteinases-9; Ac-SDKP, N-acetyl-seryl-aspartyl-lysyl-proline; ZNF451, Zinc finger protein 451; ANP, atrial natriuretic peptide; DOCK2, Dedicator of cytokinesis 2; CIB1, calcium and integrin binding protein; ADAMTS8:A disintegrin and metalloproteinase with thrombospondin motifs 8; YAP, yes-associated protein; SNO, S-nitrosylation; OPN, osteoblast protein; PRDX1, Peroxiredoxin-1; The prefix “pre-” denotes treatment with the corresponding substance or pathological model.

Taken together, the PI3K/AKT pathway functions as the signaling command center for fibroblasts, integrating diverse inputs from metabolites, hormones, ECM stiffness, and genetic material. Future anti-fibrotic therapies should move beyond indiscriminately shutting down this central hub. Instead, they must learn to precisely interpret and modulate these signals. By developing smart drugs responsive to the tissue microenvironment, we can specifically block pathogenic ECM remodeling driven by these signals, ultimately achieving physiological repair of fibrotic lesions.

## 5 Potential therapeutics and drugs targeting the PI3K/AKT signaling pathway in fibroblasts

Currently, clinical studies targeting the PI3K/AKT pathway in fibroblasts are limited in the context of fibrotic and tissue remodeling diseases. However, extensive preclinical research has focused on modulating the PI3K/AKT pathway in fibroblasts from various tissues to treat animal models of ECM-related diseases. Consequently, this area of research holds significant potential. These studies have identified numerous potential therapeutic agents and novel treatment strategies, including those in the field of regenerative medicine. This section aims to summarize current potential drugs targeting the PI3K/AKT signaling cascade in the context of fibroblasts, categorized into direct-acting agents, upstream modulators, fibroblast-specific targets, extracellular vesicle-based therapies, and clinically promising drugs with unknown targets.

### 5.1 Drugs that directly target the PI3K/AKT signaling cascade

Recent preclinical studies have identified numerous potential direct binding targets within the PI3K/AKT-mTOR signaling cascade, with modulation of this pathway’s activation or inhibition emerging as a promising strategy (Table 2).

**TABLE 2:** Drugs acting directly on the PI3K/AKT signaling cascade in fibroblasts in preclinical studies.

Agent	Specific target	Cell types	Disease	Description of the role	Common adverse effects	Ref
LY294002	PI3K	LFs, corneal myofibroblasts, HBF, etc.	Pulmonary fibrosis, airway fibrosis, corneal stromal fibrosis, etc.	Inhibition of fibroblast proliferation and FN and COL1/3 production	A certain degree of toxicity	Hu et al., 2020; Zhang et al., 2016; Li et al., 2024; Sinha et al., 2025
GSK2126458	PI3K/mTOR	HLFs	IPF	By inhibiting the AKT phosphorylation pathway, the proliferation and COL1 synthesis of primary human lung fibroblasts were significantly reduced	Diarrhea, high blood sugar, nausea	Mercer et al., 2016; Kottmann et al., 2015; Lukey et al., 2019
TET	AKT1/PIK3CA	NIH-3T3 cell	Silicosis	Inhibits the migration, proliferation, and differentiation of pulmonary fibroblasts	Poor water solubility, low oral bioavailability, short half-life, and potential toxicity to organs such as the liver and kidneys	Ma et al. (2024)
AZD6738	PI3Kδ	HConFs	Subconjunctival scarring	Inhibits TGF-β1-induced fibrotic responses, including myofibroblast activation and the synthesis of associated ECM proteins such as FN, COL 1, and COL 4	Fatigue, nausea, loss of appetite, anemia, thrombocytopenia, vomiting	Huang et al., 2022a; Kwon et al., 2022
CUDC-907	PI3K/HDAC1-6	LFs, CAFs	Lung and tumor fibrosis	Inhibition of fibroblast/CAFs cell proliferation, migration, and apoptosis	Diarrhea, thrombocytopenia, fatigue, nausea, constipation, vomiting	Zhang et al., 2020; Oki et al., 2017
APi	AKT1/GSK3β	MCFs	Cardiac fibrosis	Inhibits TGFβ1-induced proliferation, cell viability, expression of α-SMA and COL1/3 proteins, as well as phosphorylation of AKT 1 and GSK 3β in MCFs	Undescribed	Kan et al., 2024; Borges et al., 2022
OP-D	AKT1/AKT2	HLF-1	Pulmonary fibrosis	Inhibits pulmonary EMT and excessive ECM deposition, promotes pulmonary fibroblast apoptosis, and blocks the differentiation of pulmonary fibroblasts into myofibroblasts	Undescribed	Bao et al. (2023)
AS-IV	AKT1	NRK-49F	Renal fibrosis	Suppression of α-SMA, COL, and FN Expression in NRK-52 E, NRK-49 F, and UIRI Rats	Undescribed	Yu L. et al. (2022)
JHF	EGFR/AKT1	HFLs	Pulmonary fibrosis	Inhibits fibroblast activation	Undescribed	Shao et al. (2022)
Peimine	PIK3CD/AKT (1-3)	NIH-3T3 cell	Pulmonary fibrosis	Inhibits fibroblast activation and glycolysis	Undescribed	Li Y. et al. (2025)

Api, Apigenin; OP-D, Ophiopogonin-D; AS-IV, Astragaloside-IV; JHF, jinshui huanxian formula; TET, tetrandrine; IPF, idiopathic pulmonary fibrosis; LFs, Lung fibroblasts; PLFs, Primary lung fibroblasts; HFLs, Human embryonic lung fibroblasts; CFs, Cardiac fibroblasts; MCFs, Mouse cardiac fibroblasts; HCFs, Human cardiac fibroblasts; HBF, human bronchial fibroblasts; HDFs, Human dermal fibroblasts.

#### 5.1.1 PI3K/AKT targeted drugs

LY294002, one of the earliest PI3K inhibitors, suppresses fibroblast proliferation and the production of FN and COL1/3 in pulmonary fibrosis models (Hu et al., 2020; Zhang et al., 2016). It inhibits AKT phosphorylation and downstream TRPC1 regulation in human bronchial fibroblasts (HBF) during airway fibrosis induced by environmental particulate matter (Li et al., 2024). Additionally, it inhibits PI3K in corneal myofibroblasts and nearly completely reverses ECM deposition in the cornea (Sinha et al., 2025). LY294002 is a weak inhibitor with micromolar potency and significant off-target effects on other kinases, rendering it unsuitable as a specific PI3K therapeutic (Workman et al., 2010). It is now primarily used in control experiments in cellular and animal studies.

Ceralasertib (AZD6738) is an ATR inhibitor, but at high doses, it inhibits the PI3Kδ subtype in HConFs. By modulating the PI3K/AKT pathway, it induces apoptosis in HConFs and regulates VEGFA expression through eNOS phosphorylation, thereby influencing ocular fibrotic responses (Huang et al., 2022b). In clinical trials, AZD6738 is primarily used for cancer treatment (Yap et al., 2021; Ring et al., 2023). In a Phase II trial for advanced gastric cancer (NCT03780608), combination therapy with AZD6738 and durvalumab demonstrated antitumor activity, achieving partial remission or disease stabilization in some patients and extending progression-free survival and overall survival (Kwon et al., 2022). Adverse events included hypertension and anemia as serious incidents, with diarrhea and fatigue as common non-serious incidents, all manageable through dose adjustments. Thus, AZD6738 holds significant potential for future fibrotic disease regulation (Huang et al., 2022a).

CUDC-907 is a novel small-molecule compound with dual inhibitory activity against PI3K and histone deacetylase (HDAC). HDACs play a crucial role in epigenetic regulation by removing acetyl groups from histones, compacting chromatin structure, and thereby influencing gene transcription (Qian et al., 2012). When HDAC activity is inhibited, histone acetylation levels increase, altering chromatin conformation to facilitate transcription factor access and modulate gene expression (Smith et al., 2019). Zhang et al. (Zhang et al., 2020) demonstrated that this dual-target inhibitor induces dephosphorylation of AKT-mTOR and SMAD2/3 proteins, thereby suppressing the transcription and translation of fibrosis-associated genes. Furthermore, inducing high histone H3 acetylation in CAFs downregulates TGF-β1-induced expression of multiple HDAC isoforms. These combined pharmacological effects effectively suppress excessive ECM protein secretion by fibroblasts, potentially improving fibrosis in the lungs and tumors. In a clinical study (NCT01742988), CUDC-907 demonstrated tolerable safety as monotherapy and in combination with rituximab. Adverse events were predominantly mild to moderate, primarily gastrointestinal and hematologic, with no severe inflammatory or toxic symptoms observed (Oki et al., 2017). CUDC-907 thus represents a promising therapeutic candidate warranting further clinical investigation.

Tetrandrine (TET), a dibenzylisoquinoline alkaloid derived from Stephania tetrandra, has been shown to exhibit multiple pharmacological effects, including anticancer and anti-silicosis activities (Liu et al., 2023; Xue et al., 2023; Bhagya and Chandrashekar, 2016). Recent studies indicate that it functions as a targeted drug for PIK3CA and AKT1 (Ma et al., 2024). In models of pulmonary fibrosis and silicosis treatment, targeting the PI3K/AKT signaling pathway inhibits pulmonary fibroblast proliferation and migration while promoting apoptosis, thereby alleviating silica-induced pulmonary fibrosis. This therapeutic effect is observed in both early and advanced stages of the disease (Ma et al., 2024). TET has demonstrated efficacy in both experimental models and silicosis patients (Miao et al., 2013). However, its poor water solubility, low oral bioavailability, and potential hepatotoxicity and nephrotoxicity limit its clinical application. Addressing these limitations is crucial for its future development.

Jinshui Huanxian formula (JHF), a 10-herb compound, has undergone molecular docking analysis indicating that its active constituents—tangeretin, isosinensetin, and peimine—may inhibit EGFR and subsequently PI3K/AKT signaling HFLs. This leads to suppressed fibroblast activation and reduced expression of fibrosis-related proteins. Molecular docking further reveals that these compounds bind to AKT1 (Shao et al., 2022). Further studies revealed that peimine inhibits PI3K/AKT-mediated metabolic responses via PFKFB3, thereby suppressing ECM expression in LFs (Li Y. et al., 2025). This research also highlights that peimine interacts with PIK3CD and AKT1-3 as target points. Currently, research on these drugs remains in its early stages, with side effects and more detailed mechanisms requiring further exploration and testing.

#### 5.1.2 PI3K/mTOR targeted drugs

mTOR inhibitors represent a well-established therapeutic class, with sirolimus and its analogues (e.g., everolimus) widely used in the treatment of various diseases. In the field of fibrosis, sirolimus has shown significant anti-fibrotic properties in animal models (Jin et al., 2014). By inhibiting mTORC1 activity in fibroblasts, sirolimus downregulates α-SMA and COL1 expression, thereby reducing pathological damage in pulmonary and hepatic fibrosis (Woodcock et al., 2019). Preclinical studies have shown that everolimus induces autophagy in fibroblasts, reducing postoperative knee joint fibrosis and collagen deposition (Liu Y. et al., 2020). These findings lay the groundwork for expanding its application in fibroblast-targeted therapies for fibrotic diseases. Notably, the controversy surrounding rapamycin stems from its selective inhibition of mTORC1, which fails to effectively block the primary downstream role of 4E-BP1 in collagen synthesis and may induce adverse reactions (Wang J. et al., 2022; Woodcock et al., 2019). Thus, the use of these drugs as monotherapy appears inadequate for treating fibrosis, highlighting the need to explore their combination with other agents.

Omipalimib (GSK2126458) exhibits multi-targeted antifibrotic effects as a dual PI3K/mTOR pathway inhibitor. *In vitro* studies confirm its ability to significantly reduce proliferation and COL1 synthesis in primary HLFs by targeting PI3K/mTOR (Mercer et al., 2016; Kottmann et al., 2015). Preliminary clinical translational research (NCT01725139) further validated the drug’s safety in IPF patients through a double-blind, placebo-controlled dose-ranging study. Adverse events (AEs) primarily included diarrhea, hyperglycemia, and nausea, with no serious AEs reported and no participants withdrawing early due to AEs (Lukey et al., 2019). However, this trial was limited by a small sample size, insufficient statistical power, a short study duration lacking long-term follow-up, and the absence of a formal anti-fibrotic assessment. Therefore, additional trials are needed to address these limitations and better validate the drug’s applicability.

#### 5.1.3 AKT/GSK3β targeted therapy

Integrated studies employing network pharmacology, molecular docking, dynamics, and multi-omics validation have identified apigenin (APi) (Kan et al., 2024) and the steroidal saponin ophiopogonin-D (OP-D) from Salvia miltiorrhiza (Bao et al., 2023) as direct, selective inhibitors of the AKT/GSK3β axis in fibroblasts. These compounds bind competitively to the ATP-binding pockets of AKT1/2 and GSK3β, thereby blocking downstream phosphorylation cascades. This mechanism inhibits EMT and ECM deposition in models of cardiac and pulmonary fibrosis. Their capacity to reverse established fibrotic phenotypes *in vivo*, coupled with low toxicity and synergistic activity with classical PI3K inhibitors, positions APi and OP-D as promising natural product leads for developing targeted anti-fibrotic therapies. Notably, APi is currently under investigation in several clinical trials for various human diseases (e.g., NCT05999682, NCT03139227). Although no trials have yet investigated API for ECM-related diseases, the reported absence of adverse reactions in one clinical study (NCT03526081) (Borges et al., 2022) supports its favorable safety profile and potential for clinical translation.

Additionally, a network pharmacology study revealed that Astragaloside IV (AS-IV) can bind AKT1 and restore GSK3β activity, thereby reducing fibroblast proliferation and decreasing the synthesis of ECM components such as COL1 and FN. Conversely, AS-IV promotes β-catenin degradation in epithelial cells, inhibits Wnt/β-catenin pathway activation, reduces PAI-1 and Snail expression, and suppresses EMT and renal fibrosis progression (Yu L. et al., 2022). Theoretically, this dual-functional pharmacology enables the compound to achieve dual-cell-type inhibition with a single agent, effectively curbing abnormal matrix expansion. It may emerge as a PI3K/AKT inhibitor capable of simultaneously suppressing myofibroblast-mediated fibrogenesis and epithelial-derived ECM amplification.

### 5.2 PI3K/AKT signaling upsream receptor modulator

As outlined in Section 4, the numerous targets capable of activating the PI3K/AKT signaling pathway in fibroblasts provide a robust theoretical basis for the discussion in this chapter. Substances associated with these targets may all participate in fibroblast regulation of the ECM. Consequently, various receptor modulators, including RTKs, GPCRs, cytokine receptors, and serine/threonine kinase receptors upstream of PI3K, are promising candidates for future targeted drug development. Given the extensive number and broad range of relevant targets, this section will focus on presenting research progress in fibroblast-related studies, specifically highlighting inhibitors of commonly studied receptors such as TGF-βR and IGF-1R.

#### 5.2.1 TGF-β receptor inhibitors

TGF-βR inhibitors have advanced through multiple preclinical and clinical stages in antifibrotic and anticancer applications (Peng et al., 2022). Many of these agents, acting on fibroblasts, have demonstrated potent antifibrotic effects with significant therapeutic potential.

Galunisertib (LY2157299) is a promising inhibitor of TGF-β type I kinase (ALK5). Studies indicate that Galunisertib inhibits TGF-β-induced fibrotic DFs(Peterson et al., 2022), reducing fibrotic scarring while accelerating dermal wound closure. In clinical trials, combination with gemcitabine extended overall survival in patients with unresectable pancreatic cancer (Melisi et al., 2018), while monotherapy demonstrated acceptable safety profiles (Kelley et al., 2019; Santini et al., 2019).

Vactosertib (EW-7197) is another ALK5 inhibitor. In three-dimensional renal fibrosis chip models and mouse models, Vactosertib exerts anti-fibrotic effects by inhibiting TGF-β signaling pathways in renal tissue cells (e.g., fibroblasts), regulating inflammatory cytokine expression, and promoting angiogenesis (Jang et al., 2025). Thus, it holds potential therapeutic value for renal fibrosis. Clinical studies indicate that Vactosertib demonstrates good tolerability and no safety concerns when combined with Durvalumab in patients with metastatic non-small cell lung cancer (NSCLC) and urothelial carcinoma (Cho et al., 2024). Furthermore, combination therapy with the immunomodulator Pomalidomide also shows safety (Malek et al., 2018; Malek et al., 2019).

Topical application of the ALK5 inhibitor A-83-01 to skin wounds suppresses myofibroblast proliferation, effectively preventing burn wound contraction without delaying wound closure (Sun et al., 2014). However, clinical data on A-83-01 remain limited, warranting further investigation.

Epigallocatechin gallate (EGCG) is a significant drug, confirmed as a dual inhibitor of ALK5 and lysyl oxidase-like 2 (LOXL2) (Wei et al., 2017). A follow-up study to an early clinical trial found that EGCG treatment significantly downregulated TGF-β1 pathway genes (COL1A1, CTHRC1, SERPINE1) and sFRP2 gene expression in HLFs, while also inhibiting pro-inflammatory and stress pathways in fibroblasts (Cohen et al., 2024). As this drug remains in early clinical trials, further information is still needed. Notably, a multicenter, double-blind, placebo-controlled, dose-ranging Phase I study of oral EGCG for IPF patients is currently underway (NCT05195918).

Although TGF-β-targeting drugs show promise in preclinical models, their clinical efficacy has often fallen short of expectations. This discrepancy may arise from the pleiotropic nature of TGF-β signaling and the complexity of its regulatory networks. Exploring combination therapies that integrate TGF-β inhibition with other targeted agents may yield improved outcomes.

#### 5.2.2 IGF-1 receptor inhibitors

In the context of targeting fibroblasts, IGF-1R inhibitors have been extensively studied as therapeutic agents for autoimmune diseases, particularly active thyroid-associated ophthalmopathy (TAO) (Morshed et al., 2022; Kim et al., 2024; Mohyi and Smith, 2018). This is because the primary therapeutic target cells for diseases like TAO are located in OFs and orbital adipocytes (OAs) (Cui et al., 2023).

Linsitinib, a selective IGF-1R inhibitor, has been shown in OF-related studies to effectively suppress proliferation and HA secretion. This mechanism involves concurrent attenuation of the PI3K/AKT and ERK signaling pathways, providing a novel pharmacological rationale for targeted TAO therapy (Lee et al., 2024). Linsitinib advanced to phase II/III oncology trials but failed to improve overall survival or progression-free survival across multiple solid tumors, showing no objective response advantage. However, it exhibited a favorable safety profile (von Mehren et al., 2020; Fassnacht et al., 2015; Oza et al., 2018). It is now being repurposed for TAO, with an ongoing phase 2b trial (NCT05276063) for active, moderate-to-severe disease.

Teprotumumab (RV 001, R1507), a fully human monoclonal antibody, binds with high specificity to the IGF-1R extracellular domain, blocking ligand binding and receptor activation (Kurzrock et al., 2010). Early studies revealed that it inhibits fibroblast AKT phosphorylation by blocking both IGF-1R and TSHR, while simultaneously reducing proinflammatory cytokine release. This provides a molecular rationale for the therapeutic efficacy of this antibody in TAO and demonstrates its potential for targeting OFs(Chen et al., 2014). Recent studies have confirmed and clarified that teprotumumab inhibits OFs’ *in vitro* HA secretion by suppressing TSHR/IGF-1R crosstalk rather than by blocking the binding of autoantibodies (TSAb) to IGF-1R. In recent years, teprotumumab has demonstrated efficacy in treating TAO patients (Smith et al., 2017; Douglas et al., 2020). Common adverse events include muscle spasms, nausea, diarrhea, fatigue, and hyperglycemia, though these were generally mild during treatment.

It is worth emphasizing that IGF-1R inhibitors have previously experienced large-scale failures in cancer treatment. For instance, in pancreatic cancer therapy, a Phase III clinical trial (NCT01231347) of Ganitumab—a monoclonal antibody inhibiting IGF-1R activity—combined with gemcitabine failed to improve patient survival rates (Fuchs et al., 2015). Similarly, a Phase II trial of cixutumumab (an IGF-1R mAb antagonist) combined with erlotinib and gemcitabine for stage IV pancreatic cancer also failed to extend progression-free survival (Philip et al., 2014). Key reasons for failure include the high homology between IGF-1R and insulin receptor (IR), where inhibitors may disrupt insulin signaling and cause side effects (e.g., hyperglycemia). Furthermore, the complex regulation of IGF and IGF-binding proteins (IGFBPs) secreted by stromal cells in the tumor microenvironment makes overcoming resistance challenging with single IGF-1R blockade (Mutgan et al., 2018). More specific drugs must be developed to optimize combination therapy regimens (Philippou et al., 2017). In TAO, however, the IGF-1R inhibitor’s target organs are concentrated locally within the orbit, featuring a relatively simple microenvironment. Local administration reduces systemic side effects, making this a key factor in its successful application. Therefore, targeted therapy for fibroblasts requires direct inhibition of fibroblast IGF-1R signaling via highly specific inhibitors, combined with fibroblast deactivation drugs to reduce IGF ligand sources. Concurrently, biomarker screening should identify sensitive populations to ultimately achieve the goal of suppressing pathological fibroblast function and improving the microenvironment in diseases like TAO and fibrosis.

### 5.3 Targeting fibroblast signature markers

Challenges facing anti-fibrotic drugs targeting fibroblasts include a lack of tissue specificity and an inability to precisely target specific fibroblast subtypes. While these drugs inhibit abnormal fibroblasts, they may also impair normal tissue function and induce systemic toxicity, thereby limiting their clinical application. Fibroblast-specific targets (e.g., FAPα) exhibit distinct expression specificity, being highly expressed on activated fibroblasts in pathological states while maintaining low expression in normal tissues and quiescent fibroblasts. Precision intervention strategies based on these targets enable targeted enrichment at lesion sites, specifically suppressing abnormal fibroblast activation while reducing systemic off-target toxicity. This approach offers a key direction for enhancing the specificity and safety of anti-fibrotic and anti-tumor therapies.

FAPα, a type II transmembrane protease highly expressed on activated fibroblasts in fibrotic tissues and tumor microenvironments, is closely linked to PI3K/AKT pathway activation. It promotes fibroblast activation by degrading ECM components and regulating cytokines, making it a long-standing attractive target (Hamson et al., 2014; Wang J. et al., 2014; Kelly et al., 2012). Initial targeting strategies focused on depleting CAFs in cancer therapy, encompassing non-conjugated monoclonal antibodies, immunoconjugates, vaccines, and radionuclide-based therapies (Zou et al., 2025). However, early monoclonal antibody clinical trials demonstrated limited efficacy (Scott et al., 2003). Novel approaches like chimeric antigen receptor T cells (CAR-T cells) and drug conjugates have shown promising results in cancer models (Shahvali et al., 2023), leading to the extension of these targeted therapies to other diseases like fibrosis and inflammation. In models of cardiac fibrosis, CAR-T cells have been shown to selectively eliminate overactivated fibroblasts, reduce ECM deposition, and ultimately reverse fibrosis and restore tissue function (Rurik et al., 2022). Similarly, CAR-macrophages (CAR-Ms) have been found to specifically phagocytose FAP^+^ fibroblasts *in vitro* and infiltrate myocardial fibrotic regions *in vivo*, effectively eliminating activated fibroblasts and significantly reducing myocardial fibrosis following ischemia-reperfusion injury, thereby improving cardiac systolic and diastolic function without notable short-term toxicity (Wang J. et al., 2024). Additionally, Talabostat, a small-molecule dipeptidyl peptidase inhibitor targeting FAPα, has shown concentration-dependent suppression of activated fibroblast viability in both healthy and SSc fibroblasts, while also inhibiting fibroblast migration in SSc patients (Pashaei et al., 2024). Comparable effects have been observed in fibroblasts from pulmonary fibrosis. (Egger et al., 2017). Importantly, therapeutic approaches such as antibody-radiopharmaceutical conjugates and enzyme inhibitor-radiopharmaceutical conjugates complement molecular imaging techniques like positron emission tomography (PET) with FAPI tracers (FAPI PET). As a diagnostic adjunct, FAPI PET accurately identifies patients with high FAPα expression, thereby enhancing the precision of targeting. This integrated strategy holds significant potential for broader applications in preclinical and clinical trials targeting fibroblasts in diseases such as cancer and fibrosis (Ferreira et al., 2021; Telo et al., 2022; Li K. et al., 2025; Mori et al., 2023).

In addition to FAP, other notable targets include PDGFRα/β and CD26 (Xin et al., 2020; Riccetti et al., 2024; Rimal et al., 2022). Targeted therapies based on fibroblast-specific markers have been extensively reviewed in the literature (Chen et al., 2021a; Li K. et al., 2025; Zou et al., 2025; Klinkhammer et al., 2018). Many of these intervention strategies are linked to the regulation of the PI3K/AKT pathway, collectively forming the central research framework for fibroblast-targeted therapies.

### 5.4 Extracellular vesicle-based therapies

Beyond conventional pharmacological interventions, EVs sourced from diverse cellular origins have increasingly garnered substantial interest as a novel cell-free therapeutic modality. These EVs, which are membrane-bound vesicles released by cells, transport a range of bioactive molecules, including proteins and nucleic acids, and function as pivotal mediators of intercellular signaling. EVs originating from distinct cell types can specifically modulate the PI3K/AKT signaling cascade by transferring their cargo to fibroblasts, thus exerting dual-directional regulatory influences on ECM metabolism (Qin et al., 2023; Wu et al., 2024).

For example, adipose-derived stem cell-derived EVs (ADSCs-EVs) can activate the PI3K/AKT pathway by transmitting signals such as PDGF-AA. This delays fibroblast senescence and promotes the synthesis of ECM components like COL1/3 and elastin, thereby helping to maintain tissue elasticity (Guo et al., 2020; Wang et al., 2021a). Conversely, epidermal stem cell-derived EVs (EpiSC-EVs) deliver miR-203a-3p, which targets and suppresses PI3KCA expression. This downregulates AKT/mTOR phosphorylation levels and effectively inhibits myofibroblast activation, thereby demonstrating anti-fibrotic potential (Zhao et al., 2025).

In the context of epithelial and endothelial cells, human amniotic epithelial cell-derived exosomes (hAECs-Exos) have been shown to significantly enhance fibroblast proliferation and collagen synthesis in diabetic wound models by augmenting PI3K/AKT-mTOR pathway activity, thereby facilitating tissue repair (Wei F. et al., 2020). Similarly, endothelial cell-derived EVs (ED-EVs) not only activate the PI3K/AKT-mTOR cascade but also induce YAP dephosphorylation and nuclear translocation in a synergistic manner, thereby accelerating wound healing through multiple mechanisms (Wei P. et al., 2020).

Current EVs therapies are primarily in Phase I clinical trials, with a focus on safety validation. An *in vitro* study has shown that platelet-derived extracellular vesicles (pEVs) carry substances such as IGF and TGF-β, which can be taken up by fibroblasts (DFs). These pEVs promote fibroblast proliferation, migration, and endothelial angiogenesis by activating the ERK and AKT signaling pathways (Johnson et al., 2023). Subsequent Phase I human trials have demonstrated that a single subcutaneous injection of ligand-based exosome affinity-purified pEVs is safe and well-tolerated in healthy volunteers. However, no significant acceleration in wound healing was observed in the normal wounds of healthy individuals, although further validation of efficacy is required in patients with delayed wound healing (Johnson et al., 2023).

In summary, the essence of EV-based therapies hinges on the biological functions of their contents. By selecting different cellular sources, it is possible to either upregulate or downregulate the PI3K/AKT pathway in fibroblasts. This approach holds immense potential for inhibiting pathological fibrosis or promoting physiological tissue repair, depending on therapeutic needs. This opens up broad prospects for developing a new generation of precision-targeted therapeutic products for fibroblasts.

### 5.5 Other drugs with potential

In addition to the previously mentioned drugs, we have identified several compounds that show promise in preclinical studies. These compounds collectively influence ECM remodeling by modulating PI3K/AKT signaling in various fibroblast types through diverse mechanisms, as detailed in Table 3.

**TABLE 3:** Drugs targeting the PI3K/AKT signaling pathway in fibroblasts without a defined target.

Disease	Agent	Specific target	Target cell	Description of the role	Common adverse effects	Ref
Pulmonary fibrosis	Rhy	Unclear	HFL-1	Reduces the abnormal overexpression of FN, Col I, and α-SMA induced by TGF-β1-mediated PI3K/AKT activation	Long-term oral use may cause liver and kidney damage and can affect the cardiovascular and central nervous systems by crossing the blood-brain barrier	Wang X. et al. (2023)
Pulmonary fibrosis	ISL	Unclear	MRC-5 cell	By inhibiting PI3K/AKT/mTOR pathway activation to induce autophagy, TGF-β1-induced proliferation, migration, and expression of fibrosis markers in MRC-5 cells were suppressed	Undescribed	He et al. (2020)
Pulmonary fibrosis	ZBM	Unclear	MRC-5 cell	Inhibits PI3K/AKT thereby suppresses FOXO3 phosphorylation, restoring its original function and inhibiting fibroblast proliferation	Undescribed	Feng et al. (2025)
Pulmonary fibrosis	NTS, TS	Unclear	MPLFs	By blocking both the PI3K/AKT-HIF-1α and PI3K/PAK-RAF-ERK-HIF-1α pathways through the pyrrolo [1,2-a]azepine core, it inhibits SDF-1-mediated M2 macrophage polarization, thereby disrupting the positive feedback loop of fibrosis activation in pulmonary fibrosis	Undescribed; known side effects of nicotinamide include gastrointestinal discomfort (nausea, vomiting), skin flushing, and dizziness	Fu et al. (2023)
Pulmonary fibrosis、RA	BAI	Unclear	RPLFs, RAFLS	Inhibits PI3K/AKT activation alleviates pulmonary fibrosis and fibroblast proliferation	It is generally safe and well tolerated	Zhang et al., 2022b; Zhao et al., 2020a; Li et al., 2021
IPF	NEN	Unclear	HLFs	Inhibits TGF-β1-induced EMT and ECM accumulation	Undescribed	Pei et al. (2022)
IPF	SIN	Unclear	HLF-1	Inhibits TGF-β1/Smad 3, PI3K/AKT, and NF-κB pathways suppresses the activation, migration, and proliferation of HFL-1 cells	No direct side effects have been explicitly mentioned, but attention should be paid to the potential hepatotoxicity and nephrotoxicity of Chinese herbal extracts	Yao et al., 2024; Huang et al., 2019; Liu et al., 2018
Silicosis	CEL-07	Unclear	NIH-3T3 cell	Inhibits the expression of PI3K/AKT-mediated fibrotic factors α-SMA and COL1/3, and promotes fibroblast apoptosis by increasing ROS accumulation	Not apparent; the parent compound Tripterygium wilfordii homofuran is known to exhibit hepatotoxicity, nephrotoxicity, hematotoxicity, and reproductive toxicity. CEL-07 exhibits reduced toxicity following structural optimization	Bai et al., 2024; Xiao et al., 2017
Myocardial fibrosis	Emodin	Unclear	CFs	Significantly suppresses oxidative stress and damage in cells, inhibits ANGII-induced proliferation of cardiac fibroblasts, downregulates activation of the PI3K/AKT-mTOR pathway, suppresses α-SMA expression, and promotes COL2 expression, thereby alleviating myocardial fibrosis and improving cardiac function	Hepatotoxicity and nephrotoxicity	Huang W. et al., 2024; Zhang C. et al., 2022
Cardiac fibrosis	Calycosin	Unclear	CFs	Activates PI3K/AKT to inhibit STAT3 phosphorylation, reduce MMP-9 expression, suppress the transformation of cardiomyocytes into myofibroblasts, and alleviate myocardial fibrosis	Cardiac toxicity	Wang J et al. (2022)
Diabetic wound healing	hAECs-Exos	Unclear	HDFs	Promotes angiogenesis and fibroblast function	Undescribed	Wei F. et al. (2020)
Diabetic wound healing	ADSCs-hEVs	Unclear	HLFs	Suppresses early inflammatory responses while promoting the secretion and expression of growth factors and extracellular matrix-related markers	Undescribed	Wang et al. (2021b)
Chronic non-healing wounds	Corylin	Unclear	NIH-3T3 cell	Co-activation of the PI3K/AKT and SIRT1/NF-κB pathways coordinates fibroblasts and macrophages to promote wound healing	undescribed	Xiu et al. (2023)
Skin wound healing	MY-1	MY-1 binds to the PTH1 receptor on the surface of fibroblasts	PRDFs	Enhances fibroblast migration, thereby increasing fibroblast aggregation in the early stages and leading to denser collagen deposition in the later stages	Undescribed	Zhou et al. (2024)
Skin wound healing	Rhein	Unclear	HDFs	Promoting skin wound healing and accelerating collagen maturation by activating the PI3K/AKT pathway	Low-dose short-term use appears to be relatively safe, whereas high-dose long-term administration may lead to liver and kidney toxicity and adverse reactions including gastrointestinal discomfort	Yang et al., 2024; Fu et al., 2024
Anti-aging	ADSC-CM	Unclear	HDFs	Increased expression of COL1/3, elastin, and TIMP-1, but decreased expression of MMP-1 and MMP-9 in irradiated and non-irradiated HDFs	Undescribed	Guo et al. (2020)
HS	EpiSC-EVs	Unclear	HLFs	Inhibits PIK3CA expression and excessive activation of the PI3K/AKT/mTOR pathway plays a crucial role in reducing scar formation	Undescribed	Zhao et al. (2025)

Rhy, Rhynchophylline; BAI, baicalein; ISL, isoliquiritigenin; NEN, niclosamide ethanolamine salt; ZBM, zhenbeimu; ESL, Elephantopus scaber Linn.; SIN, sinomenine; NTS, neostemonine; TS, hyakusogan; IPF, idiopathic pulmonary fibrosis; LFs, Lung fibroblasts; PLFs, Primary lung fibroblasts; MPLFs, Mouse primary lung fibroblasts; CFs, Cardiac fibroblasts; HCFs, human cardiac fibroblasts; HDFs, Human dermal fibroblasts RDFs, Rat dermal fibroblasts; PRDFs, Primary rat dermal fibroblasts; RAFLS, rheumatoid arthritis synovial fibroblasts; ADSCs-hEVs, HIF-1α overexpressing adipose stem cells extracellular vesicles; EpiSC-EVs, Epidermal stem cells-extracellular vesicles; hAECs-Exos, human amniotic epithelial cell exosomes; ED-EVs, Endothelial cells-extracellular vesicles; EMT, Epithelial-mesenchymal transition.

## 6 Delivery strategies and challenges targeting the PI3K/AKT pathway in fibroblasts

Investigation of targeted pharmaceuticals is undeniably significant. Nevertheless, the successful translation of biological insights into effective therapeutic strategies is equally essential, necessitating thoughtful consideration of precise, efficient, and secure drug delivery to the disease site.

Take pulmonary fibrosis treatment delivery as an illustrative example (Diwan et al., 2024). Oral administration, widely adopted for its convenience, entails drug absorption via the small intestine. However, it confronts several challenges, including compromised absorption of hydrophilic drugs, suboptimal lung targeting, gastrointestinal degradation, and systemic distribution, which can trigger adverse reactions such as diarrhea and rash (Alqahtani et al., 2021; Fala, 2015). Intravenous infusion circumvents the pulmonary mucosal barrier but is invasive and may lead to drug clearance by mononuclear phagocytes and indiscriminate biodistribution (Patton and Byron, 2007). In contrast, pulmonary inhalation delivery is a highly appealing targeting method, leveraging the lung’s expansive surface area and thin epithelial barrier to facilitate site-specific drug delivery and rapid therapeutic onset. This approach effectively bypasses first-pass metabolism and systemic toxicity, thereby lowering required drug doses, reducing administration frequency, and minimizing associated adverse reactions. Importantly, inhalation delivery enhances drug bioavailability and patient adherence, although challenges such as pulmonary toxicity, drug stability, and pulmonary defense mechanisms need to be addressed (Patton et al., 2004; Ray et al., 2020; Kuzmov and Minko, 2015). Over the past few years, nanocarriers, including liposomes and polymeric nanoparticles, have shown significant promise in preclinical drug delivery (Ferguson et al., 2023; Seo et al., 2016; Shahabadi et al., 2022). These advanced carriers enhance drug delivery efficacy by favorably modulating *in vitro* drug release, bioavailability, and safety, thus emerging as a crucial strategy to surmount the limitations of conventional delivery methods (van Rijt et al., 2014; Diwan et al., 2024).

Considering that the PI3K/AKT signaling pathway is a fundamental and extensively expressed mechanism in various cell types, imprecise delivery of PI3K/AKT modulators can trigger systemic reactions, such as metabolic disorders, immunosuppression, and skin toxicity, thus narrowing their clinical application scope. The unique physical microenvironment of fibrotic tissue acts as a natural barrier, as activated fibroblasts become embedded within dense ECM deposits, such as collagen, which they overproduce. This results in increased tissue stiffness and diffusion resistance, severely hindering effective drug penetration and accumulation. Moreover, the significant heterogeneity of fibroblasts means that pathogenic fibroblast subpopulations coexist with healthy subpopulations that maintain tissue homeostasis within fibrotic lesions. This underscores the need for therapeutic strategies with high cellular selectivity to prevent collateral damage to normal physiological functions. Utilizing nanoparticle carriers for drug delivery serves as a strategy to avoid systemic exposure when targeting fibroblasts, achieving favorable outcomes in numerous animal studies.

For example, a poly (lactic-co-glycolic acid)-polyvinyl alcohol (PLGA-PVA)-based nanocarrier system loaded with rhyzine (Rhy) enabled sustained pulmonary-targeted drug release via tracheal spraying, effectively addressing Rhy’s issues of low water solubility, short half-life, and high toxicity (Wang S. et al., 2023). Additionally, exploiting the tunable and biodegradable properties of tissue engineering materials like polycaprolactone (PCL), metformin (MET)-grafted PCL nanostructures (METG-PCLN) ensure stable and sustained drug release while acting as an effective physical barrier. By targeting dorsal root ganglion (DRG) cells to modulate epidural fibroblast fibrosis, this approach effectively mitigates epidural fibrosis (EF) (Xu et al., 2024). Moreover, integrating physical stimulation with biomaterials, such as targeting wounds with Fe3O4 nanoparticle-loaded exosomes guided by static magnetic fields, highlights the potential of physical techniques to enhance drug delivery precision (Wu et al., 2020). The underlying principle involves applying sustained micro-magnetic forces to cells via magnetic nanoparticles (MNPs) within a static magnetic field (SMF) to promote tissue regeneration. This combination of MNPs and SMF modulates fibroblast phenotypic polarization for optimal wound healing (Li et al., 2016).

Beyond nanoparticles, hydrogels constitute a specialized category of gel materials utilized in tissue repair. These materials employ water as a dispersing medium and create three-dimensional network structures via the physical or chemical crosslinking of hydrophilic polymers. Their extensive application is attributed to their exceptional tissue compatibility, minimal toxicity, hydrophilic three-dimensional porous structures, and capacity for water swelling (Serpico et al., 2023). In studies focused on fibroblasts, chitosan hydrogels loaded with bFGF and SDF-1 exhibit exceptional biocompatibility, self-healing capabilities, and sustained drug release. These hydrogels enhance the periurethral microenvironment by initially providing physical support and subsequently inducing the homing of endogenous BMSCs. The underlying mechanism involves promoting fibroblast collagen deposition and proliferation via the JAK-STAT signaling pathways, effectively alleviating stress urinary incontinence (SUI) symptoms in mice (Yang et al., 2023).

Importantly, nanoparticles and hydrogels can synergistically address each other’s limitations. The transport of nanomaterials from hydrogels is essential for effective drug delivery and tissue engineering. Moreover, nanoparticle release can be triggered by hydrogel degradation, influenced by factors such as particle size and hydrogel pore dimensions, and can be selectively controlled through the swelling of stimulus-responsive hydrogels (Jobdeedamrong and Crespy, 2024). One study integrated methacrylamide gelatin (GelMA) with nanoparticles, specifically liposomes. Liposomes functioned as the primary drug carrier for MY-1 (a novel short-chain PTH peptide), safeguarded from protease degradation in the wound microenvironment by the phospholipid bilayer structure. The hydrogel served as a sustained-release scaffold, stabilizing the liposomes and preventing rapid leakage. Additionally, the hydrogel’s porous structure and degradative properties established a dual sustained-release mechanism with the liposomes. This strategy circumvented the rapid depletion observed in traditional hydrogel drug delivery while extending the duration of MY-1’s therapeutic effect. The drug was delivered to rabbit dermal fibroblasts (RDFs), activating the PI3K/AKT-Rac1 signaling pathway to regulate cell migration, thereby achieving wound healing and enhanced tensile strength (Zhou et al., 2024).

Furthermore, nucleic acid-based gene therapies are progressing rapidly. Current clinical applications encompass miRNA mimics, miRNA inhibitors, artificially designed miRNAs, and miRNA therapies in combination with carriers. However, several challenges remain, including off-target effects, low delivery efficiency, difficulties in dose regulation (Diener et al., 2022), and resistance to serum degradation (Catela Ivkovic et al., 2017). Consequently, high-throughput screening to develop more precise and safer miRNA therapies is imperative. Additionally, other ncRNAs, including lncRNAs, circRNAs, and siRNAs, also hold therapeutic potential, although they face similar delivery challenges as miRNAs (Chen K. et al., 2022). Nanoparticles have been extensively investigated for delivering nucleic acids of varying sizes, such as siRNA, miRNA, and mRNA (Xiao et al., 2022; Metkar et al., 2024). Enhancing the safety of ncRNA therapies and minimizing immune side effects are crucial, and optimizing nucleic acid delivery systems can facilitate targeted therapy for fibroblasts (Nemeth et al., 2024).

The increasing emphasis on nanomedicine underscores the pivotal role of nanotechnology-based drug delivery systems in treating diverse diseases, facilitating enhanced control over drug release, pharmacokinetics, and pharmacodynamics (Shang et al., 2024; Zhang Q. et al., 2023; Shi et al., 2023). Notably, the significant heterogeneity of fibroblasts remains a major barrier to achieving precision therapy. To surmount this challenge, future research should concentrate on identifying surface markers unique to pathogenic fibroblast subpopulations (e.g., FAP-α). By leveraging these markers to develop corresponding monoclonal antibodies, specific peptide ligands, or nucleic acid aptamers as targeted modulators, and chemically coupling them to surface-modified nanocarriers, it is possible to establish active targeted delivery systems.

## 7 Conclusions and future directions

**FIGURE 6:**
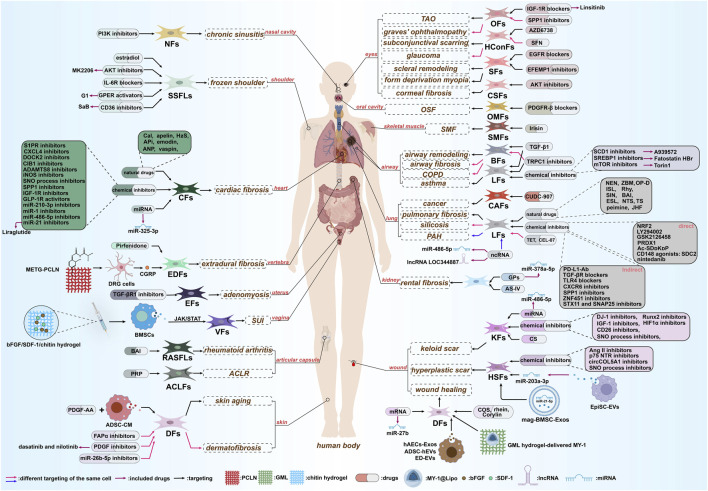
Summary of potential therapeutic agents targeting PI3K/AKT signaling in various types of fibroblasts. This section provides a comprehensive compilation of all therapeutic candidates discussed throughout this review that target fibroblast PI3K/AKT signaling pathways. These agents demonstrate potential for restoring homeostatic balance in tissue ECM through precise modulation of fibroblast activity. NFs, Nasal fibroblasts; SSFL, Shoulder synovial fibroblasts; CFs, Cardiac fibroblasts; EDFs, Epidural fibroblasts; EFs, Endometrial fibroblasts; VFs, Vaginal fibroblasts; RASFLs, Rheumatoid arthritis synovial fibroblasts; ACLFs, Anterior cruciate ligament fibroblasts; DFs, Dermal fibroblasts; OFs, Orbital Fibroblasts; HConFs, Human conjunctival fibroblasts; SFs, Scleral fibroblasts; CSFs, Corneal stromal fibroblasts; OMFs, Oral mucosal fibroblasts; SMFs, Skeletal muscle fibroblasts; BFs, Bronchial fibroblasts; LFs, Lung fibroblasts; CAFs, Cancer-associated fibroblasts; RFs, Renal fibroblasts; KFs, Keloid fibroblasts; HSFs, Hypertrophic scar fibroblasts; OSF, Oral submucous fibrosis; SMF, Skeletal muscle fibrosis; PAH, Pulmonary arterial hypertension; TAO, Thyroid-associated ophthalmopathy; SUI, Stress urinary incontinence; TRPC1, Transient receptor potential cation channel subfamily C member 1; Rhy, Rhynchophylline; OXA, Oxaliplatin; BAI, Baicalein; TET, Tetrandrine; ISL, Isoliquiritigenin; NEN, Niclosamide ethanolamine salt; Api, Apigenin; OP-D, Ophiopogonin-D; AS-IV, Astragaloside-IV; ZBM, Zhebeimu; ESL, Elephantopus scaber Linn.; SIN, sinomenine; NTS, Neotuberostemonine; TS, Tuberostemonine; JHF, Jinshui Huanxian formula; Cal, Calycosin; JHF, Jinshui Huanxian formula; Ac-SD_D_K_D_P, N-acetyl-seryl-aspartyl-lysyl-proline with Asp and Lys residues substituted with D-amino acids; CIB1, Calcium and integrin binding protein 1; DOCK2, Dedicator of cytokinesis 2; FAPα, fibroblast activation protein-α; EFEMP1, EGF-containing fibulin extracellular matrix protein 1; iNOS, inducible nitric oxide synthase; DJ-1, PARK 7 gene encodes a protein of 189 amino acids; Vaspin, An inhibitor of visceral adipose tissue-derived serine protease; ZNF451, Zinc finger protein 451; Ang II, Angiotensin II; Ac-SDKP, N-acetyl-seryl-aspartyl-lysyl-proline; ANP, Atrial natriuretic peptide; ADAMTS8, A disintegrin and metalloproteinase with thrombospondin motifs 8; OPN, Osteoblast protein; PRDX1, Peroxiredoxin-1.

In conclusion, the multidimensional regulatory mechanism involving fibroblasts, PI3K/AKT, and ECM offers a systematic and network-based framework for understanding the central role of fibroblast signaling in pathophysiology, while also systematically identifying numerous potential therapeutic targets (Figure 6). Systematic and comprehensive targeting of the PI3K/AKT pathway in distinct fibroblast subtypes can facilitate recovery from fibrosis or tissue damage at specific sites.

Notably, the precise details and therapeutic potential of this regulatory network remain largely unexplored. Future research should focus on the following areas:

Further elucidating fibroblast heterogeneity. Identifying the complex and diverse fibroblast subpopulations remains a significant challenge. There is an urgent need to prioritize the construction of high-resolution dynamic fibroblast subpopulation maps across tissues and disease states using single-cell multi-omics and spatial transcriptomics technologies. For instance, determining whether pro-fibrotic or inflammatory fibroblast subpopulations preferentially activate different upstream regulators of the PI3K pathway (e.g., IGF-1R, PDGFR) and identifying the unique receptor tyrosine kinase and GPCR profiles of pathogenic subtypes are essential prerequisites for developing cell-type-specific therapies that preserve homeostatic fibroblasts (Wei et al., 2021; Zou et al., 2025). Furthermore, designing therapies that precisely deliver PI3K/AKT pathway inhibitors to target cells based on disease subtype-specific markers can minimize off-target effects on steady-state ECM maintenance or other cell types.

Further research is needed to elucidate tissue-specific signaling actions. The contrasting functions of molecules like miR-486-5p—anti-fibrotic in skin but pro-fibrotic in the heart (Zhang X. et al., 2025; Xiao, 2021), and the opposing effects of the AKT-FOXO1 axis on ECM deposition in cardiac versus skin fibroblasts (Okada et al., 2015; Wang J. et al., 2022) highlight the profound influence of the tissue microenvironment. Cross-organ comparative biology collaborations are needed to decipher the extrinsic signaling and intrinsic epigenetic programming mechanisms that determine whether PI3K/AKT activation drives pathological ECM remodeling or regression. Understanding why pathway outputs exhibit context-dependent behavior is critical for predicting off-target effects of therapeutic agents in non-target tissues.

Further investigation into PI3K isoform specificity and pathway action differences is warranted. Detailed investigation of the distinct contributions of individual PI3K catalytic isoforms (p110α, β, γ, δ) to fibroblast biology and fibrosis remains limited in current fibroblast studies. Utilizing genetic and pharmacological tools to decipher their non-redundant functions in specific fibroblast subtypes is essential for designing isoform-selective modulators with superior therapeutic indices. Moreover, the cross-talk between the PI3K/AKT-NRF2 axis and pathways like TGF-β-SMAD signaling (Wu et al., 2023) requires mechanistic elucidation based on relevant protein expression data. Identifying direct molecular mediators and feedback loops is vital for understanding network-level regulation.

Beyond the well-established TGF-β pathway, promising signal crosstalk mechanisms in fibroblasts include emerging interactions between YAP/TAZ, PD-L1, and PI3K/AKT, warranting deeper investigation. As key effectors of the Hippo pathway, YAP/TAZ serve as critical hubs integrating mechanical and biochemical signals (Younesi et al., 2024; Zha et al., 2022). Studies indicate that AKT can directly phosphorylate YAP/TAZ in fibroblasts, thereby driving fibroblast activation, promoting mechanical memory formation, and sustaining myofibroblast survival (Younesi et al., 2024; Zha et al., 2022). On the other hand, although the association between PD-L1 and PI3K/AKT in fibroblasts is still in its exploratory phase, it demonstrates unique cross-dimensional regulatory value. Beyond its classical immunoregulatory functions, PD-L1 participates in cell-autonomous signaling. Targeting this axis holds promise for simultaneously disrupting the vicious cycle of fibrosis driven by immune dysregulation, enabling synergistic intervention in both intrinsic cellular function regulation and immune microenvironment remodeling. This approach circumvents the adaptive resistance potentially induced by monotherapy targeting PI3K/AKT (Lu et al., 2022; Zhao S. et al., 2024). In addition, leveraging the clinically established PD-L1 detection system, this strategy offers immediate translational support for stratified treatment and personalized medication in fibrotic patients (Zhao et al., 2023).

Establishing a translational research pathway. Current research is primarily driven by preclinical models. Therefore, building upon promising results in animal models, a more rigorous validation system must be established. Utilizing human patient tissues/organs and humanized animal models will validate target relevance and species-specific signaling differences. Key translational goals prioritize drug accessibility and functional significance in human disease.

Taken together, despite significant challenges, deepening insights into fibroblast PI3K/AKT biology equips us with tools to develop more precise fibrotic treatment and tissue remodeling strategies. By unraveling context-dependent signaling logic, leveraging fibroblast heterogeneity for precision targeting, and designing innovative delivery solutions, we hold the promise of transforming this pivotal signaling pathway from a biological enigma into novel therapies that improve patient outcomes in ECM-related diseases.

## Glossary

**ECM**: Extracellular matrix
**MMPs**: Matrix metalloproteinases
**TIMPs**: Tissue inhibitors of metalloproteinases
**α-SMA**: α-smooth muscle actin
**COL**: Collagen
**FN**: Fibronectin
**TGF-β**: Transforming growth factor-β
**FMT**: Fibroblast-to-myofibroblast transition
**PI3K**: Phosphatidylinositol 3-kinase
**PIP2**: Phosphatidylinositol 4,5-bisphosphate
**PIP3**: Phosphatidylinositol 3,4,5-trisphosphate
**mTORC2**: mTOR complex 2
**FOX**: Forkhead box
**COL1/3**: Collagen type I and collagen type III
**MMP-2/9**: Matrix metalloproteinases-2 and matrix metalloproteinases-9
**LC3**: Microtubule-associated protein light chain 3
**Bax**: Bcl-2 associated X protein
**Bim**: Bcl-2 interacting mediator of cell death
**Bad**: Bcl-2 associated death promoter
**VEGF**: Vascular endothelial growth factor
**LPS**: Lipopolysaccharide
**CaMKII**: Calmodulin-dependent kinase II
**BAI**: Baicalein
**LOX**: lysyl oxidase
**PDGFR**: Platelet-derived growth factor receptor
**GFs**: Growth factors
**qVICs**: quiescent valve interstitial cells
**SCD1**: stearoyl-coenzyme a desaturase 1
**YAP**: yes-associated protein
**IGF-1**: insulin-like growth factors-1
**HA**: hyaluronic acid
**OSF**: oral submucous fibrosis
**RA**: Rheumatoid arthritis
**ncRNA**: Non-coding RNA
**TME**: Tumor microenvironment
**IPF**: Idiopathic pulmonary fibrosis
**IPF**: Idiopathic pulmonary fibrosis
**MF**: Myocardial fibrosis
**SF**: Skin fibrosis
**AF**: Atrial fibrosis
**CF**: Cardiac Fibrosis
**GO**: Graves’ ophthalmopathy
**HS**: Hyperplastic scar
**FS**: Frozen shoulder
**SA-β-Gal**: Senescence associated- β-galactosidase
**LFs**: Lung fibroblasts
**PFB**: Primary lung fibroblasts
**MMVD**: Mucoviscidomatous mitral valve disease
**HFL-1**: Human fetal lung fibroblast-1
**CFs**: Cardiac fibroblasts
**HCFs**: Human cardiac fibroblasts
**HSFs**: Hypertrophic scar fibroblasts
**HDFs**: Human dermal fibroblasts
**OFs**: Orbital Fibroblasts
**NRCFs**: Neonatal rat cardiac fibroblasts
**HKFs**: Human keloid fibroblasts
**KFs**: Keloid fibroblasts
**SFs**: Scleral fibroblasts
**hCSFs**: Human corneal stromal fibroblasts
**MDFs**: Mouse dermal fibroblasts
**NHLF**: Normal human lung fibroblasts
**DFs**: Dermal fibroblasts
**HConFs**: Human conjunctival fibroblasts
**SFs**: Scleral fibroblasts
**SFLs**: Synovial fibroblasts
**RASFLs**: Rheumatoid arthritis synovial fibroblasts
**OASFLs**: Osteoarthritis synovial fibroblasts
**OFs**: Orbital Fibroblasts
**TAO**: Thyroid-associated ophthalmopathy
**CAFs**: Cancer-associated fibroblasts
**HBF**: Human bronchial fibroblasts
**CXCL16**: C-X-C chemokine ligand 16
**SDC2**: Syndecan-2
**p75NTR**: p75 neurotrophin receptor
**CS**: Chondroitin sulfate
**COS**: Chitooligosaccharide
**S1P/dhS1P**: Sphingosine-1-Phosphate/dihydro-Sphingosine-1-Phosphate
**Runx2**: Runt-related transcription factor 2
**FMT**: Fibroblast-to-myofibroblast transition
**HIF-1**: Hypoxia-inducible factor-1
**PRP**: Platelet-Rich Plasma
**SREBP1**: Sterol regulatory element binding protein 1
**Ac-SDKP**: N-acetyl-seryl-aspartyl-lysyl-proline
**ZNF451**: Zinc finger protein 451
**ANP**: atrial natriuretic peptide
**DOCK2**: Dedicator of cytokinesis 2
**CIB1**: calcium and integrin binding protein
**ADAMTS8**: A disintegrin and metalloproteinase with thrombospondin motifs 8
**YAP**: yes-associated protein
**SNO**: S-nitrosylation
**iNOS**: inducible nitric oxide synthase
**OPN**: osteoblast protein
**PRDX1**: peroxiredoxin-1
**EFEMP1**: EGF-containing fibulin extracellular matrix protein 1
**PD-L1**: Programmed death ligand 1
**NRF2**: Nuclear factor E2 related factor 2
**GSK3β**: Glycogen synthase kinase 3β
**IRS1**: Insulin receptor substrate 1
**ROS**: Reactive oxygen species
**C-RAPR1**: Cleaved poly (ADP-ribose) polymerase 1
**HE4**: Human epididymis protein 4
Rhy: Rhynchophylline
**OXA**: Oxaliplatin
**BAI**: Baicalein
**TET**: Tetrandrine
**ISL**: Isoliquiritigenin
**NEN**: Niclosamide ethanolamine salt
**Api**: Apigenin
**OP-D**: Ophiopogonin-D
**AS-IV**: Astragaloside-IV
**ZBM**: Zhebeimu
**ESL**: Elephantopus scaber Linn.
**SIN**: sinomenine
**NTS**: Neotuberostemonine
**TS**: Tuberostemonine
**JHF**: Jinshui Huanxian formula
**Cal**: Calycosin
**ERS**: Endoplasmic reticulum stress
**OS**: Oxidative stress
**ROS**: Reactive oxygen species
**PRDX**: Peroxiredoxin
**ADSCs-hEVs**: HIF-1α overexpressing adipose-derived stem cells-EVs
**EpiSC-EVs**: Epidermal stem cells-extracellular vesicles
**hAECs-Exos**: human amniotic epithelial cell exosomes
**ED-EVs**: Endothelial cells-extracellular vesicles
**HMDE**: hypoxic macrophage-derived exosomes
**GLP-1**: Glucagon-like peptide-1
**GPD-1L**: glycerol-3-phosphate dehydrogenase 1-like
**NDRG 2**: N-myc downstream regulated gene 2

## References

[B1] Abdul-Wahid A. Cydzik M. Fischer N. W. Prodeus A. Shively J. E. Martel A. (2018). 'Serum-derived carcinoembryonic antigen (CEA) activates fibroblasts to induce a local re-modeling of the extracellular matrix that favors the engraftment of CEA-Expressing tumor cells. Int. J. Cancer 143, 1963–1977. 10.1002/ijc.31586 29756328 PMC6128780

[B2] Ainola M. M. Mandelin J. A. Liljeström M. P. Li T. F. Hukkanen M. V. Konttinen Y. T. (2005). 'pannus invasion and cartilage degradation in rheumatoid arthritis: involvement of MMP-3 and interleukin-1beta. Clin. Exp. Rheumatol. 23, 644–650. 16173240

[B3] Alencar A. K. da Silva J. S. Lin M. Silva A. M. Sun X. Ferrario C. M. (2017). Effect of age, estrogen status, and late-life GPER activation on cardiac structure and function in the Fischer344×Brown Norway female rat. J. Gerontol. A Biol. Sci. Med. Sci. 72, 152–162. 10.1093/gerona/glw045 27006078 PMC5233906

[B4] Alqahtani M. S. Kazi M. Alsenaidy M. A. Ahmad M. Z. (2021). Advances in oral drug delivery. Front. Pharmacol. 12, 618411. 10.3389/fphar.2021.618411 33679401 PMC7933596

[B5] Aono Y. Kishi M. Yokota Y. Azuma M. Kinoshita K. Takezaki A. (2014). Role of platelet-derived growth factor/platelet-derived growth factor receptor axis in the trafficking of circulating fibrocytes in pulmonary fibrosis. Am. J. Respir. Cell Mol. Biol. 51, 793–801. 10.1165/rcmb.2013-0455OC 24885373

[B6] Bai Y. Liang C. Gao L. Han T. Wang F. Liu Y. (2024). Celastrol pyrazine derivative alleviates silicosis progression *via* inducing ROS-mediated apoptosis in activated fibroblasts. Molecules 29, 538. 10.3390/molecules29020538 38276616 PMC10820882

[B7] Baker D. J. Childs B. G. Durik M. Wijers M. E. Sieben C. J. Zhong J. (2016). Naturally occurring p16(Ink4a)-positive cells shorten healthy lifespan. Nature 530, 184–189. 10.1038/nature16932 26840489 PMC4845101

[B8] Bale L. K. Schafer M. J. Atkinson E. J. Le Brasseur N. K. Haak A. J. Oxvig C. (2022). Pregnancy-associated plasma protein-A (PAPP-A) is a key component of an interactive cellular mechanism promoting pulmonary fibrosis. J. Cell Physiol. 237, 2220–2229. 10.1002/jcp.30687 35098542 PMC9050837

[B9] Bao S. Chen T. Chen J. Zhang J. Zhang G. Hui Y. (2023). 'Multi-omics analysis reveals the mechanism of action of ophiopogonin D against pulmonary fibrosis. Phytomedicine 121, 155078. 10.1016/j.phymed.2023.155078 37734252

[B10] Bedard K. Krause K. H. (2007). The NOX family of ROS-generating NADPH oxidases: physiology and pathophysiology. Physiol. Rev. 87, 245–313. 10.1152/physrev.00044.2005 17237347

[B11] Bernstein Z. J. Shenoy A. Chen A. Heller N. M. Spangler J. B. (2023). Engineering the IL-4/IL-13 axis for targeted immune modulation. Immunol. Rev. 320, 29–57. 10.1111/imr.13230 37283511

[B366] Bettahi I. Sun H. Gao N. Wang F. Mi X. Chen W. (2014). Genome-wide transcriptional analysis of differentially expressed genes in diabetic, healing corneal epithelial cells: hyperglycemia-suppressed TGFβ3 expression contributes to the delay of epithelial wound healing in diabetic corneas. Diabetes 63, 715–727. 10.2337/db13-1260 24306208 PMC3900551

[B12] Bhagya N. Chandrashekar K. R. (2016). 'Tetrandrine--A molecule of wide bioactivity. Phytochemistry 125, 5–13. 10.1016/j.phytochem.2016.02.005 26899361

[B13] Bhatt J. Ghigo A. Hirsch E. (2025). PI3K/Akt in IPF: untangling fibrosis and charting therapies. Front. Immunol. 16, 1549277. 10.3389/fimmu.2025.1549277 40248697 PMC12004373

[B14] Bilanges B. Posor Y. Vanhaesebroeck B. (2019). PI3K isoforms in cell signalling and vesicle trafficking. Nat. Rev. Mol. Cell Biol. 20, 515–534. 10.1038/s41580-019-0129-z 31110302

[B15] Böhm M. Stegemann A. Paus R. Kleszczyński K. Maity P. Wlaschek M. (2025). 'endocrine controls of skin aging. Endocr. Rev. 46, 349–375. 10.1210/endrev/bnae034 39998423

[B16] Bonnans C. Chou J. Werb Z. (2014). Remodelling the extracellular matrix in development and disease. Nat. Rev. Mol. Cell Biol. 15, 786–801. 10.1038/nrm3904 25415508 PMC4316204

[B17] Bonner J. C. (2004). Regulation of PDGF and its receptors in fibrotic diseases. Cytokine Growth Factor Rev. 15, 255–273. 10.1016/j.cytogfr.2004.03.006 15207816

[B18] Borges G. Fong R. Y. Ensunsa J. L. Kimball J. Medici V. Ottaviani J. I. (2022). Absorption, distribution, metabolism and excretion of apigenin and its glycosides in healthy male adults. Free Radic. Biol. Med. 185, 90–96. 10.1016/j.freeradbiomed.2022.04.007 35452808

[B19] Calissi G. Lam E. W. Link W. (2021). Therapeutic strategies targeting FOXO transcription factors. Nat. Rev. Drug Discov. 20, 21–38. 10.1038/s41573-020-0088-2 33173189

[B20] Cao Y. Li Y. Fu S. C. Shen J. Zhang H. Jiang C. (2022). Platelet-rich plasma pretreatment protects anterior cruciate ligament fibroblasts correlated with PI3K-Akt-mTOR pathway under hypoxia condition. J. Orthop. Transl. 34, 102–112. 10.1016/j.jot.2022.02.002 35891713 PMC9283994

[B21] Catela Ivkovic T. Voss G. Cornella H. Ceder Y. (2017). microRNAs as cancer therapeutics: a step closer to clinical application. Cancer Lett. 407, 113–122. 10.1016/j.canlet.2017.04.007 28412239

[B22] Chan E. C. Peshavariya H. M. Liu G. S. Jiang F. Lim S. Y. Dusting G. J. (2013). Nox4 modulates collagen production stimulated by transforming growth factor β1 *in vivo* and *in vitro* . Biochem. Biophys. Res. Commun. 430, 918–925. 10.1016/j.bbrc.2012.11.138 23261430

[B23] Chang H. Y. Fan C. C. Chu P. C. Hong B. E. Lee H. J. Chang M. S. (2011). hPuf-A/KIAA0020 modulates PARP-1 cleavage upon genotoxic stress. Cancer Res. 71, 1126–1134. 10.1158/0008-5472.CAN-10-1831 21266351

[B24] Chen X. Cubillos-Ruiz J. R. (2021). Endoplasmic reticulum stress signals in the tumour and its microenvironment. Nat. Rev. Cancer 21, 71–88. 10.1038/s41568-020-00312-2 33214692 PMC7927882

[B25] Chen H. Mester T. Raychaudhuri N. Kauh C. Y. Gupta S. Smith T. J. (2014). 'Teprotumumab, an IGF-1R blocking monoclonal antibody inhibits TSH and IGF-1 action in fibrocytes. J. Clin. Endocrinol. Metab. 99, E1635–E1640. 10.1210/jc.2014-1580 24878056 PMC4154099

[B26] Chen Y. Li Z. Chen X. Zhang S. (2021a). 'Long non-coding RNAs: from disease code to drug role. Acta Pharm. Sin. B 11, 340–354. 10.1016/j.apsb.2020.10.001 33643816 PMC7893121

[B27] Chen Y. McAndrews K. M. Kalluri R. (2021b). Clinical and therapeutic relevance of cancer-associated fibroblasts. Nat. Rev. Clin. Oncol. 18, 792–804. 10.1038/s41571-021-00546-5 34489603 PMC8791784

[B28] Chen H. Xu K. Sun C. Gui S. Wu J. Wang S. (2023). 'Inhibition of ANGPT2 activates autophagy during hypertrophic scar formation *via* PI3K/AKT/mTOR pathway. An Bras Dermatol 98, 26–35. 10.1016/j.abd.2021.12.005 36272879 PMC9837657

[B29] Chen K. Li Y. Zhang X. Ullah R. Tong J. Shen Y. (2022). The role of the PI3K/AKT signalling pathway in the corneal epithelium: recent updates. Cell Death Dis. 13, 513. 10.1038/s41419-022-04963-x 35641491 PMC9156734

[B30] Chen Q. Zhang H. Yang Y. Zhang S. Wang J. Zhang D. (2022). Metformin Attenuates UVA-induced skin photoaging by suppressing mitophagy and the PI3K/AKT/mTOR pathway. Int. J. Mol. Sci. 23, 6960. 10.3390/ijms23136960 35805987 PMC9266365

[B31] Cheng Z. (2019). The FoxO-Autophagy axis in health and disease. Trends Endocrinol. Metab. 30, 658–671. 10.1016/j.tem.2019.07.009 31443842

[B367] Cho B. C. Lee K. H. Han J.-Y. Shim B. Kim J. Yang H. (2024). 605 vactosertib, a TGF-β signaling inhibitor, in combination with durvalumab increased mOS in≥ 2L treatment of patients with PD-L1-positive advanced NSCLC. BMJ Spec. J. 12 (Suppl. 2), A696. 10.1136/jitc-2024-SITC2024.0605

[B32] Chubanov V. Köttgen M. Touyz R. M. Gudermann T. (2024). TRPM channels in health and disease. Nat. Rev. Nephrol. 20, 175–187. 10.1038/s41581-023-00777-y 37853091

[B33] Ciesielska A. Matyjek M. Kwiatkowska K. (2021). TLR4 and CD14 trafficking and its influence on LPS-Induced pro-inflammatory signaling. Cell Mol. Life Sci. 78, 1233–1261. 10.1007/s00018-020-03656-y 33057840 PMC7904555

[B34] Clark J. G. Madtes D. K. Raghu G. (1993). Effects of platelet-derived growth factor isoforms on human lung fibroblast proliferation and procollagen gene expression. Exp. Lung Res. 19, 327–344. 10.3109/01902149309064350 8319603

[B35] Codogno P. Mehrpour M. Proikas-Cezanne T. (2011). Canonical and non-canonical autophagy: variations on a common theme of self-eating? Nat. Rev. Mol. Cell Biol. 13, 7–12. 10.1038/nrm3249 22166994

[B36] Cohen M. L. Brumwell A. N. Ho T. C. Garakani K. Montas G. Leong D. (2024). A fibroblast-dependent TGF-β1/sFRP2 noncanonical wnt signaling axis promotes epithelial metaplasia in idiopathic pulmonary fibrosis. J. Clin. Invest 134, e174598. 10.1172/JCI174598 38980870 PMC11405054

[B37] Conte E. (2022). Targeting monocytes/macrophages in fibrosis and cancer diseases: therapeutic approaches. Pharmacol. Ther. 234, 108031. 10.1016/j.pharmthera.2021.108031 34774879

[B38] Cui X. Wang F. Liu C. (2023). A review of TSHR- and IGF-1R-related pathogenesis and treatment of graves' orbitopathy. Front. Immunol. 14, 1062045. 10.3389/fimmu.2023.1062045 36742308 PMC9893276

[B39] Dai S. Xu M. Pang Q. Sun J. Lin X. Chu X. (2024). Hypoxia macrophage-derived exosomal miR-26b-5p targeting PTEN promotes the development of keloids. Burns Trauma 12, tkad036. 10.1093/burnst/tkad036 38434721 PMC10905499

[B40] Demaria M. Ohtani N. Youssef S. A. Rodier F. Toussaint W. Mitchell J. R. (2014). An essential role for senescent cells in optimal wound healing through secretion of PDGF-AA. Dev. Cell 31, 722–733. 10.1016/j.devcel.2014.11.012 25499914 PMC4349629

[B41] Deng S. Deng Q. Zhang Y. Ye H. Yu X. Zhang Y. (2019). Non-platelet-derived CXCL4 differentially regulates cytotoxic and regulatory T cells through CXCR3 to suppress the immune response to colon cancer. Cancer Lett. 443, 1–12. 10.1016/j.canlet.2018.11.017 30481563

[B42] Deng Z. Fan T. Xiao C. Tian H. Zheng Y. Li C. (2024). TGF-β signaling in health, disease, and therapeutics. Signal Transduct. Target Ther. 9, 61. 10.1038/s41392-024-01764-w 38514615 PMC10958066

[B43] Destaing O. Petropoulos C. Albiges-Rizo C. (2014). Coupling between acto-adhesive machinery and ECM degradation in invadosomes. Cell Adh Migr. 8, 256–262. 10.4161/cam.28558 24727371 PMC4198349

[B44] Diener C. Keller A. Meese E. (2022). Emerging concepts of miRNA therapeutics: from cells to clinic. Trends Genet. 38, 613–626. 10.1016/j.tig.2022.02.006 35303998

[B45] Disser N. P. Sugg K. B. Talarek J. R. Sarver D. C. Rourke B. J. Mendias C. L. (2019). 'Insulin-like growth factor 1 signaling in tenocytes is required for adult tendon growth. Faseb J. 33, 12680–12695. 10.1096/fj.201901503R 31536390 PMC6902672

[B46] Diwan R. Bhatt H. N. Beaven E. Nurunnabi M. (2024). 'Emerging delivery approaches for targeted pulmonary fibrosis treatment. Adv. Drug Deliv. Rev. 204, 115147. 10.1016/j.addr.2023.115147 38065244 PMC10787600

[B47] Douglas R. S. Kahaly G. J. Patel A. Sile S. Thompson E. H. Z. Perdok R. (2020). Teprotumumab for the treatment of active thyroid eye disease. N. Engl. J. Med. 382, 341–352. 10.1056/NEJMoa1910434 31971679

[B48] Driskell R. R. Watt F. M. (2015). Understanding fibroblast heterogeneity in the skin. Trends Cell Biol. 25, 92–99. 10.1016/j.tcb.2014.10.001 25455110

[B49] Ebi H. Costa C. Faber A. C. Nishtala M. Kotani H. Juric D. (2013). PI3K regulates MEK/ERK signaling in breast cancer *via* the Rac-GEF, P-Rex1. Proc. Natl. Acad. Sci. U. S. A. 110, 21124–21129. 10.1073/pnas.1314124110 24327733 PMC3876254

[B50] Egger C. Cannet C. Gérard C. Suply T. Ksiazek I. Jarman E. (2017). 'Effects of the fibroblast activation protein inhibitor, PT100, in a murine model of pulmonary fibrosis. Eur. J. Pharmacol. 809, 64–72. 10.1016/j.ejphar.2017.05.022 28506908

[B51] Ehrlich H. P. Desmoulière A. Diegelmann R. F. Cohen I. K. Compton C. C. Garner W. L. (1994). 'Morphological and immunochemical differences between keloid and hypertrophic scar. Am. J. Pathol. 145, 105–113. 8030742 PMC1887298

[B52] Fala L. (2015). 'Ofev (Nintedanib): first tyrosine kinase inhibitor approved for the treatment of patients with idiopathic pulmonary fibrosis. Am. Health Drug Benefits 8, 101–104. 26629273 PMC4665065

[B53] Fang Z. Meng Q. Xu J. Wang W. Zhang B. Liu J. (2023). Signaling pathways in cancer-associated fibroblasts: recent advances and future perspectives. Cancer Commun. (Lond) 43, 3–41. 10.1002/cac2.12392 36424360 PMC9859735

[B54] Fang C. Zeng Z. Ye J. Ni B. Zou J. Zhang G. (2025). Progress of mesenchymal stem cells affecting extracellular matrix metabolism in the treatment of female stress urinary incontinence. Stem Cell Res. Ther. 16, 95. 10.1186/s13287-025-04220-w 40001265 PMC11863768

[B55] Fassnacht M. Berruti A. Baudin E. Demeure M. J. Gilbert J. Haak H. (2015). 'Linsitinib (OSI-906) *versus* placebo for patients with locally advanced or metastatic adrenocortical carcinoma: a double-blind, randomised, phase 3 study. Lancet Oncol. 16, 426–435. 10.1016/S1470-2045(15)70081-1 25795408

[B56] Feng S. Xu G. Ding Q. Shi Y. (2025). 'Fritillaria thunbergii miq. Extract ameliorated experimental pulmonary fibrosis partly through the PI3K/AKT/FOXO signalling pathway. J. Ethnopharmacol. 343, 119445. 10.1016/j.jep.2025.119445 39938765

[B57] Ferguson L. T. Ma X. Myerson J. W. Wu J. Glassman P. M. Zamora M. E. (2023). Mechanisms by which liposomes improve inhaled drug delivery for alveolar diseases. Adv. Nanobiomed Res. 3, 2200106. 10.1002/anbr.202200106 37266328 PMC10231510

[B58] Ferreira C. Rosenkrans Z. Bernau K. Moore M. Valla F. Batterton J. (2021). “Targeting activated fibroblasts for non-invasive detection of lung fibrosis,” in Society of nuclear medicine.

[B59] Finkel T. Holbrook N. J. (2000). Oxidants, oxidative stress and the biology of ageing. Nature 408, 239–247. 10.1038/35041687 11089981

[B60] Frangogiannis N. G. (2021). Cardiac fibrosis. Cardiovasc Res. 117, 1450–1488. 10.1093/cvr/cvaa324 33135058 PMC8152700

[B61] Fredriksson L. Li H. Eriksson U. (2004). The PDGF family: four gene products form five dimeric isoforms. Cytokine Growth Factor Rev. 15, 197–204. 10.1016/j.cytogfr.2004.03.007 15207811

[B62] Fu S. Song X. Hu Y. Zhu Q. Lv X. Tang X. (2023). 'Neotuberostemonine and tuberostemonine ameliorate pulmonary fibrosis through suppressing TGF-β and SDF-1 secreted by macrophages and fibroblasts *via* the PI3K-dependent AKT and ERK pathways. Chin. J. Nat. Med. 21, 527–539. 10.1016/S1875-5364(23)60444-3 37517820

[B63] Fu Y. Yang L. Liu L. Kong L. Sun H. Sun Y. (2024). Rhein: an updated review concerning its biological activity, pharmacokinetics, structure optimization, and future pharmaceutical applications. Pharm. (Basel) 17, 1665. 10.3390/ph17121665 39770507 PMC11679290

[B64] Fuchs C. S. Azevedo S. Okusaka T. Van Laethem J. L. Lipton L. R. Riess H. (2015). A phase 3 randomized, double-blind, placebo-controlled trial of ganitumab or placebo in combination with gemcitabine as first-line therapy for metastatic adenocarcinoma of the pancreas: the GAMMA trial. Ann. Oncol. 26, 921–927. 10.1093/annonc/mdv027 25609246 PMC4804122

[B65] Geletu M. Adan H. Niit M. Arulanandam R. Carefoot E. Hoskin V. (2022). 'Modulation of akt vs Stat3 activity by the focal adhesion kinase in non-neoplastic mouse fibroblasts. Exp. Cell Res. 411, 112731. 10.1016/j.yexcr.2021.112731 34270980

[B66] Glaviano A. Foo A. S. C. Lam H. Y. Yap K. C. H. Jacot W. Jones R. H. (2023). 'PI3K/AKT/mTOR signaling transduction pathway and targeted therapies in cancer. Mol. Cancer 22, 138. 10.1186/s12943-023-01827-6 37596643 PMC10436543

[B67] Gu X. Jiang Y. N. Wang W. J. Zhang J. Shang D. S. Sun C. B. (2020). Comprehensive circRNA expression profile and construction of circRNA-related ceRNA network in cardiac fibrosis. Biomed. Pharmacother. 125, 109944. 10.1016/j.biopha.2020.109944 32062386

[B68] Guo S. Wang T. Zhang S. Chen P. Cao Z. Lian W. (2020). Adipose-derived stem cell-conditioned medium protects fibroblasts at different senescent degrees from UVB irradiation damages. Mol. Cell Biochem. 463, 67–78. 10.1007/s11010-019-03630-8 31602539

[B69] Guo Z. Su W. Zhou R. Zhang G. Yang S. Wu X. (2021). Exosomal MATN3 of urine-derived stem cells ameliorates intervertebral disc degeneration by antisenescence effects and promotes NPC proliferation and ECM synthesis by activating TGF-β. Oxid. Med. Cell Longev. 2021, 5542241. 10.1155/2021/5542241 34136064 PMC8175180

[B70] Guo N. Wang X. Xu M. Bai J. Yu H. Zhang Le. (2024). PI3K/AKT signaling pathway: molecular mechanisms and therapeutic potential in depression. Pharmacol. Res. 206, 107300. 10.1016/j.phrs.2024.107300 38992850

[B71] Hamson E. J. Keane F. M. Tholen S. Schilling O. Gorrell M. D. (2014). 'understanding fibroblast activation protein (FAP): substrates, activities, expression and targeting for cancer therapy. Proteomics Clin. Appl. 8, 454–463. 10.1002/prca.201300095 24470260

[B72] Han Z. Zhang X. Liu C. Lu M. Wang J. Nie Y. (2023). Analysis of long noncoding RNAs expression profiles in the human cardiac fibroblasts with cardiac fibrosis. Biochem. Biophys. Res. Commun. 660, 73–81. 10.1016/j.bbrc.2023.04.019 37068391

[B73] Hao H. Yan S. Zhao X. Han X. Fang N. Zhang Y. (2022). Atrial myocyte-derived exosomal microRNA contributes to atrial fibrosis in atrial fibrillation. J. Transl. Med. 20, 407. 10.1186/s12967-022-03617-y 36064558 PMC9446866

[B74] Hao C. Wei Y. Meng W. Zhang J. Yang X. (2025). PI3K/AKT/mTOR inhibitors for hormone receptor-positive advanced breast cancer. Cancer Treat. Rev. 132, 102861. 10.1016/j.ctrv.2024.102861 39662202

[B75] Hashemi M. Taheriazam A. Daneii P. Hassanpour A. Kakavand A. Rezaei S. (2023). Targeting PI3K/Akt signaling in prostate cancer therapy. J. Cell Commun. Signal 17, 423–443. 10.1007/s12079-022-00702-1 36367667 PMC10409967

[B76] Haspel J. A. Choi A. M. (2011). Autophagy: a core cellular process with emerging links to pulmonary disease. Am. J. Respir. Crit. Care Med. 184, 1237–1246. 10.1164/rccm.201106-0966CI 21836133 PMC3262043

[B77] He S. Sharpless N. E. (2017). Senescence in health and disease. Cell 169, 1000–1011. 10.1016/j.cell.2017.05.015 28575665 PMC5643029

[B78] He Z. Zhu Y. Jiang H. (2009). 'Toll-like receptor 4 mediates lipopolysaccharide-induced collagen secretion by phosphoinositide3-kinase-Akt pathway in fibroblasts during acute lung injury. J. Recept Signal Transduct. Res. 29, 119–125. 10.1080/10799890902845690 19519177

[B79] He Z. Gao Y. Deng Y. Li W. Chen Y. Xing S. (2012). 'Lipopolysaccharide induces lung fibroblast proliferation through toll-like receptor 4 signaling and the phosphoinositide3-kinase-Akt pathway. PLoS One 7, e35926. 10.1371/journal.pone.0035926 22563417 PMC3338545

[B80] He J. Peng H. Wang M. Liu Y. Guo X. Wang B. (2020). Isoliquiritigenin inhibits TGF-β1-induced fibrogenesis through activating autophagy *via* PI3K/AKT/mTOR pathway in MRC-5 cells. Acta Biochim. Biophys. Sin. (Shanghai) 52, 810–820. 10.1093/abbs/gmaa067 32638014

[B81] He Y. Sun M. M. Zhang G. G. Yang J. Chen K. S. Xu W. W. (2021). Targeting PI3K/Akt signal transduction for cancer therapy. Signal Transduct. Target Ther. 6, 425. 10.1038/s41392-021-00828-5 34916492 PMC8677728

[B82] He J. Fang B. Shan S. Li Q. (2024). Mechanical stiffness promotes skin fibrosis through FAPα-AKT signaling pathway. J. Dermatol Sci. 113, 51–61. 10.1016/j.jdermsci.2023.12.004 38155020

[B83] Hinz B. (2009). 'Tissue stiffness, latent TGF-beta1 activation, and mechanical signal transduction: implications for the pathogenesis and treatment of fibrosis. Curr. Rheumatol. Rep. 11, 120–126. 10.1007/s11926-009-0017-1 19296884

[B84] Hinz B. Lagares D. (2020). 'Evasion of apoptosis by myofibroblasts: a hallmark of fibrotic diseases. Nat. Rev. Rheumatol. 16, 11–31. 10.1038/s41584-019-0324-5 31792399 PMC7913072

[B85] Hinz B. McCulloch C. A. Coelho N. M. (2019). Mechanical regulation of myofibroblast phenoconversion and collagen contraction. Exp. Cell Res. 379, 119–128. 10.1016/j.yexcr.2019.03.027 30910400

[B86] Hou C. H. Tang C. H. Chen P. C. Liu J. F. (2021). Thrombospondin 2 promotes IL-6 production in osteoarthritis synovial fibroblasts *via* the PI3K/AKT/NF-κB pathway. J. Inflamm. Res. 14, 5955–5967. 10.2147/JIR.S314747 34803392 PMC8600055

[B87] Hoxhaj G. Manning B. D. (2020). The PI3K-AKT network at the interface of oncogenic signalling and cancer metabolism. Nat. Rev. Cancer 20, 74–88. 10.1038/s41568-019-0216-7 31686003 PMC7314312

[B88] Hsu H. S. Liu C. C. Lin J. H. Hsu T. W. Hsu J. W. Su K. (2017). Involvement of ER stress, PI3K/AKT activation, and lung fibroblast proliferation in bleomycin-induced pulmonary fibrosis. Sci. Rep. 7, 14272. 10.1038/s41598-017-14612-5 29079731 PMC5660192

[B89] Hu X. Xu Q. Wan H. Hu Y. Xing S. Yang H. (2020). 'PI3K-Akt-mTOR/PFKFB3 pathway mediated lung fibroblast aerobic glycolysis and collagen synthesis in lipopolysaccharide-induced pulmonary fibrosis. Lab. Invest 100, 801–811. 10.1038/s41374-020-0404-9 32051533

[B90] Hu G. Ding X. Gao F. Li J. (2022). 'calcium and integrin binding protein 1 (CIB1) induces myocardial fibrosis in myocardial infarction *via* regulating the PI3K/Akt pathway. Exp. Anim. 71, 1–13. 10.1538/expanim.21-0063 34349085 PMC8828404

[B91] Hu G. Chen J. Chen M. Yang K. Wang Y. Ma Z. (2024). Silencing DOCK2 attenuates cardiac fibrosis following myocardial infarction in mice *via* targeting PI3K/Akt and Wnt/β-Catenin pathways. J. Cardiovasc Transl. Res. 17, 1442–1454. 10.1007/s12265-024-10533-7 38990461

[B92] Hu X. Chen M. Tan B. Yang H. Li S. Li R. (2025). 'Vicenin-2 reduces inflammation and apoptosis to relieve skin photoaging *via* suppressing GSK3β. J. Photochem Photobiol. B 264, 113117. 10.1016/j.jphotobiol.2025.113117 39923642

[B93] Huang R. Y. Pan H. D. Wu J. Q. Zhou H. Li Z. G. Qiu P. (2019). Comparison of combination therapy with methotrexate and sinomenine or leflunomide for active rheumatoid arthritis: a randomized controlled clinical trial. Phytomedicine 57, 403–410. 10.1016/j.phymed.2018.12.030 30851515

[B94] Huang L. Wei Z. Wang X. Lan C. Zhu Y. Ye Q. (2022a). AZD6738 decreases intraocular pressure and inhibits fibrotic response in trabecular meshwork through CHK1/P53 pathway. Biochem. Pharmacol. 206, 115340. 10.1016/j.bcp.2022.115340 36347274

[B95] Huang L. Ye Q. Lan C. Wang X. Zhu Y. (2022b). AZD6738 inhibits fibrotic response of conjunctival fibroblasts by regulating checkpoint kinase 1/P53 and PI3K/AKT pathways. Front. Pharmacol. 13, 990401. 10.3389/fphar.2022.990401 36204234 PMC9530343

[B96] Huang, G G. Yang X. Yu Q. Luo Q. Ju C. Zhang B. (2024). Overexpression of STX11 alleviates pulmonary fibrosis by inhibiting fibroblast activation *via* the PI3K/AKT/mTOR pathway. Signal Transduct. Target Ther. 9, 306. 10.1038/s41392-024-02011-y 39523374 PMC11551190

[B97] Huang, W W. Zhou P. Zou X. Liu Y. Zhou L. Zhang Y. (2024). Emodin ameliorates myocardial fibrosis in mice by inactivating the ROS/PI3K/Akt/mTOR axis. Clin. Exp. Hypertens. 46, 2326022. 10.1080/10641963.2024.2326022 38507311

[B98] Hybertson B. M. Gao B. Bose S. K. McCord J. M. (2011). Oxidative stress in health and disease: the therapeutic potential of Nrf2 activation. Mol. Asp. Med. 32, 234–246. 10.1016/j.mam.2011.10.006 22020111

[B99] Hynes R. O. (2009). 'The extracellular matrix: not just pretty fibrils. Science 326, 1216–1219. 10.1126/science.1176009 19965464 PMC3536535

[B100] Imoto K. Okada M. Yamawaki H. (2018). Characterization of fibroblasts from hypertrophied right ventricle of pulmonary hypertensive rats. Pflugers Arch. 470, 1405–1417. 10.1007/s00424-018-2158-4 29860638

[B101] Irma J. Kartasasmita A. S. Kartiwa A. Irfani I. Rizki S. A. Onasis S. (2025). 'From growth factors to structure: PDGF and TGF-β in granulation tissue formation. A literature review. J. Cell Mol. Med. 29, e70374. 10.1111/jcmm.70374 40495632 PMC12152372

[B102] Jang S. Y. Hwang S. H. Choi Y. Kim W. Y. Park S. H. Kim W. M. (2025). 'Evaluation of the novel ALK5 inhibitor EW-7197 on therapeutic efficacy in renal fibrosis using a three-dimensional chip model. Kidney Res. Clin. Pract. 44, 612–625. 10.23876/j.krcp.23.324 39384357 PMC12245568

[B103] Ji M. Li Y. Liu Y. Ma G. (2022). 'Vaspin ameliorates cardiac remodeling by suppressing phosphoinositide 3-Kinase/Protein kinase B pathway to improve oxidative stress in heart failure rats. J. Cardiovasc Pharmacol. 80, 442–452. 10.1097/FJC.0000000000001291 36067399 PMC9439695

[B104] Jiang F. Wang M. Q. Zhang M. Y. Gu S. L. Xie Y. W. Huang Y. (2024). CPD-002, a novel VEGFR2 inhibitor, relieves rheumatoid arthritis by reducing angiogenesis through the suppression of the VEGFR2/PI3K/AKT signaling pathway. Int. Immunopharmacol. 131, 111850. 10.1016/j.intimp.2024.111850 38479157

[B105] Jin X. Dai H. Ding K. Xu X. Pang B. Wang C. (2014). 'Rapamycin attenuates bleomycin-induced pulmonary fibrosis in rats and the expression of metalloproteinase-9 and tissue inhibitors of metalloproteinase-1 in lung tissue. Chin. Med. J. Engl. 127, 1304–1309. 10.3760/cma.j.issn.0366-6999.20132749 24709185

[B106] Jinnin M. Ihn H. Asano Y. Yamane K. Trojanowska M. Tamaki K. (2006). Upregulation of tenascin-C expression by IL-13 in human dermal fibroblasts *via* the phosphoinositide 3-kinase/Akt and the protein kinase C signaling pathways. J. Invest Dermatol 126, 551–560. 10.1038/sj.jid.5700090 16374482

[B107] Jobdeedamrong A. Crespy D. (2024). Release and transport of nanomaterials from hydrogels controlled by temperature. Macromol. Rapid Commun. 45, e2400359. 10.1002/marc.202400359 38897179

[B108] Johnson J. Law S. Q. K. Shojaee M. Hall A. S. Bhuiyan S. Lim M. B. L. (2023). First-in-human clinical trial of allogeneic, platelet-derived extracellular vesicles as a potential therapeutic for delayed wound healing. J. Extracell. Vesicles 12, e12332. 10.1002/jev2.12332 37353884 PMC10290200

[B109] Juin A. Planus E. Guillemot F. Horakova P. Albiges-Rizo C. Génot E. (2013). Extracellular matrix rigidity controls podosome induction in microvascular endothelial cells. Biol. Cell 105, 46–57. 10.1111/boc.201200037 23106484

[B110] Kan H. Wang P. Yang Y. Jia H. Liu A. Wang M. (2024). Apigenin inhibits proliferation and differentiation of cardiac fibroblasts through AKT/GSK3β signaling pathway. J. Ethnopharmacol. 334, 118518. 10.1016/j.jep.2024.118518 38964628

[B111] Katayama Y. Naitoh M. Kubota H. Yamawaki S. Aya R. Ishiko T. (2020). 'Chondroitin sulfate promotes the proliferation of keloid fibroblasts through activation of the integrin and protein kinase B pathways. Int. J. Mol. Sci. 21, 1955. 10.3390/ijms21061955 32182995 PMC7139995

[B112] Keir M. E. Butte M. J. Freeman G. J. Sharpe A. H. (2008). PD-1 and its ligands in tolerance and immunity. Annu. Rev. Immunol. 26, 677–704. 10.1146/annurev.immunol.26.021607.090331 18173375 PMC10637733

[B113] Kelley R. K. Gane E. Assenat E. Siebler J. Galle P. R. Merle P. (2019). A phase 2 Study of galunisertib (TGF-β1 receptor type I inhibitor) and sorafenib in patients with advanced hepatocellular carcinoma. Clin. Transl. Gastroenterol. 10, e00056. 10.14309/ctg.0000000000000056 31295152 PMC6708671

[B114] Kelly T. Huang Y. Simms A. E. Mazur A. (2012). 'fibroblast activation protein-α: a key modulator of the microenvironment in multiple pathologies. Int. Rev. Cell Mol. Biol. 297, 83–116. 10.1016/B978-0-12-394308-8.00003-0 22608558

[B115] Kido D. Mizutani K. Takeda K. Mikami R. Matsuura T. Iwasaki K. (2017). 'Impact of diabetes on gingival wound healing *via* oxidative stress. PLoS One 12, e0189601. 10.1371/journal.pone.0189601 29267310 PMC5739411

[B116] Kim D. W. Kim S. Han J. Belday K. Li E. Mahoney N. (2024). Transcriptomic profiling of thyroid eye disease orbital fat demonstrates differences in adipogenicity and IGF-1R pathway. JCI Insight 9, e182352. 10.1172/jci.insight.182352 39704170 PMC11665563

[B117] Klinkhammer B. M. Floege J. Boor P. (2018). PDGF in organ fibrosis. Mol. Asp. Med. 62, 44–62. 10.1016/j.mam.2017.11.008 29155002

[B118] Kottmann R. M. Trawick E. Judge J. L. Wahl L. A. Epa A. P. Owens K. M. (2015). Pharmacologic inhibition of lactate production prevents myofibroblast differentiation. Am. J. Physiol. Lung Cell Mol. Physiol. 309, L1305–L1312. 10.1152/ajplung.00058.2015 26408551 PMC4669339

[B119] Kuppe C. Ibrahim M. M. Kranz J. Zhang X. Ziegler S. Perales-Patón J. (2021). 'Decoding myofibroblast origins in human kidney fibrosis. Nature 589, 281–286. 10.1038/s41586-020-2941-1 33176333 PMC7611626

[B120] Kurzrock R. Patnaik A. Aisner J. Warren T. Leong S. Benjamin R. (2010). A phase I study of weekly R1507, a human monoclonal antibody insulin-like growth factor-I receptor antagonist, in patients with advanced solid tumors. Clin. Cancer Res. 16, 2458–2465. 10.1158/1078-0432.CCR-09-3220 20371689

[B121] Kuzmov A. Minko T. (2015). 'Nanotechnology approaches for inhalation treatment of lung diseases. J. Control Release 219, 500–518. 10.1016/j.jconrel.2015.07.024 26297206

[B122] Kwon M. Kim G. Kim R. Kim K. T. Kim S. T. Smith S. (2022). Phase II study of ceralasertib (AZD6738) in combination with durvalumab in patients with advanced gastric cancer. J. Immunother. Cancer 10, e005041. 10.1136/jitc-2022-005041 35790315 PMC9258491

[B123] Lagares D. Hinz B. (2021). Animal and human models of tissue repair and fibrosis: an introduction. Methods Mol. Biol. 2299, 277–290. 10.1007/978-1-0716-1382-5_20 34028750

[B124] Lanzolla G. Marinò M. Menconi F. (2024). 'Graves disease: latest understanding of pathogenesis and treatment options. Nat. Rev. Endocrinol. 20, 647–660. 10.1038/s41574-024-01016-5 39039206

[B125] Lawrence J. Nho R. (2018). The role of the mammalian target of rapamycin (mTOR) in pulmonary fibrosis. Int. J. Mol. Sci. 19, 778. 10.3390/ijms19030778 29518028 PMC5877639

[B126] Lebel M. Cliche D. O. Charbonneau M. Adam D. Brochiero E. Dubois C. M. (2022). Invadosome Formation by lung fibroblasts in idiopathic pulmonary fibrosis. Int. J. Mol. Sci. 24, 499. 10.3390/ijms24010499 36613948 PMC9820272

[B127] LeBleu V. S. Neilson E. G. (2020). Origin and functional heterogeneity of fibroblasts. Faseb J. 34, 3519–3536. 10.1096/fj.201903188R 32037627

[B129] Lee K. T. Chen B. C. Liu S. C. Lin Y. Y. Tsai C. H. Ko C. Y. (2021). 'Nesfatin-1 facilitates IL-1β production in osteoarthritis synovial fibroblasts by suppressing miR-204-5p synthesis through the AP-1 and NF-κB pathways. Aging (Albany NY) 13, 22490–22501. 10.18632/aging.203559 34560673 PMC8507299

[B130] Lee J. Y. Lee S. B. Yang S. W. Paik J. S. (2024). 'Linsitinib inhibits IGF-1-induced cell proliferation and hyaluronic acid secretion by suppressing PI3K/Akt and ERK pathway in orbital fibroblasts from patients with thyroid-associated ophthalmopathy. PLoS One 19, e0311093. 10.1371/journal.pone.0311093 39693285 PMC11654993

[B131] Lendahl U. Muhl L. Betsholtz C. (2022). 'Identification, discrimination and heterogeneity of fibroblasts. Nat. Commun. 13, 3409. 10.1038/s41467-022-30633-9 35701396 PMC9192344

[B132] Levental I. Georges P. C. Janmey P. A. (2007). Soft biological materials and their impact on cell function. Soft Matter 3, 299–306. 10.1039/b610522j 32900146

[B133] Li X. Wei J. Aifantis K. E. Fan Y. Feng Q. Cui F. Z. (2016). 'Current investigations into magnetic nanoparticles for biomedical applications. J. Biomed. Mater Res. A 104, 1285–1296. 10.1002/jbm.a.35654 26779606

[B134] Li L. Gao H. Lou K. Luo H. Hao S. Yuan J. (2021). 'Safety, tolerability, and pharmacokinetics of oral baicalein tablets in healthy Chinese subjects: a single-center, randomized, double-blind, placebo-controlled multiple-ascending-dose study. Clin. Transl. Sci. 14, 2017–2024. 10.1111/cts.13063 34156161 PMC8504836

[B135] Li X. Ma X. Miao Y. Zhang J. Xi B. Li W. (2023). 'Duvelisib attenuates bleomycin-induced pulmonary fibrosis *via* inhibiting the PI3K/Akt/mTOR signalling pathway. J. Cell Mol. Med. 27, 422–434. 10.1111/jcmm.17665 36651446 PMC9889612

[B136] Li S. Qu L. Zhou L. Zhan N. Liu L. Ling Y. (2024). Biomass fuels related-PM(2.5) promotes lung fibroblast-myofibroblast transition through PI3K/AKT/TRPC1 pathway. Ecotoxicol. Environ. Saf. 276, 116309. 10.1016/j.ecoenv.2024.116309 38599156

[B137] Li K. Liu X. Lu R. Zhao P. Tian Y. Li J. (2025). Bleomycin pollution and lung health: the therapeutic potential of peimine in bleomycin-induced pulmonary fibrosis by inhibiting glycolysis. Ecotoxicol. Environ. Saf. 289, 117451. 10.1016/j.ecoenv.2024.117451 39626488

[B138] Li Q. Li Z. Luo T. Shi H. (2022). Targeting the PI3K/AKT/mTOR and RAF/MEK/ERK pathways for cancer therapy. Mol. Biomed. 3, 47. 10.1186/s43556-022-00110-2 36539659 PMC9768098

[B139] Li S. Ding X. Zhang H. Ding Y. Tan Q. (2022). 'IL-25 improves diabetic wound healing through stimulating M2 macrophage polarization and fibroblast activation. Int. Immunopharmacol. 106, 108605. 10.1016/j.intimp.2022.108605 35149293

[B140] Li Y. Liu Q. Jing X. Wang Y. Jia X. Yang X. (2025). Cancer-Associated fibroblasts: heterogeneity, cancer pathogenesis, and therapeutic targets. MedComm 6, e70292. 10.1002/mco2.70292 40656546 PMC12246558

[B141] Lin Y. Y. Ko C. Y. Liu S. C. Wang Y. H. Hsu C. J. Tsai C. H. (2021). 'miR-144-3p ameliorates the progression of osteoarthritis by targeting IL-1β: potential therapeutic implications. J. Cell Physiol. 236, 6988–7000. 10.1002/jcp.30361 33772768

[B142] Liu R. Chen H. Bai H. Zhang W. Wang X. Qin X. (2013). Suppression of nuclear factor erythroid 2-related factor 2 *via* extracellular signal-regulated kinase contributes to bleomycin-induced oxidative stress and fibrogenesis. Toxicol. Lett. 220, 15–25. 10.1016/j.toxlet.2013.03.034 23570914

[B368] Liu H. R. Xia Z. Y. Wang N. L. (2020). Sulforaphane modulates TGFβ2-induced conjunctival fibroblasts activation and fibrosis by inhibiting PI3K/Akt signaling. Int. J. Ophthalmol. 13, 1505–1511. 10.18240/ijo.2020.10.01 33078098 PMC7511376

[B143] Liu W. Zhang Y. Zhu W. Ma C. Ruan J. Long H. (2018). Sinomenine inhibits the progression of rheumatoid arthritis by regulating the secretion of inflammatory cytokines and monocyte/macrophage subsets. Front. Immunol. 9, 2228. 10.3389/fimmu.2018.02228 30319663 PMC6168735

[B144] Liu G. Cooley M. A. Jarnicki A. G. Borghuis T. Nair P. M. Tjin G. (2019). Fibulin-1c regulates transforming growth factor-β activation in pulmonary tissue fibrosis. JCI Insight 5, e124529. 10.1172/jci.insight.124529 31343988 PMC6777837

[B145] Liu T. Li K. Zhang Z. Peng J. Yang J. Law B. Y. K. (2023). Tetrandrine inhibits cancer stem cell characteristics and epithelial to mesenchymal transition in triple-negative breast cancer *via* SOD1/ROS signaling pathway. Am. J. Chin. Med. 51, 425–444. 10.1142/S0192415X23500222 36692485

[B146] Liu C. Khairullina L. Qin Y. Zhang Y. Xiao Z. (2024). 'Adipose stem cell exosomes promote mitochondrial autophagy through the PI3K/AKT/mTOR pathway to alleviate keloids. Stem Cell Res. Ther. 15, 305. 10.1186/s13287-024-03928-5 39278919 PMC11403874

[B147] Liu G. Philp A. M. Corte T. Travis M. A. Schilter H. Hansbro N. G. (2021). 'Therapeutic targets in lung tissue remodelling and fibrosis. Pharmacol. Ther. 225, 107839. 10.1016/j.pharmthera.2021.107839 33774068

[B148] Liu P. Luo G. Dodson M. Schmidlin C. J. Wei Y. Kerimoglu B. (2021). The NRF2-LOC344887 signaling axis suppresses pulmonary fibrosis. Redox Biol. 38, 101766. 10.1016/j.redox.2020.101766 33126057 PMC7573654

[B149] Liu R. Chen Y. Liu G. Li C. Song Y. Cao Z. (2020). 'PI3K/AKT pathway as a key link modulates the multidrug resistance of cancers. Cell Death Dis. 11, 797. 10.1038/s41419-020-02998-6 32973135 PMC7515865

[B150] Liu Y. Zhang Z. Yan L. Li X. Zhang J. Zhang X. (2020). Everolimus reduces postoperative arthrofibrosis in rabbits by inducing autophagy-mediated fibroblast apoptosis by PI3K/Akt/mTOR signaling pathway. Biochem. Biophys. Res. Commun. 533, 1–8. 10.1016/j.bbrc.2020.08.039 32919704

[B151] Liu Y. Li Y. Li J. Zuo X. Cao Q. Xie W. (2021). Inhibiting miR-1 attenuates pulmonary arterial hypertension in rats. Mol. Med. Rep. 23, 283. 10.3892/mmr.2021.11922 33604679 PMC7905329

[B152] Lou H. Wu L. Q. Wang H. Wei R. L. Cheng J. W. (2021). The potential role of osteopontin in the pathogenesis of graves' ophthalmopathy. Invest Ophthalmol. Vis. Sci. 62, 18. 10.1167/iovs.62.12.18 34546326 PMC8458783

[B153] Lu Y. Zhong W. Liu Y. Chen W. Zhang J. Zeng Z. (2022). 'Anti-PD-L1 antibody alleviates pulmonary fibrosis by inducing autophagy *via* inhibition of the PI3K/Akt/mTOR pathway. Int. Immunopharmacol. 104, 108504. 10.1016/j.intimp.2021.108504 35026657

[B154] Lukey P. T. Harrison S. A. Yang S. Man Y. Holman B. F. Rashidnasab A. (2019). A randomised, placebo-controlled study of omipalisib (PI3K/mTOR) in idiopathic pulmonary fibrosis. Eur. Respir. J. 53, 1801992. 10.1183/13993003.01992-2018 30765508

[B155] Lurje I. Gaisa N. T. Weiskirchen R. Tacke F. (2023). Mechanisms of organ fibrosis: emerging concepts and implications for novel treatment strategies. Mol. Asp. Med. 92, 101191. 10.1016/j.mam.2023.101191 37236017

[B156] Lv W. Liu S. Zhang Q. Hu W. Wu Y. Ren Y. (2021a). 'Circular RNA CircCOL5A1 sponges the MiR-7-5p/Epac1 axis to promote the progression of keloids through regulating PI3K/Akt signaling pathway. Front. Cell Dev. Biol. 9, 626027. 10.3389/fcell.2021.626027 33553184 PMC7859531

[B157] Lv W. Wu M. Ren Y. Luo X. Hu W. Zhang Q. (2021b). 'Treatment of keloids through Runx2 siRNA-induced inhibition of the PI3K/AKT signaling pathway. Mol. Med. Rep. 23, 55. 10.3892/mmr.2020.11693 33200804 PMC7706002

[B158] Lv D. Xu Z. Cheng P. Hu Z. Dong Y. Rong Y. (2023). 'S-Nitrosylation-mediated coupling of DJ-1 with PTEN induces PI3K/AKT/mTOR pathway-dependent keloid formation. Burns Trauma 11, tkad024. 10.1093/burnst/tkad024 38116467 PMC10729783

[B159] Ma Z. Yu R. Zhu Q. Sun L. Jian L. Wang X. (2020). CXCL16/CXCR6 axis promotes bleomycin-induced fibrotic process in MRC-5 cells *via* the PI3K/AKT/FOXO3a pathway. Int. Immunopharmacol. 81, 106035. 10.1016/j.intimp.2019.106035 31753588

[B160] Ma N. Wang Y. K. Xu S. Ni Q. Z. Zheng Q. W. Zhu B. (2021). PPDPF alleviates hepatic steatosis through inhibition of mTOR signaling. Nat. Commun. 12, 3059. 10.1038/s41467-021-23285-8 34031390 PMC8144412

[B161] Ma F. Shen J. Zhang H. Zhang Z. Yang A. Xiong J. (2022). A novel lncRNA FPASL regulates fibroblast proliferation *via* the PI3K/AKT and MAPK signaling pathways in hypertrophic scar. Acta Biochim. Biophys. Sin. (Shanghai) 55, 274–284. 10.3724/abbs.2022122 36082934 PMC10157618

[B162] Ma R. Huang X. Sun D. Wang J. Xue C. Ye Q. (2024). Tetrandrine alleviates silica-induced pulmonary fibrosis through PI3K/AKT pathway: network pharmacology investigation and experimental validation. Inflammation 47, 1109–1126. 10.1007/s10753-023-01964-6 38265677

[B163] Magaye R. R. Savira F. Hua Y. Xiong X. Huang L. Reid C. (2021). Attenuating PI3K/Akt-mTOR pathway reduces dihydrosphingosine 1 phosphate mediated collagen synthesis and hypertrophy in primary cardiac cells. Int. J. Biochem. Cell Biol. 134, 105952. 10.1016/j.biocel.2021.105952 33609744

[B164] Maity P. Singh K. Krug L. Koroma A. Hainzl A. Bloch W. (2021). Persistent JunB activation in fibroblasts disrupts stem cell niche interactions enforcing skin aging. Cell Rep. 36, 109634. 10.1016/j.celrep.2021.109634 34469740

[B165] Makena P. Kikalova T. Prasad G. L. Baxter S. A. (2023). Oxidative stress and lung fibrosis: towards an adverse outcome pathway. Int. J. Mol. Sci. 24, 12490. 10.3390/ijms241512490 37569865 PMC10419527

[B166] Malek E. Kim B.-G. Valent J. Driscoll J. Caimi P. Kim S.-J. (2018). Preclinical studies and a phase I trial of the TGF-β receptor inhibitor, vactosertib (TEW-7197), in combination with pomalidomide in patients with multiple myeloma refractory to bortezomib or lenalidomide. Blood 132, 1962. 10.1182/blood-2018-99-112449

[B167] Malek E. Hwang S. Lima M. de Caimi P. Gallogly M. M. Metheny L. III (2019). Preclinical studies and phase I trial of vactosertib in combination with pomalidomide in relapsed multiple myeloma: a corticosteroid-free approach by targeting TGF-β signaling pathway. Blood 134, 3232. 10.1182/blood-2019-126728

[B168] McMullen C. J. Chalmers S. Wood R. Cunningham M. R. Currie S. (2020). Sunitinib and imatinib display differential cardiotoxicity in adult rat cardiac fibroblasts that involves a role for calcium/calmodulin dependent protein kinase II. Front. Cardiovasc Med. 7, 630480. 10.3389/fcvm.2020.630480 33598481 PMC7882511

[B169] Melisi D. Garcia-Carbonero R. Macarulla T. Pezet D. Deplanque G. Fuchs M. (2018). 'Galunisertib plus gemcitabine vs. gemcitabine for first-line treatment of patients with unresectable pancreatic cancer. Br. J. Cancer 119, 1208–1214. 10.1038/s41416-018-0246-z 30318515 PMC6251034

[B170] Mempel T. R. Lill J. K. Altenburger L. M. (2024). 'How chemokines organize the tumour microenvironment. Nat. Rev. Cancer 24, 28–50. 10.1038/s41568-023-00635-w 38066335 PMC11480775

[B171] Mercer P. F. Woodcock H. V. Eley J. D. Platé M. Sulikowski M. G. Durrenberger P. F. (2016). Exploration of a potent PI3 kinase/mTOR inhibitor as a novel anti-fibrotic agent in IPF. Thorax 71, 701–711. 10.1136/thoraxjnl-2015-207429 27103349 PMC4975851

[B172] Merl-Pham J. Basak T. Knüppel L. Ramanujam D. Athanason M. Behr J. (2019). Quantitative proteomic profiling of extracellular matrix and site-specific collagen post-translational modifications in an *in vitro* model of lung fibrosis. Matrix Biol. Plus 1, 100005. 10.1016/j.mbplus.2019.04.002 33543004 PMC7852317

[B173] Metkar M. Pepin C. S. Moore M. J. (2024). 'Tailor made: the art of therapeutic mRNA design. Nat. Rev. Drug Discov. 23, 67–83. 10.1038/s41573-023-00827-x 38030688

[B174] Miao R. M. Sun X. F. Zhang Y. Y. Wu W. Fang Z. H. Zhao R. (2013). Clinical efficacy of tetrandrine combined with acetylcysteine effervescent tablets in treatment of silicosis. Zhonghua Lao Dong Wei Sheng Zhi Ye Bing Za Zhi 31, 857–858. 24370303

[B175] Mizushima N. Komatsu M. (2011). Autophagy: renovation of cells and tissues. Cell 147, 728–741. 10.1016/j.cell.2011.10.026 22078875

[B176] Mohammad S. I. Vasudevan A. Hussein Alzewmel A. Rab S. O. Ballal S. Kalia R. (2025). The mutual effects of stearoyl-CoA desaturase and cancer-associated fibroblasts: a focus on cancer biology. Exp. Cell Res. 447, 114508. 10.1016/j.yexcr.2025.114508 40122505

[B177] Mohyi M. Smith T. J. (2018). IGF1 receptor and thyroid-associated ophthalmopathy. J. Mol. Endocrinol. 61, T29–t43. 10.1530/JME-17-0276 29273685 PMC6561656

[B178] Molenaar J. C. (2003). From the library of the Netherlands Journal of Medicine. Rudolf Virchow: die Cellularpathologie in ihrer Begründung auf physiologische und pathologische Gewebelehre; 1858. Ned. Tijdschr. Geneeskd. 147, 2236–2244. 14640063

[B179] Mori Y. Dendl K. Cardinale J. Kratochwil C. Giesel F. L. Haberkorn U. (2023). FAPI PET: fibroblast activation protein inhibitor use in oncologic and nononcologic disease. Radiology 306, e220749. 10.1148/radiol.220749 36594838

[B180] Morshed S. A. Ma R. Latif R. Davies T. F. (2022). Mechanisms in graves eye disease: apoptosis as the end point of insulin-like growth factor 1 receptor inhibition. Thyroid 32, 429–439. 10.1089/thy.2021.0176 34927457 PMC9048181

[B181] Mulholland D. J. Dedhar S. Wu H. Nelson C. C. (2006). PTEN and GSK3beta: key regulators of progression to androgen-independent prostate cancer. Oncogene 25, 329–337. 10.1038/sj.onc.1209020 16421604

[B182] Muñoz-Espín D. Cañamero M. Maraver A. Gómez-López G. Contreras J. Murillo-Cuesta S. (2013). Programmed cell senescence during mammalian embryonic development. Cell 155, 1104–1118. 10.1016/j.cell.2013.10.019 24238962

[B369] Murata H. Zhou L. Ochoa S. Hasan A. Badiavas E. Falanga V. (1997). TGF-beta3 stimulates and regulates collagen synthesis through TGF-beta1-dependent and independent mechanisms. J. Invest. Dermatol 108, 258–262. 10.1111/1523-1747.ep12286451 9036921

[B183] Mutgan A. C. Besikcioglu H. E. Wang S. Friess H. Ceyhan G. O. Demir I. E. (2018). 'Insulin/IGF-driven cancer cell-stroma crosstalk as a novel therapeutic target in pancreatic cancer. Mol. Cancer 17, 66. 10.1186/s12943-018-0806-0 29475434 PMC5824531

[B184] Nathan C. Ding A. (2010). 'Nonresolving inflammation. Cell 140, 871–882. 10.1016/j.cell.2010.02.029 20303877

[B185] Nemeth K. Bayraktar R. Ferracin M. Calin G. A. (2024). Non-coding RNAs in disease: from mechanisms to therapeutics. Nat. Rev. Genet. 25, 211–232. 10.1038/s41576-023-00662-1 37968332

[B186] Newton K. Strasser A. Kayagaki N. Dixit V. M. (2024). 'Cell death. Cell 187, 235–256. 10.1016/j.cell.2023.11.044 38242081

[B187] Nishikai-Yan Shen T. Kanazawa S. Kado M. Okada K. Luo L. Hayashi A. (2017). Interleukin-6 stimulates Akt and p38 MAPK phosphorylation and fibroblast migration in non-diabetic but not diabetic mice. PLoS One 12, e0178232. 10.1371/journal.pone.0178232 28542434 PMC5441644

[B370] Niu W. Zhang Y. Liu H. Liang N. Xu L. Li Y. (2023). Single-cell profiling uncovers the roles of endometrial fibrosis and microenvironmental changes in adenomyosis. J. Inflamm. Res. 16, 1949–1965. 10.2147/jir.S402734 37179754 PMC10167994

[B188] Noordam R. Gunn D. A. Tomlin C. C. Maier A. B. Griffiths T. Catt S. D. (2013). 'Serum insulin-like growth factor 1 and facial ageing: high levels associate with reduced skin wrinkling in a cross-sectional study. Br. J. Dermatol 168, 533–538. 10.1111/bjd.12131 23363376

[B189] Noskovičová N. Petřek M. Eickelberg O. Heinzelmann K. (2015). Platelet-derived growth factor signaling in the lung. From lung development and disease to clinical studies. Am. J. Respir. Cell Mol. Biol. 52, 263–284. 10.1165/rcmb.2014-0294TR 25303647

[B190] Noto A. De Vitis C. Pisanu M. E. Roscilli G. Ricci G. Catizone A. (2017). Stearoyl-CoA-desaturase 1 regulates lung cancer stemness *via* stabilization and nuclear localization of YAP/TAZ. Oncogene 36, 4573–4584. 10.1038/onc.2017.212 28368399

[B191] Oakes S. A. Papa F. R. (2015). The role of endoplasmic reticulum stress in human pathology. Annu. Rev. Pathol. 10, 173–194. 10.1146/annurev-pathol-012513-104649 25387057 PMC5568783

[B192] Okada M. Oba Y. Yamawaki H. (2015). Endostatin stimulates proliferation and migration of adult rat cardiac fibroblasts through PI3K/Akt pathway. Eur. J. Pharmacol. 750, 20–26. 10.1016/j.ejphar.2015.01.019 25620135

[B193] Oki Y. Kelly K. R. Flinn I. Patel M. R. Gharavi R. Ma A. (2017). CUDC-907 in relapsed/refractory diffuse large B-cell lymphoma, including patients with MYC-alterations: results from an expanded phase I trial. Haematologica 102, 1923–1930. 10.3324/haematol.2017.172882 28860342 PMC5664396

[B194] Ornatowski W. Lu Q. Yegambaram M. Garcia A. E. Zemskov E. A. Maltepe E. (2020). Complex interplay between autophagy and oxidative stress in the development of pulmonary disease. Redox Biol. 36, 101679. 10.1016/j.redox.2020.101679 32818797 PMC7451718

[B371] Oouchi Y. Watanabe M. Ida Y. Ohguro H. Hikage F. (2021). Rosiglitasone and ROCK inhibitors modulate fibrogenetic changes in TGF-β2 treated human conjunctival fibroblasts (HconF) in different manners. Int. J. Mol. Sci. 22, 7335. 10.3390/ijms22147335 34298955 PMC8307967

[B195] Oza A. Kaye S. Van Tornout J. Sessa C. Gore M. Naumann R. W. (2018). Phase 2 study evaluating intermittent and continuous linsitinib and weekly paclitaxel in patients with recurrent platinum resistant ovarian epithelial cancer. Gynecol. Oncol. 149, 275–282. 10.1016/j.ygyno.2018.01.019 29454514

[B196] Pan D. Schellhardt L. Acevedo-Cintron J. A. Hunter D. Snyder-Warwick A. K. Mackinnon S. E. (2022). IL-4 expressing cells are recruited to nerve after injury and promote regeneration. Exp. Neurol. 347, 113909. 10.1016/j.expneurol.2021.113909 34717939 PMC8887027

[B197] Pan K. Li Q. Guo Z. Li Z. (2025). Healing action of Interleukin-4 (IL-4) in acute and chronic inflammatory conditions: mechanisms and therapeutic strategies. Pharmacol. Ther. 265, 108760. 10.1016/j.pharmthera.2024.108760 39615600

[B198] Park S. Ahn J. Y. Lim M. J. Kim M. H. Yun Y. S. Jeong G. (2010). Sustained expression of NADPH oxidase 4 by p38 MAPK-akt signaling potentiates radiation-induced differentiation of lung fibroblasts. J. Mol. Med. Berl. 88, 807–816. 10.1007/s00109-010-0622-5 20396861

[B199] Park J. H. Shin J. M. Yang H. W. Kim T. H. Lee S. H. Lee H. M. (2020). Cigarette smoke extract stimulates MMP-2 production in nasal fibroblasts *via* ROS/PI3K, akt, and NF-κB signaling pathways. Antioxidants (Basel) 9, 739. 10.3390/antiox9080739 32806646 PMC7465436

[B200] Pashaei M. Farhadi E. Kavosi H. Madreseh E. Enayati S. Mahmoudi M. (2024). 'Talabostat, fibroblast activation protein inhibitor, attenuates inflammation and fibrosis in systemic sclerosis. Inflammopharmacology 32, 3181–3193. 10.1007/s10787-024-01536-6 39167314

[B201] Patton J. S. Byron P. R. (2007). Inhaling medicines: delivering drugs to the body through the lungs. Nat. Rev. Drug Discov. 6, 67–74. 10.1038/nrd2153 17195033

[B202] Patton J. S. Fishburn C. S. Weers J. G. (2004). The lungs as a portal of entry for systemic drug delivery. Proc. Am. Thorac. Soc. 1, 338–344. 10.1513/pats.200409-049TA 16113455

[B203] Pei X. Zheng F. Li Y. Lin Z. Han X. Feng Y. (2022). Niclosamide ethanolamine salt alleviates idiopathic pulmonary fibrosis by modulating the PI3K-mTORC1 pathway. Cells 11, 346. 10.3390/cells11030346 35159160 PMC8834116

[B204] Peng D. Fu M. Wang M. Wei Y. Wei X. (2022). Targeting TGF-β signal transduction for fibrosis and cancer therapy. Mol. Cancer 21, 104. 10.1186/s12943-022-01569-x 35461253 PMC9033932

[B205] Peng J. Xiao X. Li S. Lyu X. Gong H. Tan S. (2023). Aspirin alleviates pulmonary fibrosis through PI3K/AKT/mTOR-mediated autophagy pathway. Exp. Gerontol. 172, 112085. 10.1016/j.exger.2023.112085 36623738

[B206] Peng H. Zhang Y. Min J. Tan Y. Liu S. (2024). 'Loss of ZNF451 mediates fibroblast activation and promotes lung fibrosis. Respir. Res. 25, 160. 10.1186/s12931-024-02781-7 38600524 PMC11008011

[B207] Peterson J. M. Jay J. W. Wang Y. Joglar A. A. Prasai A. Palackic A. (2022). Galunisertib exerts antifibrotic effects on TGF-β-Induced fibroproliferative dermal fibroblasts. Int. J. Mol. Sci. 23, 6689. 10.3390/ijms23126689 35743131 PMC9223605

[B208] Philip P. A. Goldman B. Ramanathan R. K. Lenz H. J. Lowy A. M. Whitehead R. P. (2014). 'Dual blockade of epidermal growth factor receptor and insulin-like growth factor receptor-1 signaling in metastatic pancreatic cancer: phase Ib and randomized phase II trial of gemcitabine, erlotinib, and cixutumumab *versus* gemcitabine plus erlotinib (SWOG S0727). Cancer 120, 2980–2985. 10.1002/cncr.28744 25041791 PMC4284963

[B209] Philippou A. Christopoulos P. F. Koutsilieris D. M. (2017). Clinical studies in humans targeting the various components of the IGF system show lack of efficacy in the treatment of cancer. Mutat. Res. Rev. Mutat. Res. 772, 105–122. 10.1016/j.mrrev.2016.09.005 28528684

[B210] Piera-Velazquez S. Jimenez S. A. (2021). Oxidative stress induced by reactive oxygen species (ROS) and NADPH oxidase 4 (NOX4) in the pathogenesis of the fibrotic process in systemic sclerosis: a promising therapeutic target. J. Clin. Med. 10, 4791. 10.3390/jcm10204791 34682914 PMC8539594

[B211] Plikus M. V. Wang X. Sinha S. Forte E. Thompson S. M. Herzog E. L. (2021). Fibroblasts: origins, definitions, and functions in health and disease. Cell 184, 3852–3872. 10.1016/j.cell.2021.06.024 34297930 PMC8566693

[B212] Porter A. G. Jänicke R. U. (1999). 'Emerging roles of caspase-3 in apoptosis. Cell Death Differ. 6, 99–104. 10.1038/sj.cdd.4400476 10200555

[B213] Povsic T. J. Kohout T. A. Lefkowitz R. J. (2003). 'Beta-arrestin1 mediates insulin-like growth factor 1 (IGF-1) activation of phosphatidylinositol 3-kinase (PI3K) and anti-apoptosis. J. Biol. Chem. 278, 51334–51339. 10.1074/jbc.M309968200 14534298

[B214] Prossnitz E. R. Barton M. (2023). The G protein-coupled oestrogen receptor GPER in health and disease: an update. Nat. Rev. Endocrinol. 19, 407–424. 10.1038/s41574-023-00822-7 37193881 PMC10187525

[B215] Qian C. Lai C. J. Bao R. Wang D. G. Wang J. Xu G. X. (2012). 'Cancer network disruption by a single molecule inhibitor targeting both histone deacetylase activity and phosphatidylinositol 3-kinase signaling. Clin. Cancer Res. 18, 4104–4113. 10.1158/1078-0432.CCR-12-0055 22693356

[B216] Qin Y. Ge G. Yang P. Wang L. Qiao Y. Pan G. (2023). An update on adipose-derived stem cells for regenerative medicine: where challenge meets opportunity. Adv. Sci. (Weinh) 10, e2207334. 10.1002/advs.202207334 37162248 PMC10369252

[B217] Qiu Y. Wang Z. Zhang X. Huang P. Zhang W. Zhang K. (2020). A long-acting isomer of Ac-SDKP attenuates pulmonary fibrosis through SRPK1-mediated PI3K/AKT and Smad2 pathway inhibition. IUBMB Life 72, 2611–2626. 10.1002/iub.2389 33135306

[B218] Ramani K. Biswas P. S. (2019). Interleukin-17: friend or foe in organ fibrosis. Cytokine 120, 282–288. 10.1016/j.cyto.2018.11.003 30772195 PMC6555688

[B219] Ray S. Bhattacharyya S. Panda P. Pandey A. Ghosal K. (2020). “Advances in pulmonary nanomedicine for therapeutic management of respiratory diseases,” in Nano medicine and nano safety: recent trends and clinical evidences (Springer).

[B220] Reddy V. S. Harskamp R. E. van Ginkel M. W. Calhoon J. Baisden C. E. Kim I. S. (2008). Interleukin-18 stimulates fibronectin expression in primary human cardiac fibroblasts *via* PI3K-Akt-dependent NF-kappaB activation. J. Cell Physiol. 215, 697–707. 10.1002/jcp.21348 18064631

[B221] Riccetti M. R. Green J. Taylor T. J. Perl A. T. (2024). Prenatal FGFR2 signaling *via* PI3K/AKT specifies the PDGFRA(+) myofibroblast. Am. J. Respir. Cell Mol. Biol. 70, 63–77. 10.1165/rcmb.2023-0245OC 37734036 PMC10768833

[B222] Rimal R. Desai P. Daware R. Hosseinnejad A. Prakash J. Lammers T. (2022). 'Cancer-associated fibroblasts: origin, function, imaging, and therapeutic targeting. Adv. Drug Deliv. Rev. 189, 114504. 10.1016/j.addr.2022.114504 35998825

[B223] Ring A. Kilburn L. S. Pearson A. Moretti L. Afshari-Mehr A. Wardley A. M. (2023). Olaparib and Ceralasertib (AZD6738) in patients with triple-negative advanced breast cancer: results from cohort E of the plasmaMATCH trial (CRUK/15/010). Clin. Cancer Res. 29, 4751–4759. 10.1158/1078-0432.CCR-23-1696 37773077 PMC10690092

[B224] Rinkevich Y. Walmsley G. G. Hu M. S. Maan Z. N. Newman A. M. Drukker M. (2015). 'Skin fibrosis. Identification and isolation of a dermal lineage with intrinsic fibrogenic potential. Science 348, aaa2151. 10.1126/science.aaa2151 25883361 PMC5088503

[B225] Run L. Tian Z. Xu L. Du J. Li N. Wang Q. (2023). 'Knockdown of IL4I1 improved high glucose-evoked insulin resistance in HepG2 cells by alleviating inflammation and lipotoxicity through AHR activation. Appl. Biochem. Biotechnol. 195, 6694–6707. 10.1007/s12010-023-04399-9 36913096

[B226] Rurik J. G. Tombácz I. Yadegari A. Méndez Fernández P. O. Shewale S. V. Li L. (2022). 'CAR T cells produced *in vivo* to treat cardiac injury. Science 375, 91–96. 10.1126/science.abm0594 34990237 PMC9983611

[B227] Santini V. Valcárcel D. Platzbecker U. Komrokji R. S. Cleverly A. L. Lahn M. M. (2019). Phase II study of the ALK5 inhibitor galunisertib in very low-low-and intermediate-risk myelodysplastic syndromes. Clin. Cancer Res. 25, 6976–6985. 10.1158/1078-0432.CCR-19-1338 31481511

[B228] Sappino A. P. Masouyé I. Saurat J. H. Gabbiani G. (1990). Smooth muscle differentiation in scleroderma fibroblastic cells. Am. J. Pathol. 137, 585–591. 1698026 PMC1877526

[B229] Saraswathibhatla A. Indana D. Chaudhuri O. (2023). 'Cell-extracellular matrix mechanotransduction in 3D. Nat. Rev. Mol. Cell Biol. 24, 495–516. 10.1038/s41580-023-00583-1 36849594 PMC10656994

[B230] Sawant M. Hinz B. Schönborn K. Zeinert I. Eckes B. Krieg T. (2021). A story of fibers and stress: matrix-embedded signals for fibroblast activation in the skin. Wound Repair Regen. 29, 515–530. 10.1111/wrr.12950 34081361

[B231] Sazonova O. V. Blishchenko E. Y. Tolmazova A. G. Khachin D. P. Leontiev K. V. Karelin A. A. (2007). 'Stimulation of fibroblast proliferation by neokyotorphin requires ca influx and activation of PKA, CaMK II and MAPK/ERK. Febs J. 274, 474–484. 10.1111/j.1742-4658.2006.05594.x 17229152

[B232] Schafer S. Viswanathan S. Widjaja A. A. Lim W. W. Moreno-Moral A. DeLaughter D. M. (2017). 'IL-11 is a crucial determinant of cardiovascular fibrosis. Nature 552, 110–115. 10.1038/nature24676 29160304 PMC5807082

[B233] Schlessinger J. (2000). Cell signaling by receptor tyrosine kinases. Cell 103, 211–225. 10.1016/s0092-8674(00)00114-8 11057895

[B234] Scott A. M. Wiseman G. Welt S. Adjei A. Lee F. T. Hopkins W. (2003). A Phase I dose-escalation study of sibrotuzumab in patients with advanced or metastatic fibroblast activation protein-positive cancer. Clin. Cancer Res. 9, 1639–1647. Available online at: https://aacrjournals.org/clincancerres/article/9/5/1639/204681/A-Phase-I-Dose-Escalation-Study-of-Sibrotuzumab-in. 12738716

[B235] Seo J. Lee C. Hwang H. S. Kim B. Thao le Q. Lee E. S. (2016). Therapeutic advantage of inhaled tacrolimus-bound albumin nanoparticles in a bleomycin-induced pulmonary fibrosis mouse model. Pulm. Pharmacol. Ther. 36, 53–61. 10.1016/j.pupt.2016.01.001 26768967

[B236] Serpico L. Dello Iacono S. Cammarano A. De Stefano L. (2023). Recent advances in stimuli-responsive hydrogel-based wound dressing. Gels 9, 451. 10.3390/gels9060451 37367122 PMC10298082

[B237] Shahabadi N. Moshiri M. Roohbakhsh A. Imenshahidi M. Hashemi M. Amin F. (2022). A dose-related positive effect of inhaled simvastatin-loaded PLGA nanoparticles on paraquat-induced pulmonary fibrosis in rats. Basic Clin. Pharmacol. Toxicol. 131, 251–261. 10.1111/bcpt.13771 35802512

[B238] Shahvali S. Rahiman N. Jaafari M. R. Arabi L. (2023). Targeting fibroblast activation protein (FAP): advances in CAR-T cell, antibody, and vaccine in cancer immunotherapy. Drug Deliv. Transl. Res. 13, 2041–2056. 10.1007/s13346-023-01308-9 36840906

[B239] Shang S. Li X. Wang H. Zhou Y. Pang K. Li P. (2024). Targeted therapy of kidney disease with nanoparticle drug delivery materials. Bioact. Mater 37, 206–221. 10.1016/j.bioactmat.2024.03.014 38560369 PMC10979125

[B240] Shao D. D. Suresh R. Vakil V. Gomer R. H. Pilling D. (2008). Pivotal advance: th-1 cytokines inhibit, and Th-2 cytokines promote fibrocyte differentiation. J. Leukoc. Biol. 83, 1323–1333. 10.1189/jlb.1107782 18332234 PMC2659591

[B241] Shao D. Liu X. Wu J. Zhang A. Bai Y. Zhao P. (2022). Identification of the active compounds and functional mechanisms of jinshui huanxian formula in pulmonary fibrosis by integrating serum pharmacochemistry with network. Phytomedicine 102, 154177. 10.1016/j.phymed.2022.154177 35636171

[B242] Sharma M. Chuang W. W. Sun Z. (2002). Phosphatidylinositol 3-kinase/Akt stimulates androgen pathway through GSK3beta inhibition and nuclear beta-catenin accumulation. J. Biol. Chem. 277, 30935–30941. 10.1074/jbc.M201919200 12063252

[B243] Shi J. Li J. Guan H. Cai W. Bai X. Fang X. (2014). 'Anti-fibrotic actions of interleukin-10 against hypertrophic scarring by activation of PI3K/AKT and STAT3 signaling pathways in scar-forming fibroblasts. PLoS One 9, e98228. 10.1371/journal.pone.0098228 24878845 PMC4039501

[B244] Shi W. Wu Y. Bian D. (2021). p75NTR silencing inhibits proliferation, migration, and extracellular matrix deposition of hypertrophic scar fibroblasts by activating autophagy through inhibiting the PI3K/Akt/mTOR pathway. Can. J. Physiol. Pharmacol. 99, 349–359. 10.1139/cjpp-2020-0219 32726570

[B245] Shi L. X. Liu X. R. Zhou L. Y. Zhu Z. Q. Yuan Q. Zou T. (2023). 'Nanocarriers for gene delivery to the cardiovascular system. Biomater. Sci. 11, 7709–7729. 10.1039/d3bm01275a 37877418

[B246] Shi N. Wang Y. Xia Z. Zhang J. Jia S. Jiao Y. (2024). The regulatory role of the apelin/APJ axis in scarring: identification of upstream and downstream mechanisms. Biochim. Biophys. Acta Mol. Basis Dis. 1870, 167125. 10.1016/j.bbadis.2024.167125 38508477

[B247] Shi W. Q. Li T. Liang R. Li B. Zhou X. (2024). Targeting scleral remodeling and myopia development in form deprivation myopia through inhibition of EFEMP1 expression. Biochim. Biophys. Acta Mol. Basis Dis. 1870, 166981. 10.1016/j.bbadis.2023.166981 38101653

[B248] Shiraishi M. Yamaguchi A. Suzuki K. (2022). Nrg1/ErbB signaling-mediated regulation of fibrosis after myocardial infarction. Faseb J. 36, e22150. 10.1096/fj.202101428RR 34997943

[B249] Simm A. Nestler M. Hoppe V. (1998). Mitogenic effect of PDGF-AA on cardiac fibroblasts. Basic Res. Cardiol. 93 (Suppl. 3), 40–43. 10.1007/s003950050209 9879443

[B250] Singh K. Maity P. Krug L. Meyer P. Treiber N. Lucas T. (2015). Superoxide anion radicals induce IGF-1 resistance through concomitant activation of PTP1B and PTEN. EMBO Mol. Med. 7, 59–77. 10.15252/emmm.201404082 25520316 PMC4309668

[B251] Sinha N. R. Hofmann A. C. Suleiman L. A. Laub R. Tripathi R. Chaurasia S. S. (2025). PI3K signaling and lysyl oxidase is critical to corneal stroma fibrosis following mustard gas injury. Exp. Eye Res. 251, 110213. 10.1016/j.exer.2024.110213 39706242 PMC11798705

[B252] Slowikowski K. Nguyen H. N. Noss E. H. Simmons D. P. Mizoguchi F. Watts G. F. M. (2020). CUX1 and IκBζ (NFKBIZ) mediate the synergistic inflammatory response to TNF and IL-17A in stromal fibroblasts. Proc. Natl. Acad. Sci. U. S. A. 117, 5532–5541. 10.1073/pnas.1912702117 32079724 PMC7071902

[B253] Smith R. E. Strieter R. M. Zhang K. Phan S. H. Standiford T. J. Lukacs N. W. (1995). A role for C-C chemokines in fibrotic lung disease. J. Leukoc. Biol. 57, 782–787. 10.1002/jlb.57.5.782 7539030

[B254] Smith T. J. Kahaly G. J. Ezra D. G. Fleming J. C. Dailey R. A. Tang R. A. (2017). 'Teprotumumab for thyroid-associated ophthalmopathy. N. Engl. J. Med. 376, 1748–1761. 10.1056/NEJMoa1614949 28467880 PMC5718164

[B255] Smith E. R. Wigg B. Holt S. Hewitson T. D. (2019). TGF-β1 modifies histone acetylation and acetyl-coenzyme A metabolism in renal myofibroblasts. Am. J. Physiol. Ren. Physiol. 316, F517–F529. 10.1152/ajprenal.00513.2018 30623724

[B256] Sun X. Kim Y. H. Phan T. N. Yang B. S. (2014). Topical application of ALK5 inhibitor A-83-01 reduces burn wound contraction in rats by suppressing myofibroblast population. Biosci. Biotechnol. Biochem. 78, 1805–1812. 10.1080/09168451.2014.932666 25351330

[B257] Sun H. N. Ren C. X. Lee D. H. Wang W. H. Guo X. Y. Hao Y. Y. (2023). PRDX1 negatively regulates bleomycin-induced pulmonary fibrosis *via* inhibiting the epithelial-mesenchymal transition and lung fibroblast proliferation *in vitro* and *in vivo* . Cell Mol. Biol. Lett. 28, 48. 10.1186/s11658-023-00460-x 37268886 PMC10236698

[B258] Sun W. Lei Y. Jiang Z. Wang K. Liu H. Xu T. (2025). BPA and low-Se exacerbate apoptosis and mitophagy in chicken pancreatic cells by regulating the PTEN/PI3K/AKT/mTOR pathway. J. Adv. Res. 67, 61–69. 10.1016/j.jare.2024.01.029 38311007 PMC11725106

[B259] Sun F. Bi Q. Wang X. Liu J. (2020). 'Down-regulation of mir-27b promotes angiogenesis and fibroblast activation through activating PI3K/AKT signaling pathway. Wound Repair Regen. 28, 39–48. 10.1111/wrr.12765 31587435

[B260] Sun K. Luo J. Guo J. Yao X. Jing X. Guo F. (2020). The PI3K/AKT/mTOR signaling pathway in osteoarthritis: a narrative review. Osteoarthr. Cartil. 28, 400–409. 10.1016/j.joca.2020.02.027 32081707

[B261] Talbott H. E. Mascharak S. Griffin M. Wan D. C. Longaker M. T. (2022). 'Wound healing, fibroblast heterogeneity, and fibrosis. Cell Stem Cell 29, 1161–1180. 10.1016/j.stem.2022.07.006 35931028 PMC9357250

[B262] Tang Y. Huo X. Liu J. Tang Y. Zhang M. Xie W. (2022). 'MicroRNA-325-3p targets human epididymis protein 4 to relieve right ventricular fibrosis in rats with pulmonary arterial hypertension. Cardiovasc Ther. 2022, 4382999. 10.1155/2022/4382999 35136419 PMC8800631

[B263] Taylor C. T. Scholz C. C. (2022). The effect of HIF on metabolism and immunity. Nat. Rev. Nephrol. 18, 573–587. 10.1038/s41581-022-00587-8 35726016 PMC9208707

[B264] Telo S. Farolfi A. Castellucci P. Antonacci F. Solli P. Mosconi C. (2022). A case of [(68)Ga]Ga-FAPI-46-avid and [(18)F]F-FDG-negative COVID-19 pneumonia sequelae. Eur. J. Nucl Med. Mol. Imaging 49, 2452–2453. 10.1007/s00259-022-05720-0 35179626 PMC8854481

[B265] Theocharis A. D. Skandalis S. S. Gialeli C. Karamanos N. K. (2016). 'Extracellular matrix structure. Adv. Drug Deliv. Rev. 97, 4–27. 10.1016/j.addr.2015.11.001 26562801

[B266] Todd N. W. Luzina I. G. Atamas S. P. (2012). 'Molecular and cellular mechanisms of pulmonary fibrosis. Fibrogenes. Tissue Repair 5, 11. 10.1186/1755-1536-5-11 22824096 PMC3443459

[B267] Tofovic S. P. Zhang X. Jackson E. K. Zhu H. Petrusevska G. (2009). 2-methoxyestradiol attenuates bleomycin-induced pulmonary hypertension and fibrosis in estrogen-deficient rats. Vasc. Pharmacol. 51, 190–197. 10.1016/j.vph.2009.06.002 19540933 PMC2760300

[B268] Travaglini K. J. Nabhan A. N. Penland L. Sinha R. Gillich A. Sit R. V. (2020). A molecular cell atlas of the human lung from single-cell RNA sequencing. Nature 587, 619–625. 10.1038/s41586-020-2922-4 33208946 PMC7704697

[B269] Travers J. G. Kamal F. A. Robbins J. Yutzey K. E. Blaxall B. C. (2016). Cardiac fibrosis: the fibroblast awakens. Circ. Res. 118, 1021–1040. 10.1161/CIRCRESAHA.115.306565 26987915 PMC4800485

[B270] Tschumperlin D. J. Ligresti G. Hilscher M. B. Shah V. H. (2018). 'Mechanosensing and fibrosis. J. Clin. Invest 128, 74–84. 10.1172/JCI93561 29293092 PMC5749510

[B271] Tsoyi K. Liang X. De Rossi G. Ryter S. W. Xiong K. Chu S. G. (2021). CD148 deficiency in fibroblasts promotes the development of pulmonary fibrosis. Am. J. Respir. Crit. Care Med. 204, 312–325. 10.1164/rccm.202008-3100OC 33784491 PMC8513593

[B372] Umetsu A. Ida Y. Sato T. Higashide M. Nishikiori N. Furuhashi M. (2024). RHO-associated coiled-coil-containing protein kinase inhibitors significantly modulate the epithelial-mesenchymal transition induced by TGF-β2 in the 2-D and 3-D cultures of human corneal stroma fibroblasts. Biomedicines 12, 2784. 10.3390/biomedicines12122784 39767691 PMC11673340

[B272] van Rijt S. H. Bein T. Meiners S. (2014). 'Medical nanoparticles for next generation drug delivery to the lungs. Eur. Respir. J. 44, 765–774. 10.1183/09031936.00212813 24791828

[B273] Vo T. T. T. Wee Y. Chen Y. L. Cheng H. C. Tuan V. P. Lee I. T. (2021). 'Surfactin attenuates particulate matter-induced COX-2-dependent PGE(2) production in human gingival fibroblasts by inhibiting TLR2 and TLR4/MyD88/NADPH oxidase/ROS/PI3K/Akt/NF-κB signaling pathway. J. Periodontal Res. 56, 1185–1199. 10.1111/jre.12932 34486757

[B274] von Mehren M. George S. Heinrich M. C. Schuetze S. M. Yap J. T. Yu J. Q. (2020). 'Linsitinib (OSI-906) for the treatment of adult and pediatric wild-type gastrointestinal stromal tumors, a SARC phase II study. Clin. Cancer Res. 26, 1837–1845. 10.1158/1078-0432.CCR-19-1069 31792037 PMC7856429

[B275] Wang H. Tibbitt M. W. Langer S. J. Leinwand L. A. Anseth K. S. (2013). Hydrogels preserve native phenotypes of valvular fibroblasts through an elasticity-regulated PI3K/AKT pathway. Proc. Natl. Acad. Sci. U. S. A. 110, 19336–19341. 10.1073/pnas.1306369110 24218588 PMC3845151

[B276] Wang C. Liu E. Li W. Cui J. Li T. (2018). MiR-3188 inhibits non-small cell lung cancer cell proliferation through FOXO1-Mediated mTOR-p-PI3K/AKT-c-JUN signaling pathway. Front. Pharmacol. 9, 1362. 10.3389/fphar.2018.01362 30618730 PMC6297856

[B277] Wang J. Wu H. Zhao Y. Qin Y. Zhang Y. Pang H. (2021a). Extracellular vesicles from HIF-1α-Overexpressing adipose-derived stem cells restore diabetic wounds through accelerated fibroblast proliferation and migration. Int. J. Nanomedicine 16, 7943–7957. 10.2147/IJN.S335438 34887659 PMC8652947

[B278] Wang J. You J. Gong D. Xu Y. Yang B. Jiang C. (2021b). PDGF-BB induces conversion, proliferation, migration, and collagen synthesis of oral mucosal fibroblasts through PDGFR-β/PI3K/AKT signaling pathway. Cancer Biomark. 30, 407–415. 10.3233/CBM-201681 33492283 PMC12499994

[B279] Wang L. Li S. Yao Y. Yin W. Ye T. (2021c). The role of natural products in the prevention and treatment of pulmonary fibrosis: a review. Food Funct. 12, 990–1007. 10.1039/d0fo03001e 33459740

[B280] Wang Q. Wang P. Qin Z. Yang X. Pan B. Nie F. (2021d). Altered glucose metabolism and cell function in keloid fibroblasts under hypoxia. Redox Biol. 38, 101815. 10.1016/j.redox.2020.101815 33278780 PMC7718484

[B281] Wang F. T. Wu T. Q. Lin Y. Jiao Y. R. Li J. Y. Ruan Y. (2024). The role of the CXCR6/CXCL16 axis in the pathogenesis of fibrotic disease. Int. Immunopharmacol. 132, 112015. 10.1016/j.intimp.2024.112015 38608478

[B282] Wang H. Wu Q. Liu Z. Luo X. Fan Y. Liu Y. (2014). Downregulation of FAP suppresses cell proliferation and metastasis through PTEN/PI3K/AKT and Ras-ERK signaling in oral squamous cell carcinoma. Cell Death Dis. 5, e1155. 10.1038/cddis.2014.122 24722280 PMC5424105

[B283] Wang J. Hu K. Cai X. Yang B. He Q. Wang J. (2022). Targeting PI3K/AKT signaling for treatment of idiopathic pulmonary fibrosis. Acta Pharm. Sin. B 12, 18–32. 10.1016/j.apsb.2021.07.023 35127370 PMC8799876

[B284] Wang J. Guo R. Ma X. Wang Y. Zhang Q. Zheng N. (2023). Liraglutide inhibits AngII-induced cardiac fibroblast proliferation and ECM deposition through regulating miR-21/PTEN/PI3K pathway. Cell Tissue Bank. 24, 125–137. 10.1007/s10561-022-10021-9 35792987

[B285] Wang J. Du H. Xie W. Bi J. Zhang H. Liu X. (2024). 'CAR-macrophage therapy alleviates myocardial ischemia-reperfusion injury. Circ. Res. 135, 1161–1174. 10.1161/CIRCRESAHA.124.325212 39465245

[B286] Wang J. H. Su F. Wang S. Lu X. C. Zhang S. H. Chen D. (2014). CXCR6 deficiency attenuates pressure overload-induced monocytes migration and cardiac fibrosis through downregulating TNF-α-dependent MMP9 pathway. Int. J. Clin. Exp. Pathol. 7, 6514–6523. 25400729 PMC4230124

[B287] Wang L. Cheng J. Huang J. Xiao T. Tang Z. (2024). The mechanism of IL-13 targeting IL-13Rα2 in regulating oral mucosal FBs through PI3K/AKT/mTOR. Oral Dis. 30, 3142–3154. 10.1111/odi.14760 37897109

[B288] Wang R. Jia J. Zhou L. Zhu X. Tang Z. Shen H. (2024). 'miR-758-3p/ILK signaling modulated angiogenesis by regulating VEGFA in wound healing. Int. J. Med. Sci. 21, 175–187. 10.7150/ijms.86733 38164357 PMC10750343

[B289] Wang S. Huang Z. Liu Y. Sun H. Zhou Y. Shi J. (2023). Sustainably released nanoparticle-based rhynchophylline limits pulmonary fibrosis by inhibiting the TEK-PI3K/AKT signaling pathway. Transl. Lung Cancer Res. 12, 427–445. 10.21037/tlcr-22-675 37057119 PMC10087999

[B290] Wang X. Li W. Zhang Y. Sun Q. Cao J. Tan N. (2022). 'Calycosin as a novel PI3K activator reduces inflammation and fibrosis in heart failure through AKT-IKK/STAT3 axis. Front. Pharmacol. 13, 828061. 10.3389/fphar.2022.828061 35264961 PMC8899514

[B291] Wang Y. Ma Z. Peng W. Yu Q. Liang W. Cao L. (2025). '3,5,6,7,8,3',4'- Heptamethoxyflavonoid inhibits TGF-β1-induced epithelial-mesenchymal transition by regulating oxidative stress and autophagy through MEK/ERK/PI3K/AKT/mTOR signaling pathway. Sci. Rep. 15, 4567. 10.1038/s41598-025-88869-6 39915543 PMC11802913

[B292] Wang Z. Li X. Liu X. Yang Y. Yan Y. Cui D. (2025). 'Mechanistic insights into the anti-fibrotic effects of estrogen *via* the PI3K-Akt pathway in frozen shoulder. J. Steroid Biochem. Mol. Biol. 249, 106701. 10.1016/j.jsbmb.2025.106701 39947440

[B293] Wei Y. Kim T. J. Peng D. H. Duan D. Gibbons D. L. Yamauchi M. (2017). Fibroblast-specific inhibition of TGF-β1 signaling attenuates lung and tumor fibrosis. J. Clin. Invest 127, 3675–3688. 10.1172/JCI94624 28872461 PMC5617667

[B294] Wei K. Nguyen H. N. Brenner M. B. (2021). 'Fibroblast pathology in inflammatory diseases. J. Clin. Invest 131, e149538. 10.1172/JCI149538 34651581 PMC8516469

[B295] Wei J. Peng M. Y. Wang S. N. Lu H. X. (2024). CXCL4:NLRP3-mediated pyroptosis product that regulates cardiac fibrosis. Int. Immunopharmacol. 133, 112096. 10.1016/j.intimp.2024.112096 38657496

[B296] Wei F. Wang A. Wang Q. Han W. Rong R. Wang L. (2020). 'Plasma endothelial cells-derived extracellular vesicles promote wound healing in diabetes through YAP and the PI3K/Akt/mTOR pathway. Aging (Albany NY) 12, 12002–12018. 10.18632/aging.103366 32570219 PMC7343472

[B297] Wei P. Zhong C. Yang X. Shu F. Xiao S. Gong T. (2020). 'Exosomes derived from human amniotic epithelial cells accelerate diabetic wound healing *via* PI3K-AKT-mTOR-mediated promotion in angiogenesis and fibroblast function. Burns Trauma 8, tkaa020. 10.1093/burnst/tkaa020 32923490 PMC7476545

[B298] Witherel C. E. Abebayehu D. Barker T. H. Spiller K. L. (2019). Macrophage and fibroblast interactions in biomaterial-mediated fibrosis. Adv. Healthc. Mater 8, e1801451. 10.1002/adhm.201801451 30658015 PMC6415913

[B299] Wlaschek M. Maity P. Makrantonaki E. Scharffetter-Kochanek K. (2021). 'Connective tissue and fibroblast senescence in skin aging. J. Invest Dermatol 141, 985–992. 10.1016/j.jid.2020.11.010 33563466

[B300] Wong R. S. (2011). 'Apoptosis in cancer: from pathogenesis to treatment. J. Exp. Clin. Cancer Res. 30, 87. 10.1186/1756-9966-30-87 21943236 PMC3197541

[B301] Woodcock H. V. Eley J. D. Guillotin D. Platé M. Nanthakumar C. B. Martufi M. (2019). The mTORC1/4E-BP1 axis represents a critical signaling node during fibrogenesis. Nat. Commun. 10, 6. 10.1038/s41467-018-07858-8 30602778 PMC6315032

[B302] Workman P. Clarke P. A. Raynaud F. I. van Montfort R. L. (2010). Drugging the PI3 kinome: from chemical tools to drugs in the clinic. Cancer Res. 70, 2146–2157. 10.1158/0008-5472.CAN-09-4355 20179189 PMC3242038

[B303] Wu X. Li J. Yang X. Bai X. Shi J. Gao J. (2018). miR-155 inhibits the formation of hypertrophic scar fibroblasts by targeting HIF-1α *via* PI3K/AKT pathway. J. Mol. Histol. 49, 377–387. 10.1007/s10735-018-9778-z 29785488

[B304] Wu D. Kang L. Tian J. Wu Y. Liu J. Li Z. (2020). 'Exosomes derived from bone mesenchymal stem cells with the stimulation of Fe(3)O(4) nanoparticles and static magnetic field enhance wound healing through upregulated miR-21-5p. Int. J. Nanomedicine 15, 7979–7993. 10.2147/IJN.S275650 33116513 PMC7585514

[B305] Wu B. Tang X. Zhou Z. Ke H. Tang S. Ke R. (2021). RNA sequencing analysis of FGF2-responsive transcriptome in skin fibroblasts. PeerJ 9, e10671. 10.7717/peerj.10671 33520460 PMC7812929

[B306] Wu Y. Wu Y. Yu J. Zhang Y. Li Y. Fu R. (2023). Irisin ameliorates D-galactose-induced skeletal muscle fibrosis *via* the PI3K/Akt pathway. Eur. J. Pharmacol. 939, 175476. 10.1016/j.ejphar.2022.175476 36539073

[B307] Wu D. Zhao X. Xie J. Yuan R. Li Y. Yang Q. (2024). Physical modulation of mesenchymal stem cell exosomes: a new perspective for regenerative medicine. Cell Prolif. 57, e13630. 10.1111/cpr.13630 38462759 PMC11294442

[B308] Wynn T. A. (2008). Cellular and molecular mechanisms of fibrosis. J. Pathol. 214, 199–210. 10.1002/path.2277 18161745 PMC2693329

[B309] Xia Y. Entman M. L. Wang Y. (2013). Critical role of CXCL16 in hypertensive kidney injury and fibrosis. Hypertension 62, 1129–1137. 10.1161/HYPERTENSIONAHA.113.01837 24060897 PMC3926124

[B310] Xia Y. Jin X. Yan J. Entman M. L. Wang Y. (2014). CXCR6 plays a critical role in angiotensin II-induced renal injury and fibrosis. Arterioscler. Thromb. Vasc. Biol. 34, 1422–1428. 10.1161/ATVBAHA.113.303172 24855055 PMC4089979

[B311] Xiao Y. (2021). MiR-486-5p inhibits the hyperproliferation and production of collagen in hypertrophic scar fibroblasts *via* IGF1/PI3K/AKT pathway. J. Dermatol. Treat. 32, 973–982. 10.1080/09546634.2020.1728210 32079424

[B312] Xiao S. Zhang M. Liang Y. Wang D. (2017). Celastrol synergizes with oral nifedipine to attenuate hypertension in preeclampsia: a randomized, placebo-controlled, and double blinded trial. J. Am. Soc. Hypertens. 11, 598–603. 10.1016/j.jash.2017.07.004 28757108

[B313] Xiao Y. Tang Z. Huang X. Chen W. Zhou J. Liu H. (2022). 'Emerging mRNA technologies: delivery strategies and biomedical applications. Chem. Soc. Rev. 51, 3828–3845. 10.1039/d1cs00617g 35437544

[B314] Xie T. Xu Q. Wan H. Xing S. Shang C. Gao Y. (2019). 'Lipopolysaccharide promotes lung fibroblast proliferation through autophagy inhibition *via* activation of the PI3K-Akt-mTOR pathway. Lab. Invest 99, 625–633. 10.1038/s41374-018-0160-2 30760865

[B315] Xin Y. Min P. Xu H. Zhang Z. Zhang Y. Zhang Y. (2020). CD26 upregulates proliferation and invasion in keloid fibroblasts through an IGF-1-induced PI3K/AKT/mTOR pathway. Burns Trauma 8, tkaa025. 10.1093/burnst/tkaa025 33150188 PMC7596300

[B316] Xiu Y. Su Y. Gao L. Yuan H. Xu S. Liu Y. (2023). 'Corylin accelerated wound healing through SIRT1 and PI3K/AKT signaling: a candidate remedy for chronic non-healing wounds. Front. Pharmacol. 14, 1153810. 10.3389/fphar.2023.1153810 37266148 PMC10229780

[B317] Xu Z. Hu B. Zheng G. Yu W. Yang C. Wang H. (2024). Metformin-grafted polycaprolactone nanoscaffold targeting sensory nerve controlled fibroblasts reprograming to alleviate epidural fibrosis. J. Control Release 367, 791–805. 10.1016/j.jconrel.2024.02.001 38341179

[B318] Xu Z. Liu L. Dai X. Zhou X. Chen L. Chen H. (2025). '5'tiRNA-Glu-TTC targets TRPV3 and activates the PI3K/AKT signaling pathway to modulate skin photoaging. Noncoding RNA Res. 15, 29–43. 10.1016/j.ncrna.2025.07.004 40741358 PMC12310068

[B319] Xue X. Ling X. Xi W. Wang P. Sun J. Yang Q. (2020). Exogenous hydrogen sulfide reduces atrial remodeling and atrial fibrillation induced by diabetes mellitus *via* activation of the PI3K/Akt/eNOS pathway. Mol. Med. Rep. 22, 1759–1766. 10.3892/mmr.2020.11291 32705232 PMC7411292

[B320] Xue W. Sun R. Hao Z. Xing Z. Cheng H. Shao L. (2023). Tetrandrine inhibits migration and invasion of BGC-823 and MKN-45 cells by regulating PI3K/AKT/mTOR signaling pathway. Chem. Biol. Drug Des. 101, 927–936. 10.1111/cbdd.14202 36593659

[B321] Yan Y. Zhou M. Meng K. Zhou C. Jia X. Li X. (2023). 'Salvianolic acid B attenuates inflammation and prevent pathologic fibrosis by inhibiting CD36-mediated activation of the PI3K-Akt signaling pathway in frozen shoulder. Front. Pharmacol. 14, 1230174. 10.3389/fphar.2023.1230174 37593175 PMC10427508

[B322] Yang B. Y. Zhou Z. Y. Liu S. Y. Shi M. J. Liu X. J. Cheng T. M. (2022). 'Porous Se@SiO(2) nanoparticles enhance wound healing by ROS-PI3K/Akt pathway in dermal fibroblasts and reduce scar formation. Front. Bioeng. Biotechnol. 10, 852482. 10.3389/fbioe.2022.852482 35387298 PMC8978548

[B323] Yang C. Bachu M. Du Y. Brauner C. Yuan R. Ah Kioon M. D. (2022). CXCL4 synergizes with TLR8 for TBK1-IRF5 activation, epigenomic remodeling and inflammatory response in human monocytes. Nat. Commun. 13, 3426. 10.1038/s41467-022-31132-7 35701499 PMC9195402

[B324] Yang L. Xie F. Li Y. Lu Y. Li B. Hong S. (2023). 'Chitin-based hydrogel loaded with bFGF and SDF-1 for inducing endogenous mesenchymal stem cells homing to improve stress urinary incontinence. Carbohydr. Polym. 319, 121144. 10.1016/j.carbpol.2023.121144 37567701

[B325] Yang D. Li W. Xiang P. Ge T. Li H. Zhang Y. (2024). 'Rhein promotes skin wound healing by activating the PI3K/AKT signaling pathway. Open Med. (Wars) 19, 20241116. 10.1515/med-2024-1116 39726811 PMC11669899

[B326] Yang J. Zhang Y. Liang J. Yang X. Liu L. Zhao H. (2022). Fibronectin-1 is a dominant mechanism for rheumatoid arthritis *via* the mediation of synovial fibroblasts activity. Front. Cell Dev. Biol. 10, 1010114. 10.3389/fcell.2022.1010114 36225320 PMC9548557

[B327] Yang R. Tang Y. Hou J. Yu M. Long Y. Yamuhanmode A. (2022). 'Fibrosis in frozen shoulder: activation of IL-6 through PI3K-Akt signaling pathway in synovial fibroblast. Mol. Immunol. 150, 29–38. 10.1016/j.molimm.2022.07.007 35930846

[B328] Yao F. Xu M. Dong L. Shen X. Shen Y. Jiang Y. (2024). Sinomenine attenuates pulmonary fibrosis by downregulating TGF-β1/Smad3, PI3K/Akt and NF-κB signaling pathways. BMC Pulm. Med. 24, 229. 10.1186/s12890-024-03050-5 38730387 PMC11088103

[B329] Yap T. A. Krebs M. G. Postel-Vinay S. El-Khouiery A. Soria J. C. Lopez J. (2021). 'Ceralasertib (AZD6738), an oral ATR kinase inhibitor, in combination with carboplatin in patients with advanced solid tumors: a phase I Study. Clin. Cancer Res. 27, 5213–5224. 10.1158/1078-0432.CCR-21-1032 34301752 PMC9401487

[B330] Yin Y. Han Y. Shi C. Xia Z. (2020). IGF-1 regulates the growth of fibroblasts and extracellular matrix deposition in pelvic organ prolapse. Open Med. (Wars) 15, 833–840. 10.1515/med-2020-0216 33336041 PMC7712242

[B331] You H. Gou Q. Dong M. Chang F. Xiu J. (2024). Exploring the role of iNOS in HFpEF-Related myocardial fibrosis: involvement of PTEN-PI3K/AKT signaling pathway. Biochem. Biophys. Res. Commun. 734, 150589. 10.1016/j.bbrc.2024.150589 39245028

[B332] Younesi F. S. Miller A. E. Barker T. H. Rossi F. M. V. Hinz B. (2024). 'Fibroblast and myofibroblast activation in normal tissue repair and fibrosis. Nat. Rev. Mol. Cell Biol. 25, 617–638. 10.1038/s41580-024-00716-0 38589640

[B333] Yu M. Shi J. Sheng M. Gao K. Zhang L. Liu L. (2018). Astragalus inhibits epithelial-to-mesenchymal transition of peritoneal mesothelial cells by down-regulating β-Catenin. Cell Physiol. Biochem. 51, 2794–2813. 10.1159/000495972 30562743

[B334] Yu B. Cao Y. Lin P. Zhang L. Chen M. (2025). Enhancement of Ndrg2 promotes hypertrophic scar fibrosis by regulating PI3K/AKT signaling pathway. Cell Signal 129, 111659. 10.1016/j.cellsig.2025.111659 39956247

[B335] Yu L. Wei J. Liu P. (2022). Attacking the PI3K/Akt/mTOR signaling pathway for targeted therapeutic treatment in human cancer. Semin. Cancer Biol. 85, 69–94. 10.1016/j.semcancer.2021.06.019 34175443

[B336] Yu X. Xiao Q. Yu X. Cheng Y. Lin H. Xiang Z. (2022). A network pharmacology-based study on the mechanism of astragaloside IV alleviating renal fibrosis through the AKT1/GSK-3β pathway. J. Ethnopharmacol. 297, 115535. 10.1016/j.jep.2022.115535 35840059

[B337] Zeng M. Y. Pham D. Bagaitkar J. Liu J. Otero K. Shan M. (2013). 'An efferocytosis-induced, IL-4-dependent macrophage-iNKT cell circuit suppresses sterile inflammation and is defective in murine CGD. Blood 121, 3473–3483. 10.1182/blood-2012-10-461913 23426944 PMC3637016

[B338] Zha Y. Li Y. Ge Z. Wang J. Jiao Y. Zhang J. (2022). ADAMTS8 promotes cardiac fibrosis partly through activating EGFR dependent pathway. Front. Cardiovasc Med. 9, 797137. 10.3389/fcvm.2022.797137 35224040 PMC8866452

[B339] Zhang X. L. Xing R. G. Chen L. Liu C. R. Miao Z. G. (2016). PI3K/Akt signaling is involved in the pathogenesis of bleomycin-induced pulmonary fibrosis *via* regulation of epithelial-mesenchymal transition. Mol. Med. Rep. 14, 5699–5706. 10.3892/mmr.2016.5960 27878273

[B340] Zhang W. Zhang Y. Tu T. Schmull S. Han Y. Wang W. (2020). 'Dual inhibition of HDAC and tyrosine kinase signaling pathways with CUDC-907 attenuates TGFβ1 induced lung and tumor fibrosis. Cell Death Dis. 11, 765. 10.1038/s41419-020-02916-w 32943605 PMC7499263

[B341] Zhang J. Zhong W. Liu Y. Chen W. Lu Y. Zeng Z. (2021). Extracellular HSP90α interacts with ER stress to promote fibroblasts activation through PI3K/AKT pathway in pulmonary fibrosis. Front. Pharmacol. 12, 708462. 10.3389/fphar.2021.708462 34497513 PMC8420756

[B342] Zhang Q. Chen W. W. Sun X. Qian D. Tang D. D. Zhang L. L. (2022a). The versatile emodin: a natural easily acquired anthraquinone possesses promising anticancer properties against a variety of cancers. Int. J. Biol. Sci. 18, 3498–3527. 10.7150/ijbs.70447 35637953 PMC9134920

[B343] Zhang Q. Qian D. Tang D. D. Liu J. Wang L. Y. Chen W. (2022b). Glabridin from Glycyrrhiza glabra possesses a therapeutic role against keloid *via* attenuating PI3K/Akt and transforming growth Factor-β1/SMAD signaling pathways. J. Agric. Food Chem. 70, 10782–10793. 10.1021/acs.jafc.2c02045 36005946

[B344] Zhang X. Guan X. Piao Y. Che X. Si M. Jin J. (2022c). Baicalein induces apoptosis of rheumatoid arthritis synovial fibroblasts through inactivation of the PI3K/Akt/mTOR pathway. Evid. Based Complement. Altern. Med. 2022, 3643265. 10.1155/2022/3643265 36118088 PMC9473868

[B345] Zhang J. Yu H. Man M. Q. Hu L. (2024). Aging in the dermis: fibroblast senescence and its significance. Aging Cell 23, e14054. 10.1111/acel.14054 38040661 PMC10861215

[B346] Zhang F. Geng L. Zhang J. Han S. Guo M. Xu Y. (2025). 'miR-486-5p diagnosed atrial fibrillation, predicted the risk of left atrial fibrosis, and regulated angiotensin II-induced cardiac fibrosis *via* modulating PI3K/Akt signaling through targeting FOXO1. Mol. Cell Biochem. 480, 1077–1087. 10.1007/s11010-024-05027-8 38782834

[B347] Zhang L. Wang X. He S. Zhang F. Li Y. (2023). 'Gypenosides suppress fibrosis of the renal NRK-49F cells by targeting miR-378a-5p through the PI3K/AKT signaling pathway. J. Ethnopharmacol. 311, 116466. 10.1016/j.jep.2023.116466 37031821

[B348] Zhang P. Li H. Zhang A. Wang X. Song Q. Li Z. (2023). 'Mechanism of myocardial fibrosis regulation by IGF-1R in atrial fibrillation through the PI3K/Akt/FoxO3a pathway. Biochem. Cell Biol. 101, 432–442. 10.1139/bcb-2022-0199 37018819

[B349] Zhang Q. Kuang G. Li W. Wang J. Ren H. Zhao Y. (2023). Stimuli-Responsive gene delivery nanocarriers for cancer therapy. Nanomicro Lett. 15, 44. 10.1007/s40820-023-01018-4 36752939 PMC9908819

[B350] Zhang X. Zhang Y. Liu Y. (2025). 'Fibroblast activation and heterogeneity in fibrotic disease. Nat. Rev. Nephrol. 21, 613–632. 10.1038/s41581-025-00969-8 40537561

[B351] Zhao H. Li C. Li L. Liu J. Gao Y. Mu K. (2020a). Baicalin alleviates bleomycin-induced pulmonary fibrosis and fibroblast proliferation in rats *via* the PI3K/AKT signaling pathway. Mol. Med. Rep. 21, 2321–2334. 10.3892/mmr.2020.11046 32323806 PMC7185294

[B352] Zhao H. Wang Y. Qiu T. Liu W. Yao P. (2020b). 'Autophagy, an important therapeutic target for pulmonary fibrosis diseases. Clin. Chim. Acta 502, 139–147. 10.1016/j.cca.2019.12.016 31877297

[B353] Zhao Y. Qu Y. Hao C. Yao W. (2023). PD-1/PD-L1 axis in organ fibrosis. Front. Immunol. 14, 1145682. 10.3389/fimmu.2023.1145682 37275876 PMC10235450

[B354] Zhao S. Kong H. Qi D. Qiao Y. Li Y. Cao Z. (2025). Epidermal stem cell derived exosomes-induced dedifferentiation of myofibroblasts inhibits scarring *via* the miR-203a-3p/PIK3CA axis. J. Nanobiotechnology 23, 56. 10.1186/s12951-025-03157-9 39881312 PMC11776291

[B355] Zhao S. Liu H. Wang H. He X. Tang J. Qi S. (2024). 'Inhibition of phosphatidylinositol 3-kinase catalytic subunit alpha by miR-203a-3p reduces hypertrophic scar formation *via* phosphatidylinositol 3-kinase/AKT/mTOR signaling pathway. Burns Trauma 12, tkad048. 10.1093/burnst/tkad048 38179473 PMC10762504

[B356] Zhao Y. Qi Y. Xia J. Duan M. Hao C. Yao W. (2024). The role of the PI3K/AKT/mTOR pathway in mediating PD-L1 upregulation during fibroblast transdifferentiation. Int. Immunopharmacol. 142, 113186. 10.1016/j.intimp.2024.113186 39298817

[B357] Zhi Y. Wang H. Huang B. Yan G. Yan L. Z. Zhang W. (2021). Panax Notoginseng saponins suppresses TRPM7 *via* the PI3K/AKT pathway to inhibit hypertrophic scar formation *in vitro* . Burns 47, 894–905. 10.1016/j.burns.2020.10.003 33143990

[B358] Zhong S. Guo H. Wang H. Xing D. Lu T. Yang J. (2020). Apelin-13 alleviated cardiac fibrosis *via* inhibiting the PI3K/Akt pathway to attenuate oxidative stress in rats with myocardial infarction-induced heart failure. Biosci. Rep. 40. 10.1042/BSR20200040 32207519 PMC7133518

[B359] Zhou Y. Ren H. Dai B. Li J. Shang L. Huang J. (2018). 'Hepatocellular carcinoma-derived exosomal miRNA-21 contributes to tumor progression by converting hepatocyte stellate cells to cancer-associated fibroblasts. J. Exp. Clin. Cancer Res. 37, 324. 10.1186/s13046-018-0965-2 30591064 PMC6307162

[B360] Zhou Z. Liang S. Zhou Z. Liu J. Zhang J. Meng X. (2023). TGF-β1 promotes SCD1 expression *via* the PI3K-Akt-mTOR-SREBP1 signaling pathway in lung fibroblasts. Respir. Res. 24, 8. 10.1186/s12931-023-02313-9 36627645 PMC9832654

[B361] Zhou C. Cai Z. Guo J. Li C. Qin C. Yan J. (2024). Injective hydrogel loaded with liposomes-encapsulated MY-1 promotes wound healing and increases tensile strength by accelerating fibroblast migration *via* the PI3K/AKT-Rac1 signaling pathway. J. Nanobiotechnology 22, 396. 10.1186/s12951-024-02666-3 38965546 PMC11225333

[B362] Zhu N. Li T. Bai Y. Sun J. Guo J. Yuan H. (2024). Targeting myocardial inflammation: investigating the therapeutic potential of atrial natriuretic peptide in atrial fibrosis. Mol. Biol. Rep. 51, 506. 10.1007/s11033-024-09393-w 38622341 PMC11018689

[B363] Ziegler E. (1900). General pathology: or, the science of the causes, nature and course of the pathological disturbances which occur in the living subject. New York, United States: William Wood and Company.

[B364] Zou A. E. Kongthong S. Mueller A. A. Brenner M. B. (2025). Fibroblasts in immune responses, inflammatory diseases and therapeutic implications. Nat. Rev. Rheumatol. 21, 336–354. 10.1038/s41584-025-01259-0 40369134

[B365] Zymek P. Bujak M. Chatila K. Cieslak A. Thakker G. Entman M. L. (2006). The role of platelet-derived growth factor signaling in healing myocardial infarcts. J. Am. Coll. Cardiol. 48, 2315–2323. 10.1016/j.jacc.2006.07.060 17161265

